# Modelling the effects of quadrivalent Human Papillomavirus (HPV) vaccination in Puerto Rico

**DOI:** 10.1371/journal.pone.0184540

**Published:** 2017-11-30

**Authors:** Ana Patricia Ortiz, Karen J. Ortiz-Ortiz, Moraima Ríos, José Laborde, Amit Kulkarni, Matthew Pillsbury, Andreas Lauschke, Homero A. Monsanto, Cecile Marques-Goyco

**Affiliations:** 1 Cancer Control and Population Sciences Program, University of Puerto Rico Comprehensive Cancer Center, San Juan, Puerto Rico; 2 Graduate School of Public Health, University of Puerto Rico, Medical Sciences Campus, San Juan, Puerto Rico; 3 Department of Economics, University of Puerto Rico, Rio Piedras Campus, San Juan, Puerto Rico; 4 Merck & Co., Inc., Kenilworth, New Jersey, United States of America; 5 Lauschke Consulting, New York, New York, United States of America; 6 Health Outcomes Research Regional Lead, Latin America Medical Affairs, Merck & Co, San Juan, Puerto Rico; Universidad Nacional de la Plata, ARGENTINA

## Abstract

**Background:**

No study has estimated the potential impact of Human Papillomavirus (HPV) vaccination in Puerto Rico, a population with considerable burden of HPV-related morbidities. We evaluated the health and economic impacts of implementing a vaccination strategy for females and males in Puerto Rico, with the quadrivalent HPV (HPV4) vaccine, under different vaccination scenarios.

**Methods:**

We adapted a mathematical model which estimates the direct and indirect health benefits and costs of HPV4 vaccination in a dynamic population. The model compared three vaccination scenarios against screening only (no-vaccination) for three doses of HPV4 vaccine among individuals aged 11–15 years in Puerto Rico: 1) 34% for females and 13% for males (34%F/13%M), 2) 50% for females and 40% for males (50%F/40%M), and 3) 80% for female and 64% for male (80%F/64%M). Data specific to Puerto Rico was used. When not available, values from the United States were used. Input data consisted of demographic, behavioral, epidemiological, screening, and economic parameters.

**Results:**

The model predicted decreases in: 1) HPV infection prevalence for females and males, 2) cervical intraepithelial neoplasia and cervical cancer incidence for females, 3) genital warts incidence for females and males, and 4) cervical cancer deaths among females, when various vaccination program scenarios were considered. In addition, when the vaccination percentage was increased in every scenario, the reduction was greater and began earlier. The analysis also evidenced an incremental cost effectiveness ratio (ICER) of $1,964 per quality–adjusted life year gained for the 80%F/64%M uptake scenario.

**Conclusions:**

HPV vaccine can prove its cost effectiveness and substantially reduce the burden and costs associated to various HPV-related conditions when targeted to the adequate population together with an organized HPV vaccination program.

## Introduction

Human Papillomavirus (HPV) is a DNA virus with the ability to infect epithelial cells or mucous membranes in humans [1]. Over 100 genotypes have been identified, and worldwide it is considered the most common sexually transmitted infection (STI) [2]. Some HPV genotypes are considered low-risk (e.g. HPV-6 and 11), and others high-risk (e.g. HPV-16 and 18) [3]. The low-risk genotypes are known for causing skin warts and mucosal papilloma in the anogenital region [3]. HPV 6 and 11 account for over 90% of genital warts [4]. High-risk HPV genotypes are oncogenic, and HPV 16, 18, 31, 33, 45 are most commonly associated with cervical intraepithelial neoplasia [CIN], carcinoma in situ and invasive cancers [5]. It is the persistence of an oncogenic genotype (e.g. HPV 16, 18, 31, 33, 35 45, 51, 52, 58) that is strongly linked to pre-cancer [2]. The most common areas affected by high-risk HPV genotypes are cervix, anus, penis, vagina, vulva, and oropharynx [6]. HPV DNA is found in 99.7% of cervical carcinomas, with 70% of them containing HPV 16 or 18 [2].

Worldwide, cervical cancer is the second leading cause of cancer death in women between 15 and 44 years of age [7]. HPV is estimated to cause over 527,000 incident cases of cervical cancer and over 265,000 deaths worldwide, with 84% of the cases occurring in less developed regions [7]. In Puerto Rico, cancer was the second leading cause of death by 2010 [8], and cervical cancer accounts for 3.9% of all cancer deaths [9].

On the other hand, genital warts are the leading STI in the United States (US), particularly among young adults [10]. As genital warts in Puerto Rico are underreported, incidence data likely underestimate the true occurrence [11]. Although the prevalence of genital warts is not high, and they are not fatal, they can have a significant psychological burden and result in high healthcare costs [4].

Research in the US, Mexico, United Kingdom, Colombia, and Italy has documented the economic impact of HPV-related morbidities and the cost effectiveness that HPV vaccination could have in these populations [12–17]. It is documented by Seto and colleagues that the inclusion of boys in the vaccination program is more cost-effective than girls only. However, the authors note that due to a absence of agreement on the proper thresholds for cost effectiveness, comparison of results across studies with regard to boys’ vaccination was limited [18].

The importance of HPV in the etiology of cervical cancer and other HPV-related morbidities and the needs for preventive measures gave rise to the development of HPV vaccines [19]. At present, there are three vaccines approved by the FDA to prevent HPV-related diseases. GARDASIL, approved in 2006, is a quadrivalent (HPV4) vaccine that provides efficacy against two high-risk HPV genotypes, HPV 16 and 18, as well as two low-risk HPV genotypes, HPV 6 and 11, and is expected to prevent 70% of cervical cancers. It is indicated for females and males 9 to 26 years old [20]. CERVARIX, approved in 2009, is a bivalent (HPV2) vaccine against HPV-16 and HPV-18, indicated for females aged 9 to 25 years old [21]. More recently, in 2014, the Food and Drug Administration approved GARDASIL-9 (HPV9) that includes five additional oncogenic genotypes (31, 33, 45, 52, and 58) to the original four genotypes in Gardasil (6, 11, 16, and 18) and is indicated for females 9 to 26 years of age and males 9 to 15 years of age [22]. The HPV9 vaccine will increase prevention of cervical cancer to 90%. [23]. Even though the vaccination program can begin at age 9, the Advisory Committee for Immunization Practices (ACIP) suggests a vaccination regimen with HPV2 or HPV4 for females ages 11 or 12 and HPV4 for males. GARDASIL-9 has been included to the routine vaccination recommendations for 11 and 12 years old. In the US, all HPV vaccines are recommended in a three-dose schedule [24]. Although the vaccines have been proven to be effective against the above-mentioned HPV types, these vaccines do not replace the pap smear screening test, and women are still recommended to be tested [20–22, 24].

In spite of the existence and approval of HPV vaccines, vaccination rates are low. In Puerto Rico, data from the 2014 National Immunization Survey–Teenshow that HPV-vaccination coverage rates for the 3 dose-schedule were estimated at 49.9% of females and 23.7% of males ages 13–17; this estimate is higher than for the US, where 39.7% of females and 21.6% of males of the same ages have completed the vaccination regimen [25]. Nonetheless, all these estimates are well below the 2020 goal of 80% established for girls [26]. Improving the vaccination rates would not only protect the person who receives the vaccine but would also help to reduce HPV disease incidence through herd immunity. This has been documented in Australia where, incidence of genital warts declined in both young women and heterosexuals after a vaccination program was introduced [27, 28].

Data on the public health and economic consequences of HPV vaccination in Puerto Rico is of great relevance to guide public policy and other prevention and control efforts for HPV related disease. It is imperative to compare the costs and benefits of introducing a vaccination plan along with the current screening program versus screening only (no-vaccination) plan, taking into consideration different vaccination coverage rates. Therefore, the aim of this analysis was to adapt a previously published economic model to evaluate the health and economic impacts in Puerto Rico of implementing an HPV4 vaccination strategy for females and males, 11–15 years of age, under different vaccination scenarios compared to a no-vaccination strategy. In addition, to estimate the HPV4 vaccine potential effect on HPV associated diseases and cervical cancer mortality, and to evaluate HPV4 vaccination cost-effectiveness utilizing a variety of vaccination scenarios from Puerto Rico’s healthcare point of view. This is one of the few instances where the model has been adapted to demonstrate the potential impact of increasing vaccination rates on the prevention of HPV-related disease.

## Methods

A previously published transmission dynamic model [29–31] for HPV genotypes 6, 11, 16, 18 was adapted to Puerto Rico. The model is an age-structured mathematical model that incorporates: 1) a demographic model describing birth, aging, and death, 2) a behavioral model describing sexual mixing patterns, and 3) HPV infection and disease models describing transmission and disease occurrences. The model shows the vaccine direct and indirect effects (herd immunity). Although three types of HPV mathematical models have been reported in the literature (cohort, population dynamic, and hybrid), only the population dynamic model can account for both the direct and indirect (i.e., herd immunity effects) benefits of vaccination in the population [29]. The model that was used in this adaptation was calibrated and validated by the developers in three steps [30]. First, they established the face validity of the model by consulting with experts on assumptions regarding the natural history of HPV infection and disease and vaccine characteristics. Second, the predictive validity of the model was evaluated by comparing model results to epidemiologic data reported in the literature. They evaluated the model predictions against the following outcomes: HPV prevalence; genital wart incidence; RRP incidence; CIN 1, 2, 3 incidences; and incidences of cervical, vaginal, vulvar, anal, penile, head/neck cancer and death due to each of these cancers. Model prediction generally fell within the range of values reported in the literature. Finally, they assessed the convergent validity of the model by comparing estimates of the cost-effectiveness of HPV vaccination with those of several previously published studies. The range of ICER estimates from these studies overlapped many of the ranges of estimates reported in their results. Qualitatively their findings were also similar to previous studies for adolescent female HPV vaccination they reviewed. [30] Furthermore, a more recent systematic review and meta-analysis of model predictions of the long-term population-level effectiveness of vaccination against HPV 16, 18, 6, and 11 infection in women and men, to examine the variability in predicted herd effects, incremental benefit of vaccinating boys, and potential for HPV-vaccine-type elimination found that the population-level predictions were concordant across different transmission-dynamic mathematical models [32].

A literature review was performed to attain the model parameter values, which included demographic, sexual behavior and screening variables. Population estimates came from the U.S. Census Bureau [33] and screening information (% of females receiving at least 1 pap every 3 years and cervical cancer screening rates by age) were obtained from the Puerto Rico Behavioral Risk Factor Surveillance Survey (BRFSS) [34]. Sexual behavior (Mean number of sexual partners) was assessed from population-based studies in Puerto Rico [35,36], and information on cervical cancer incidence as well as cervical cancer and all-cause mortality came from the Puerto Rico Central Cancer Registry [37, 38]. Some model inputs, such as genital warts prevalence and attributable risk, were estimates based on the assumed rate of genital warts infection transmission from previous model reports [13]. Direct medical costs for HPV-related diseases were estimated from private insurance claims data (using ICD 9 and CPT codes); therefore, the perspective of the analysis is that of the private sector.The model compared three gender neutral vaccination scenarios and cervical cancer screening against cervical cancer screening alone (no-vaccination) for three doses of HPV4 vaccine among individuals aged 11–15 years old in Puerto Rico:

1. 34% for females and 13% for males (34%F/13%M)
2. 50% for females and 40% for males (50%F/40%M)
3. 80% for female and 64% for male (80%F/64%M)

The model assumed the following vaccine degree of protection:

Against transient infection for females (any time DNA detection): HPV16: 76.0%; HPV18: 96.3%; HPV6: 76.1%; HPV11: 76.1%

- Against persistent infection for females: HPV16: 98.8%; HPV18: 98.4%
- Against individual diseases:
  ○ CIN: HPV16: 97.9%; HPV18: 100%; HPV6: 100%; HPV11: 100%
  ○ Genital warts for females: HPV6: 98.9%; HPV11: 100%
  ○ Genital warts for males: HPV6: 84.3%; HPV11: 90.9%

Furthermore, the model assumed a lifelong protection and that the subjects adhered to the full three-dose vaccination regimen.

## Results

**Fig 1:**
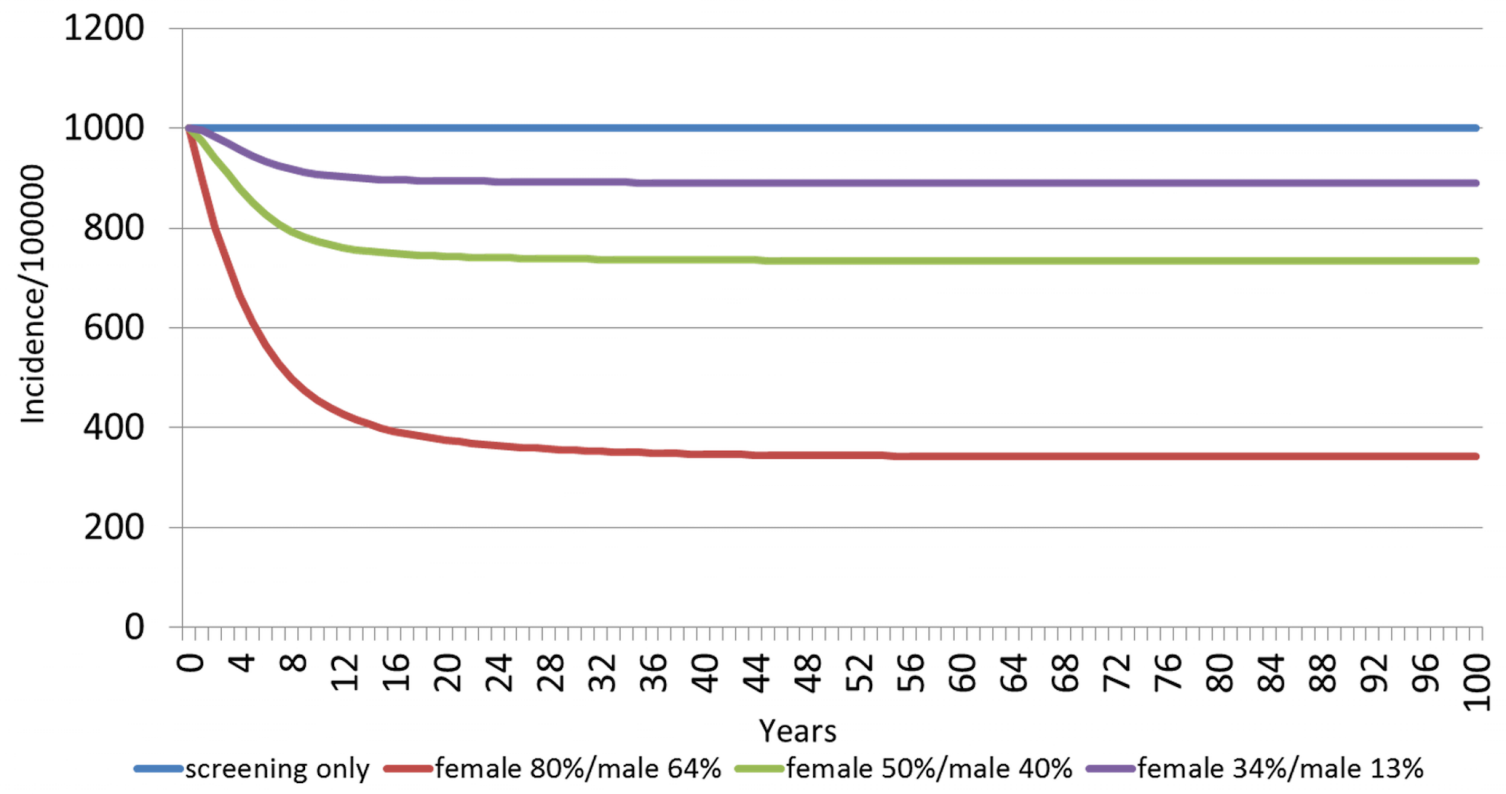
Estimated HPV 6/11 related incidence of genital warts among females over 100 years, by vaccination scenario.

**Fig 2:**
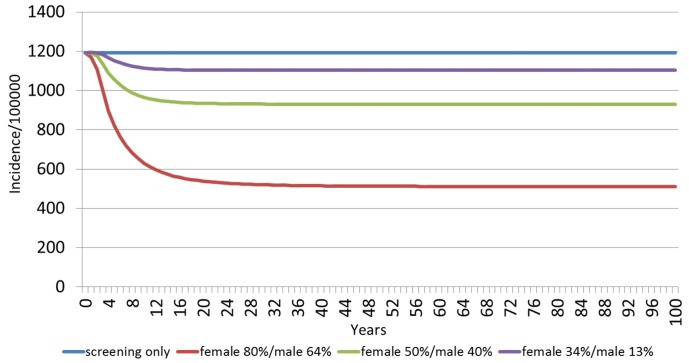
Estimated HPV 6/11 related incidence of genital warts among males over 100 years, by vaccination scenario.

The estimated HPV 6/11-related incidences of genital warts among females and males over a 100 year timespan, grouped by vaccination strategy, are shown in Figs 1 and 2, respectively. For females, the baseline annual incidence of HPV 6/11-related incidence of genital warts is 1,001 per 100,000 people, with screening, and no vaccination (Fig 1). With the 34%F/13%M vaccination scenario, the model predicts a reduction in incidence to 898 cases per 100,000 people after 15 years. Increasing the vaccination coverage to 50%F/40%M would further reduce the incidence at 15 years to 751 per 100,000 people, while for the 80% F/64%M scenario it would reach the lowest incidence rate of all scenarios of 399 per 100,000 people after 15 years. For males, the baseline annual incidence of HPV 6/11-related incidence of genital warts was 1,193 per 100,000 people with screening, and no vaccination (Fig 2). As shown, the reduction of genital warts incidence among males occurs faster than among females. With the 34%F/13%M vaccination scenario for females and males, reductions in incidence rates peak at 10 years (1,115 per 100,000). Increasing the vaccination coverage to 50%F/40%M for females and males will decrease the incidence rate to 966 per 100,000 people. In the 80%F/64%M vaccination scenario, the incidence rate would be further reduced to 630 per 100,000 people.

**Fig 3:**
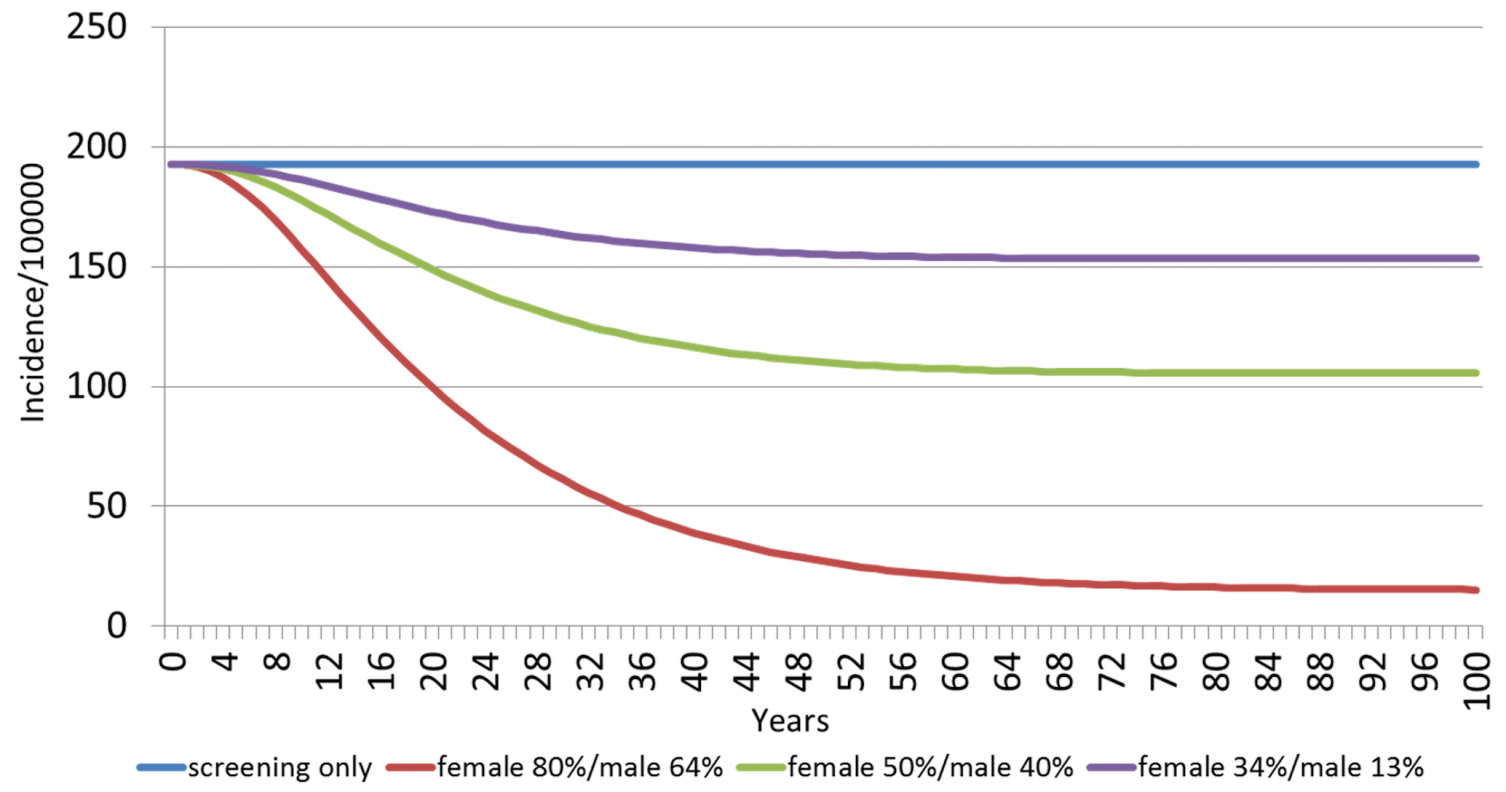
Estimated HPV 16/18 related incidence of CIN 2/3 among females over 100 years, by vaccination scenario.

Fig 3 shows the estimated HPV 16/18-related incidence of CIN 2/3 among females over 100 years, by vaccination strategy. With screening only, the annual incidence of HPV 16/18-related CIN 2/3 is 193 per 100,000 females. The model projects dramatic reductions of CIN2/3 at 20, 40 and 60 years as vaccination coverage increases. The incidence per 100,000 females at 20 years in the 34%F/13%M scenario is 173, at 40 years is 158, and at 60 years is 154. With the vaccination scenarios of 50%F/40%M and 80%F/64%M, the incidence rate for CIN2/3 is 149 and 100 at 20 years, 116 and 39 at 40 years, and 107 and 21 at 60 years, respectively.

**Fig 4:**
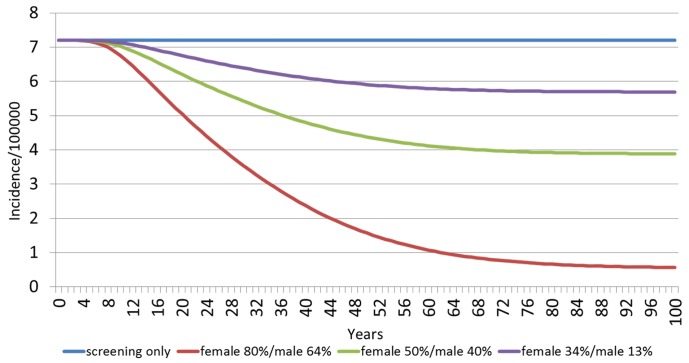
Estimated HPV 16/18 related incidence of cervical cancer among females over 100 years, by vaccination scenario.

Fig 4 shows the estimated HPV 16/18-related incidences of cervical cancer among females over a 100 year time horizon, by vaccination scenario. Since cervical cancer has a slow progression, the effects of the vaccination strategies in the decline of the incidences take more time than with other HPV-related diseases. The model predicts an annual incidence of 7.2 per 100,000 people with screening, and no vaccination. With the current vaccination percentage of 34%F/13%M in 20 years it would present a slight reduction in incidence rate to 6.2 per 100,000, in 40 years to 6.1 per 100,000, and in 60 years to 5.8 per 100,000. If the vaccination coverage was increased to 50%F/40%M, the incidence rate would decrease to 6.2 per 100,000 in 20 years, 4.8 per 100,000 in 40 years, and 4.2 per 100,000 in 60 years. If the 80%F/64%M vaccination coverage were achieved, in the first 20 years the cervical cancer incidence rate would be dramatically reduced to 5.0 per 100,000, followed by a reduction to 2.4 per 100,000 in 40 years, and 1.1 per 100,000 in 60 years.

**Fig 5:**
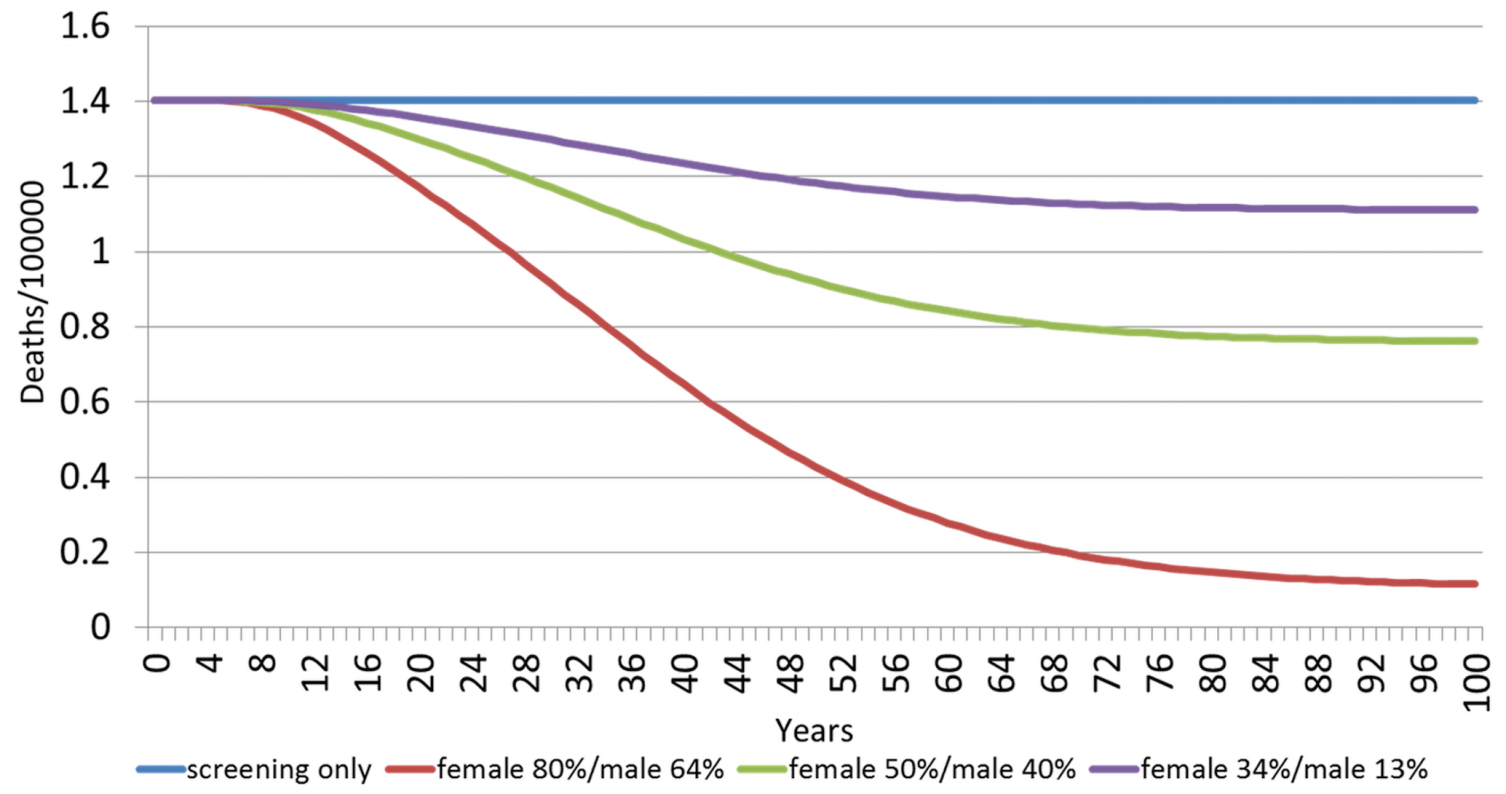
Estimated HPV 16/18 related cervical cancer deaths among females over 100 years, by vaccination scenario.

Fig 5 shows the estimated HPV 16/18-related cervical cancer deaths among females over a 100 year time horizon, by vaccination scenario. The model predicts an annual death rate of HPV 16/18-related cervical cancer of 1.4 with screening, and no vaccination. With the current vaccination percentage of 34%F/13%M, in 20 years it would present a small reduction to 1.4 per 100,000, in 60 years to 1.1 per 100,000 and then it would continue steady over the next 40 years. If the vaccination percentage were to increase to 50%F/40%M, the death rate will present a reduction to 1.3 per 100,000 in 20 years, at 60 years it would go to 0.8 per 100,000 and at 100 years it would be around 0.8 per 100,000. If the 80%F/64%M vaccination coverage was achieved, on the first 20 years the cervical cancer death rate will dramatically be reduced to 1.2 per 100,000, and in 60 years to 0.3 per 100,000.

Meanwhile, Table 1 shows the estimated cumulative HPV genotypes 6, 11, 16, 18-related disease incidence costs avoided at the population level, grouped by vaccination scenario. If the 34%F/13%M vaccination scenario is considered, the estimated costs avoided for genital warts for females and males go from $8.6 million and $8.1 million, respectively, in 25 years to $14.3 million and $13.4 million, respectively, in 50 years. The CIN1 estimated cost reduction under this vaccination coverage scenario more than doubles, from $841,749 in 25 years to nearly $2 million in 50 years. Meanwhile, in CIN2/3 we see a cost reduction of $4.8 million in 25 years and of $13.4 million in 50 years. The cervical cancer presents an estimated cost reduction of $1.2 million in the first 25 years and $4.3 million in 50 years. Under the 34%F/13%M vaccination scenario, all these disease costs sum up to a total direct cost avoidance of $23.7 million in 25 years and $47.4 million in 50 years.

**Table 1:** Estimated cumulative incidence costs avoided since HPV4 vaccination program started, by vaccination scenario.

HPV Disease	Vaccination Scenario (34% Female/13% Male)			Vaccination Scenario (50% Female/40% Male)			Vaccination Scenario (80% Female/64% Male)		
	25 years	50 years	100 years	25 years	25 years	50 years	100 years	50 years	25 years
Genital warts-female	$8,671,954	$14,257,263	$18,123,487	Genital warts-female	$8,671,954	$14,257,263	$18,123,487	Genital warts-female	$8,671,954
Genital warts-male	$8,108,461	$13,440,910	$17,091,889	Genital warts-male	$8,108,461	$13,440,910	$17,091,889	Genital warts-male	$8,108,461
CIN1	$841,749	$1,975,720	$2,863,853	CIN1	$841,749	$1,975,720	$2,863,853	CIN1	$841,749
CIN2/3	$4,830,393	$13,375,935	$20,551,689	CIN2/3	$4,830,393	$13,375,935	$20,551,689	CIN2/3	$4,830,393
Cervical cancer	$1,215,209	$4,347,953	$7,539,668	Cervical cancer	$1,215,209	$4,347,953	$7,539,668	Cervical cancer	$1,215,209
***Total Direct Costs***	***$23*,*667*,*766***	***$47*,*397*,*781***	***$66*,*170*,*586***	***Total Direct Costs***	***$23*,*667*,*766***	***$47*,*397*,*781***	***$66*,*170*,*586***	***Total Direct Costs***	***$23*,*667*,*766***

In the 50%F/40%M vaccination scenario, the estimated total direct cost avoided would be nearly $38 million in 25 years and $71.4 million in 50 years. The estimated costs avoided for genital warts in females goes from almost $13 million in 25 years to $20.6 million in 50 years; whereas in males it would be $16.2 million in 25 years and $26.6 million in 50 years. For CIN1, the estimated costs avoided in this scenario would be $1.2 million in 25 years and $2.6 million in 50 years. The estimated costs avoided for CIN2/3 would be $6 million in 25 years and $16.3 million in 50 years. For cervical cancer we see an estimated cost decrease of $1.5 million in the first 25 years and $5.3 million in 50 years.

Finally, under a broader vaccination scenario of 80%F/64%M, the estimated total direct costs avoided would be nearly $90 million in 25 years and $162 million in 50 years. The estimated cost avoided for genital warts in females goes from $31.7 million in 25 years to $51.2 million in 50 years; whereas in males it goes from $38 million in 25 years to $62.7 million in 50 years. The CIN1 estimated cost reduction under this vaccination scenario is $2.7 million in 25 years and $5.8 million in 50 years. The CIN2/3 analysis shows an estimated cost reduction of $13 million in 25 years and $32.3 million in 50 years. Direct costs attributable to cervical cancer would have an estimated decrease of $3.4 million in 25 years and $10.5 million in 50 years.

Table 2 presents the results of cost-effectiveness analysis of vaccination strategies. In Puerto Rico, increased vaccination uptake of females and males aged 11–15 year old with a HPV4 vaccine can reduce incidence and deaths from HPV-related conditions and related costs. For the 80%F/64%M uptake scenario, the study resulted in an ICER of $1,960 per QALY. The 80%F/64%M ICER is estimated to dominate over the alternative scenarios of no vaccination, 34%F/13%M vaccination and 50%F/40%M vaccination.

**Table 2:** Cost-effectiveness analysis of HPV vaccination scenarios.

	Discounted Total		Incremental		
Scenario	Cost/Person (USD)*	QALYs/Person (year)†	Cost/Person (USD)*	QALYs/Person (year)†	Cost/QALYs (USD/year)‡
**Puerto Rico F+M 11–15 years no-vaccination scenario**	**703.53**	**26.805 54**	**-**	**-**	**-**
Puerto Rico F+M 11–15 years vaccination scenario: 34% F/13% M	716.82	26.808 47	13.29	0.002 93	strongly dominated
Puerto Rico F+M 11–15 years vaccination scenario:50%F/40%M	728.26	26.812 71	24.73	0.007 16	strongly dominated
Puerto Rico F+M 11–15 years vaccination scenario:80%F/60%M	736.01	26.822 12	32.49	0.016 57	1 960

F = Female; M = Male

*Cost rounded to 0.01.

^**†**^ QALYs rounded to 0.00001.

^‡^ Cost/QALY rounded to 1.

## Discussion

Consistent with previously conducted HPV vaccination health economic analyses, our data suggests that an HPV4 vaccination program can result in important public health and economic benefits in reducing HPV-related diseases over screening only, in the absence of vaccination. Additionally, increasing the vaccination coverage in females and males would greatly decrease HPV-related morbidity and mortality, resulting in greater HPV-related disease total direct cost avoidance in Puerto Rico. Although increasing vaccination rates would be expected to result in better outcomes, the model adaptation provides the means to estimate the magnitude of disease avoidance across the different scenarios within the modelled timeframe, hence providing additional information for the need to increase vaccination rates in a timely fashion.

As the coverage rate increases, faster reductions in HPV-related disease incidences and costs are observed. At the 80%F/64%M vaccination coverage, the model shows that the vaccine program is cost-effective under the established threshold of $50,000 per QALY. The ICER of $1,960 per QALY is also consistent with the WHO [39] threshold that is three times less than the GDP per capita [38], which for Puerto Rico was estimated at $28,325 in 2013 [40].

Given that cervical cancers have a more prolonged natural history, the greatest reductions in disease incidence within the first 25 years of the introduction of HPV4 are estimated to derive from the prevention of HPV 6/11- related genital warts. Real-world evidence of the impact of gender-neutral HPV vaccination has shown that, although the focus in some countries has been towards cervical cancer prevention among females, in others, the burden of HPV-associated cancers in comparable in males and females. All men have a high burden of HPV-associated diseases, a problem that is increased in industrialized countries but that could be diminished if they were immunized as well [27].

Australia’s post-licensure monitor studies showed the first signs of a quadrivalent vaccine impact by reporting a sharp decrease in additional diagnoses of genital warts in young females within two years after vaccine, and a smaller decrease in new genital warts cases in young heterosexual men, suggesting indirect protection through herd immunity. Hariri has reported that emerging data from countries with high vaccination coverages, such as Denmark and Sweden, and those with lower coverages, such as the United States, Germany, and New Zealand, further strengthen the evidence of direct and indirect impacts of the HPV4 vaccine [6]. A systematic literature review done by Mariani, et al. confirms this finding [41]. As mentioned earlier, in Puerto Rico, increasing vaccination coverage from 34%F/13%M to 80%F/64%M can potentially decrease direct costs in 100 years by nearly 50%. Fifty-six percent of the reduction in direct costs would be attributed to the decline of the incidence of genital warts.

Among study limitations is the exclusion of health benefits and cost offsets associated with other HPV related-diseases which may be a consequence of HPV 6, 11, 16 and 18 infections, whose inclusion may show an improvement on HPV4 cost-effectiveness. Also, only direct medical costs were included in the analysis, HPV associated labor productivity loss was not included, which represented for the US an estimate of $1 billion in cervical cancer [42].

In Puerto Rico, labor productivity loss due to cancer deaths in 2004 was estimated to be around $64 million [43]. Even when the inclusion within the cost/QALY estimations of labor productivity loss is not endorsed by the US Panel on cost-effectiveness in Health and Medicine [44], it can provide a better understanding of the economic impact of health interventions.

Another limitation of the study is that data on the age-specific incidences of genital warts are under-reported in Puerto Rico, leading to inaccuracies in the model estimations. We therefore calibrated the model based on the annual age-specific incidences of genital warts in the US. In addition, data pertaining to the costs of follow-up care for incident episodes of HPV-related disease, including genital warts, were not publicly available. Therefore, these costs were estimated based on claims analysis from a private health insurance company (using ICD 9 and CPT codes). More accurate estimations of these costs represent an area for future research.

## Conclusions and implications for health policy

HPV vaccination is covered by health insurance plans for females and males in Puerto Rico; however vaccination uptake is still suboptimal. Results show that increasing the vaccination coverage can lead to a reduction in the incidence and costs of HPV-related morbidity in Puerto Rico. Although these effects will not be seen immediately for cancer due to its natural history, the impact on anogenital warts would be observed faster. Notwithstanding, it is important to foster direct attention to the importance of the vaccination and in order to be fully protected, completing the vaccination schedule. As stated before, in Puerto Rico local legislation requires insurance companies to cover the vaccine but the vaccination itself is not mandatory according to the local vaccination list. Public health advocates and decision-makers can use the results of this model adaptation to develop strategies to increase vaccination rates in order to potentially avoid additional healthcare expenditures associated with HPV-related disease. Furthermore, this analysis should be used as a basis for future, more in depth analyses that further explore the burden of HPV-related diseases and the positive impacts that vaccination programs can have on them. That way the government can adopt a properly informed position regarding HPV vaccination. Our study supports putting in effect HPV4 vaccination for the prevention of HPV-related pre-cancerous lesions, cancers and genital warts in Puerto Rico. Continued surveillance of HPV-related conditions will help evaluate the long-term impact of vaccination and guide public health efforts.

## Supporting information

S1 Fig Web-model inputs. (XLSX)

S2 Fig Modeled health-economics results. (PPTX)
