# Correction: Modelling the effects of quadrivalent Human Papillomavirus (HPV) vaccination in Puerto Rico

**DOI:** 10.1371/journal.pone.0204759

**Published:** 2018-09-21

**Authors:** Ana Patricia Ortiz, Karen J. Ortiz-Ortiz, Moraima Ríos, José Laborde, Amit Kulkarni, Matthew Pillsbury, Andreas Lauschke, Homero A. Monsanto, Cecile Marques-Goyco

There is an error in the third sentence of the second paragraph of the Introduction. The correct sentence is: “In Puerto Rico, cancer was the second leading cause of death by 2010 [8], and cervical cancer accounts for 3.9% of all cancer cases [9].”

There are errors in Table 1. The numbers from the 34%/13% scenario are repeated for the 50%/40% and 80%/64% scenarios. In addition, I the 80%/64% scenario, the years have an inverted order. Please see the corrected Table 1 here.

**Table 1:** Estimated cumulative incidence costs avoided since HPV4 vaccination program started, by vaccination scenario.

HPV Disease	Vaccination Scenario (34% Female/13% Male)			Vaccination Scenario (50% Female/40% Male)			Vaccination Scenario (80% Female/64% Male)		
	25 years	50 years	100 years	25 years	50 years	100 years	25 years	50 years	100 years
Genital warts-female	$8,671,954	$14,257,263	$18,123,487	$12,814,209	$20,640,587	$26,038,578	$31,678,147	$51,246,695	$64,843,815
Genital warts-male	$8,108,461	$13,440,910	$17,091,889	$16,223,314	$26,604,903	$33,770,465	$37,975,475	$62,669,838	$79,764,797
CIN1	$841,749	$1,975,720	$2,863,853	$1,151,140	$2,573,883	$3,687,742	$2,771,264	$5,765,318	$8,071,479
CIN2/3	$4,830,393	$13,375,935	$20,551,689	$6,053,998	$16,289,155	$24,930,480	$13,071,331	$32,275,160	$48,333,370
Cervical cancer	$1,215,209	$4,347,953	$7,539,668	$1,522,658	$5,267,437	$9,079,015	$3,365,007	$10,455,572	$17,408,785
***Total Direct Costs***	***$23*,*667*,*766***	***$47*,*397*,*781***	***$66*,*170*,*586***	***$37*,*765*,*319***	***$71*,*375*,*965***	***$97*,*506*,*280***	***$88*,*861*,*224***	***$162*,*412*,*583***	***$218*,*422*,*246***
