# Supplementary material for: Modelling the effects of quadrivalent Human Papillomavirus (HPV) vaccination in Puerto Rico
Source: PLoS One. 2017 Nov 30;12(11):e0184540. doi: 10.1371/journal.pone.0184540 (PMC5708664; doi:10.1371/journal.pone.0184540)
Supplement: S2 Fig — (PPTX) [file pone.0184540.s002.pptx]

## Slide 1
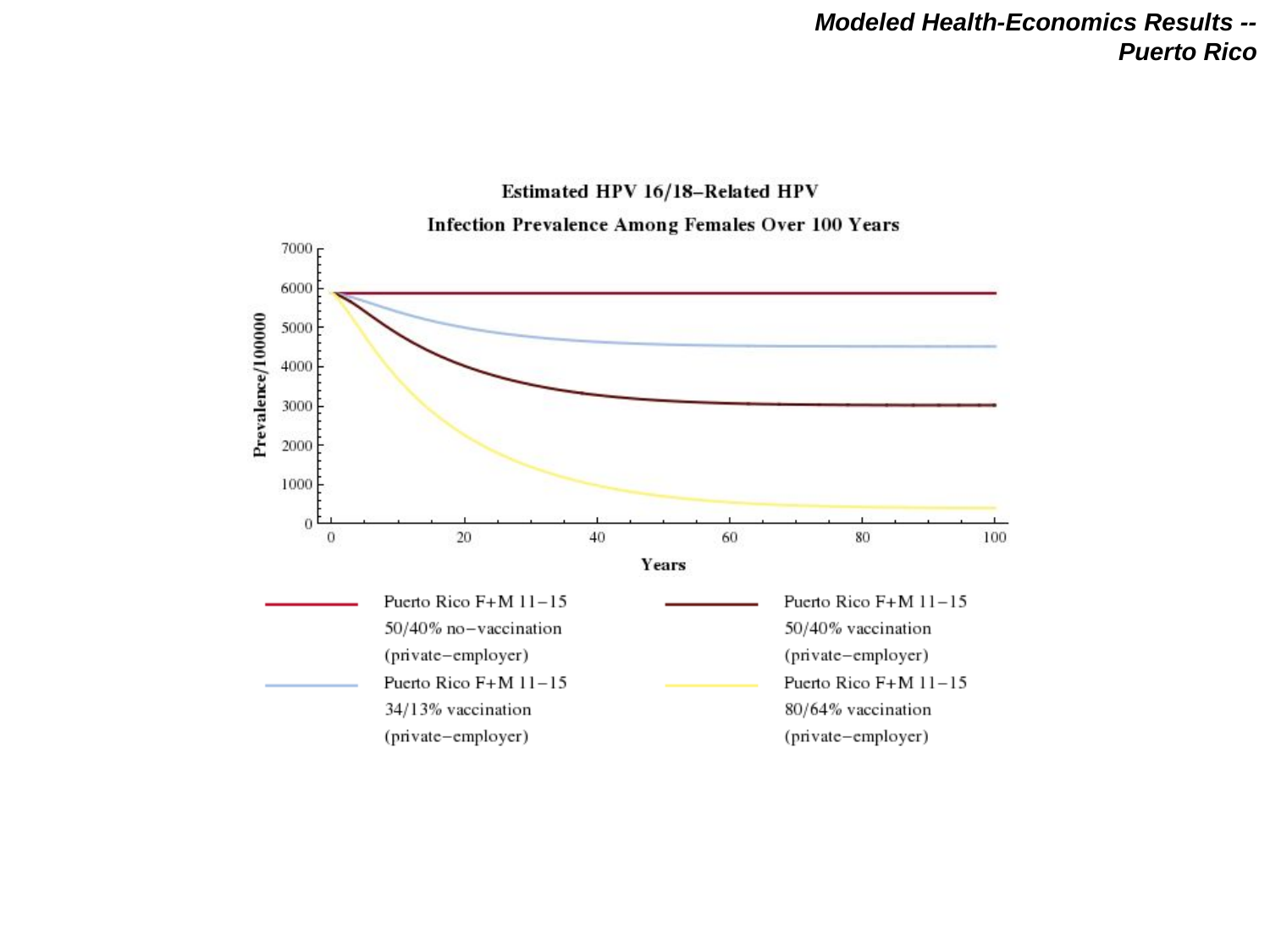

Modeled Health-Economics Results -- Puerto Rico
#

## Slide 2
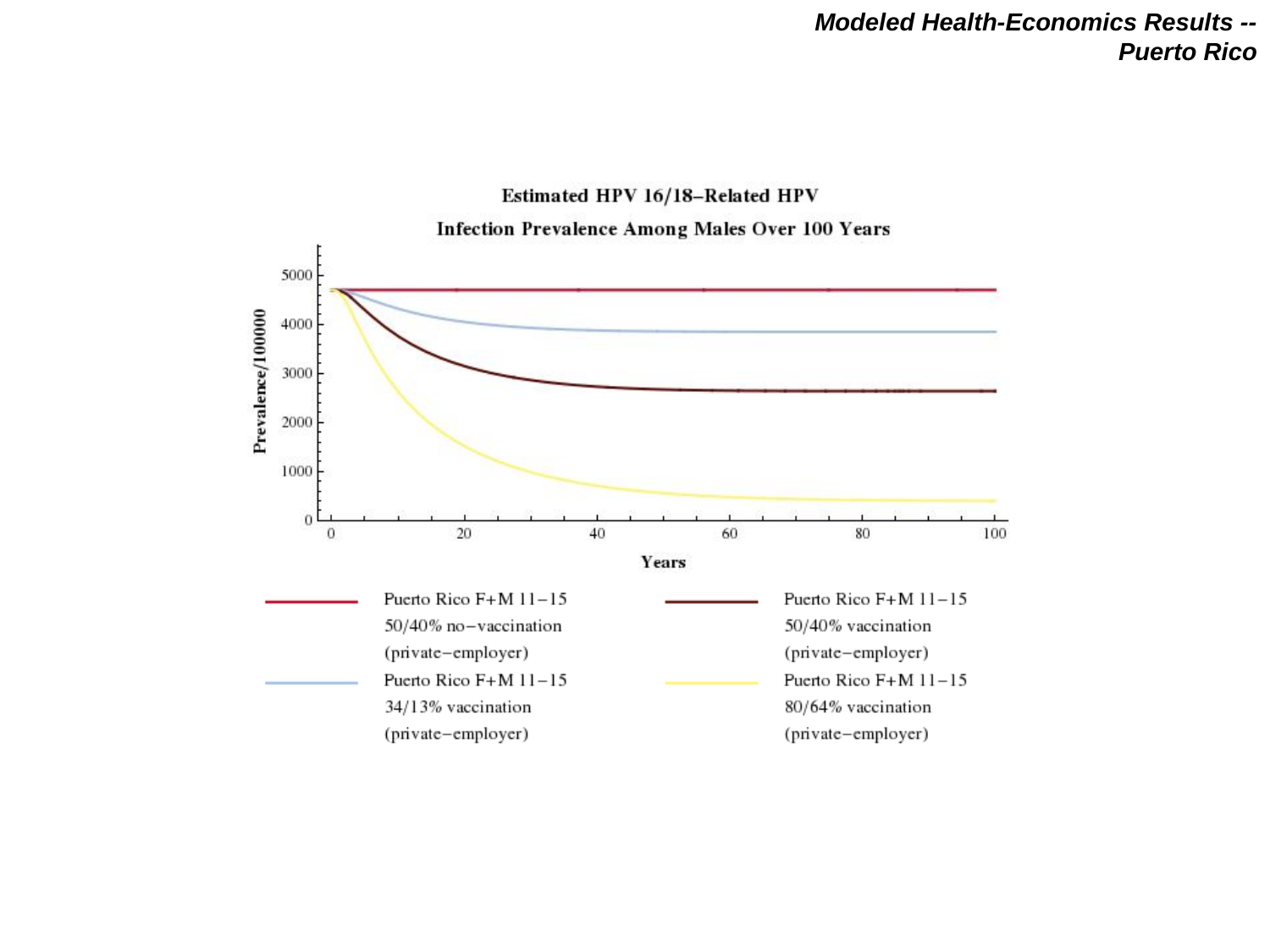

Modeled Health-Economics Results -- Puerto Rico
#

## Slide 3
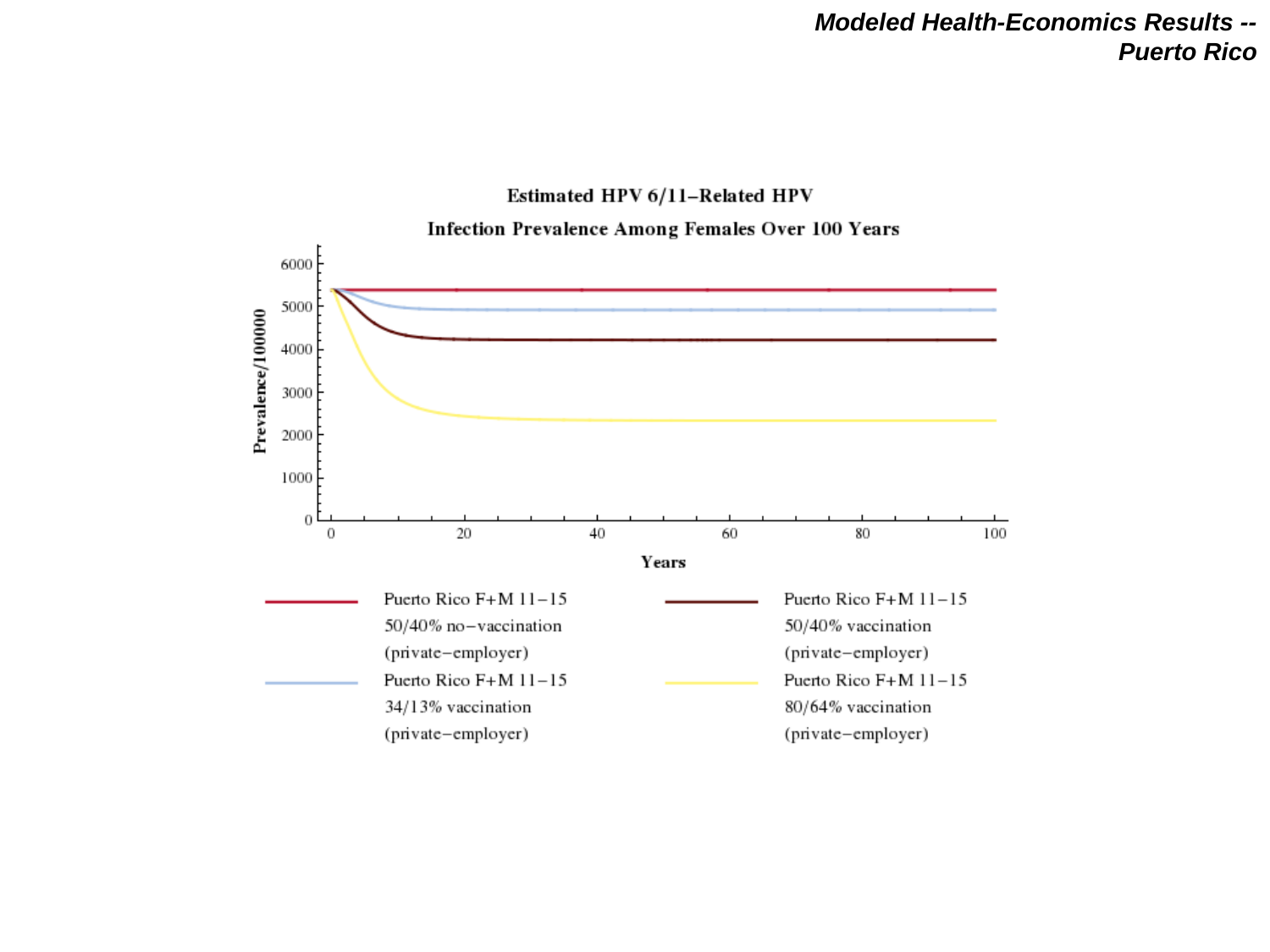

Modeled Health-Economics Results -- Puerto Rico
#

## Slide 4
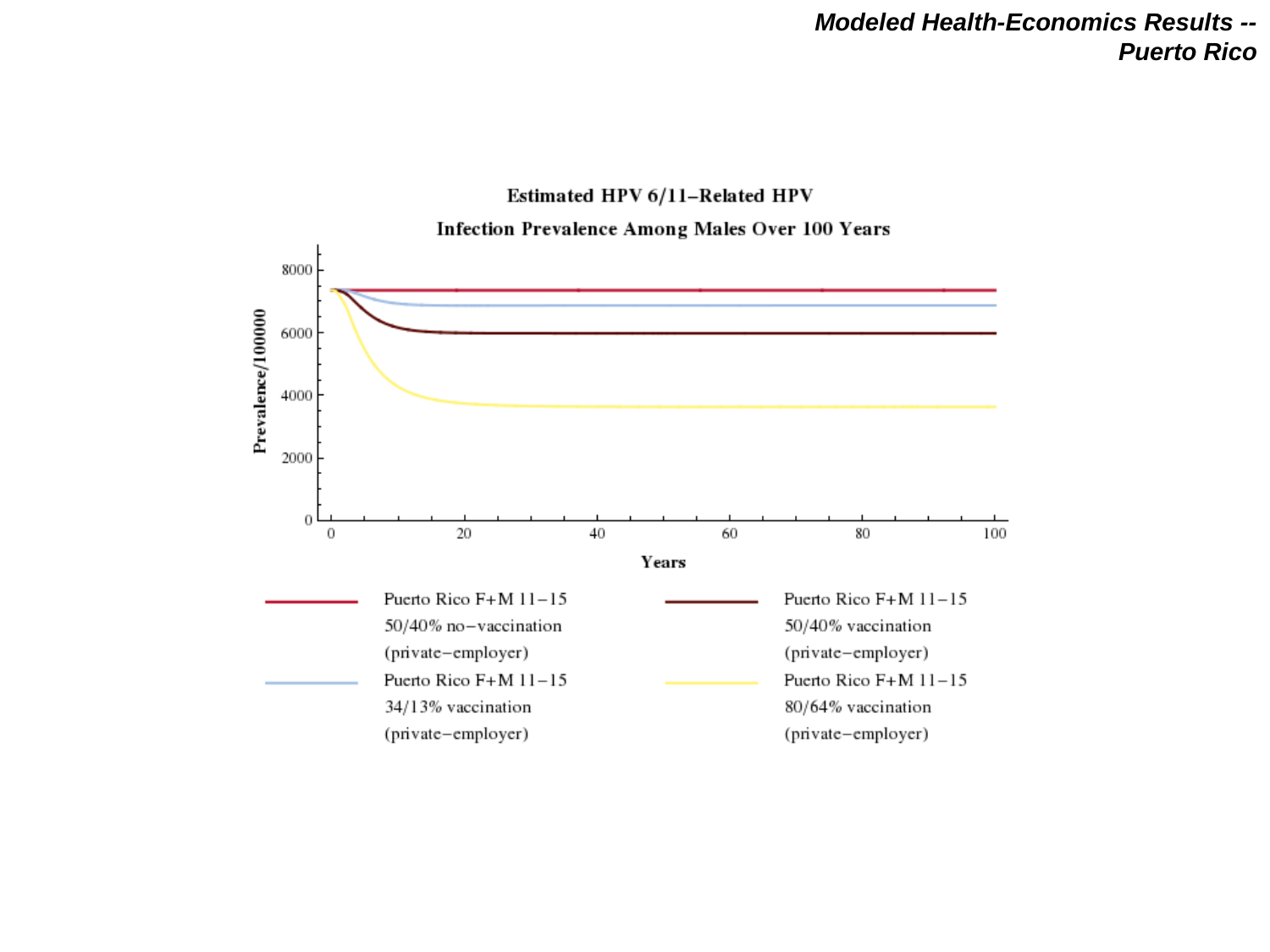

Modeled Health-Economics Results -- Puerto Rico
#

## Slide 5
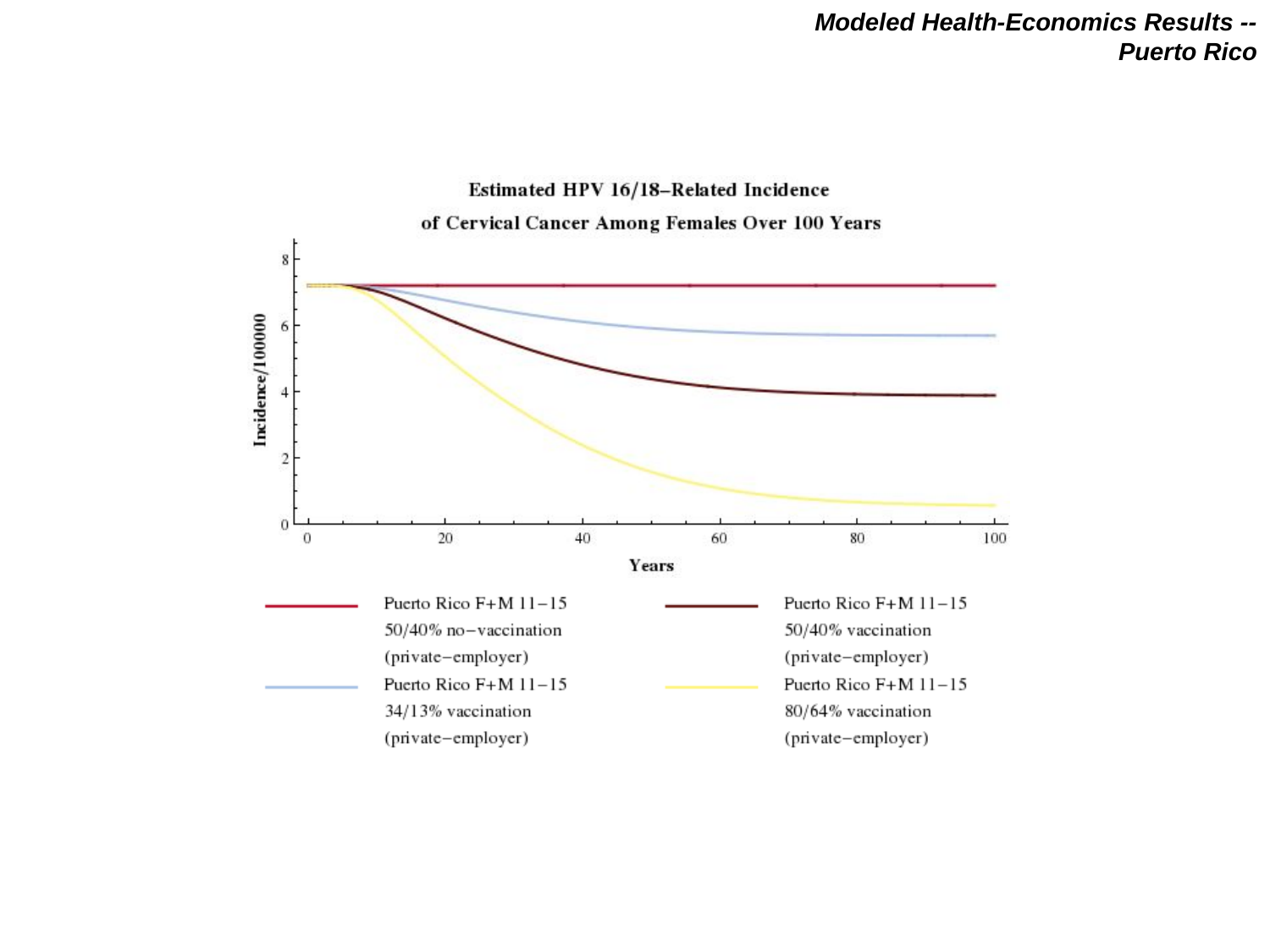

Modeled Health-Economics Results -- Puerto Rico
#

## Slide 6
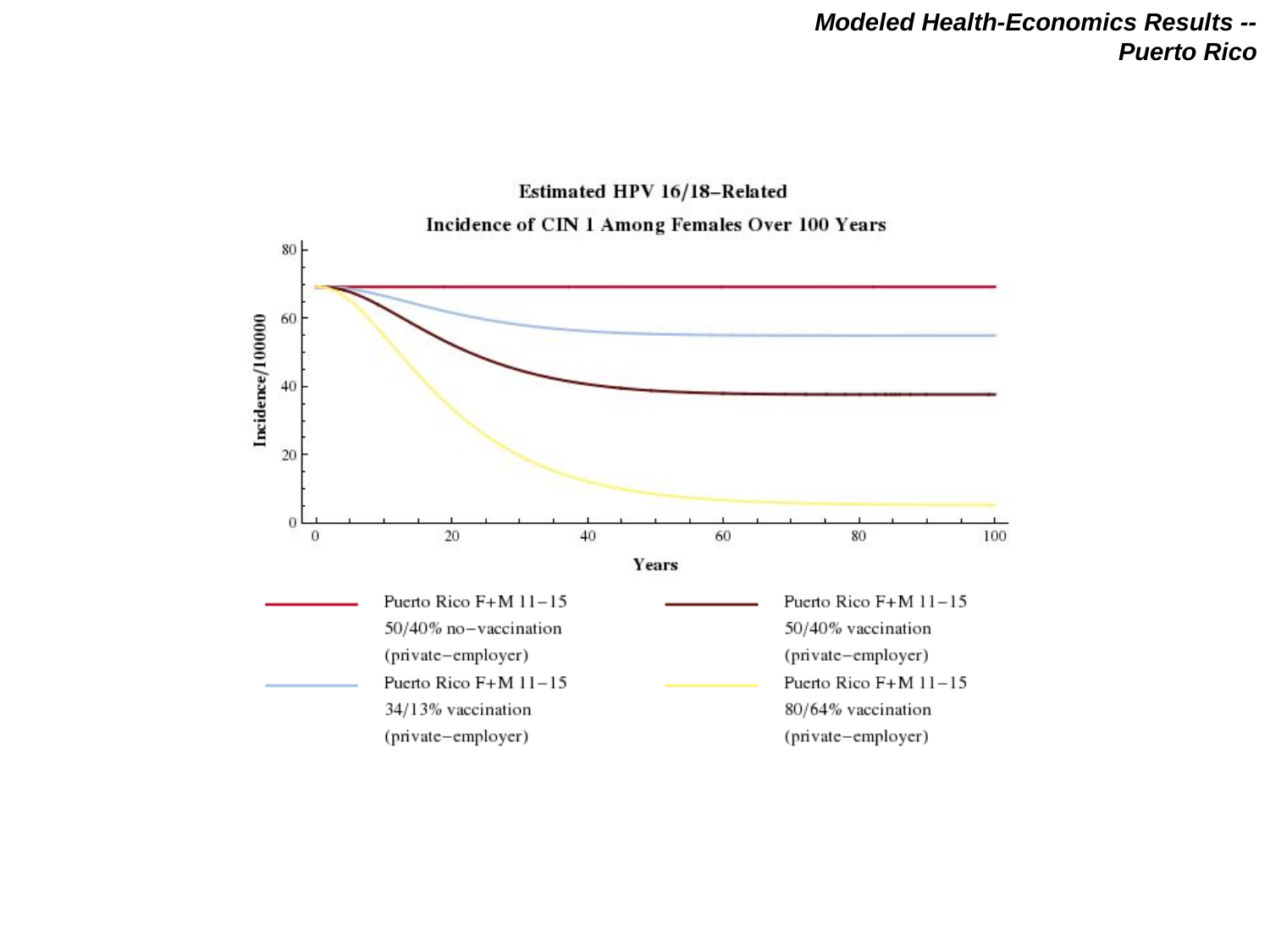

Modeled Health-Economics Results -- Puerto Rico
#

## Slide 7
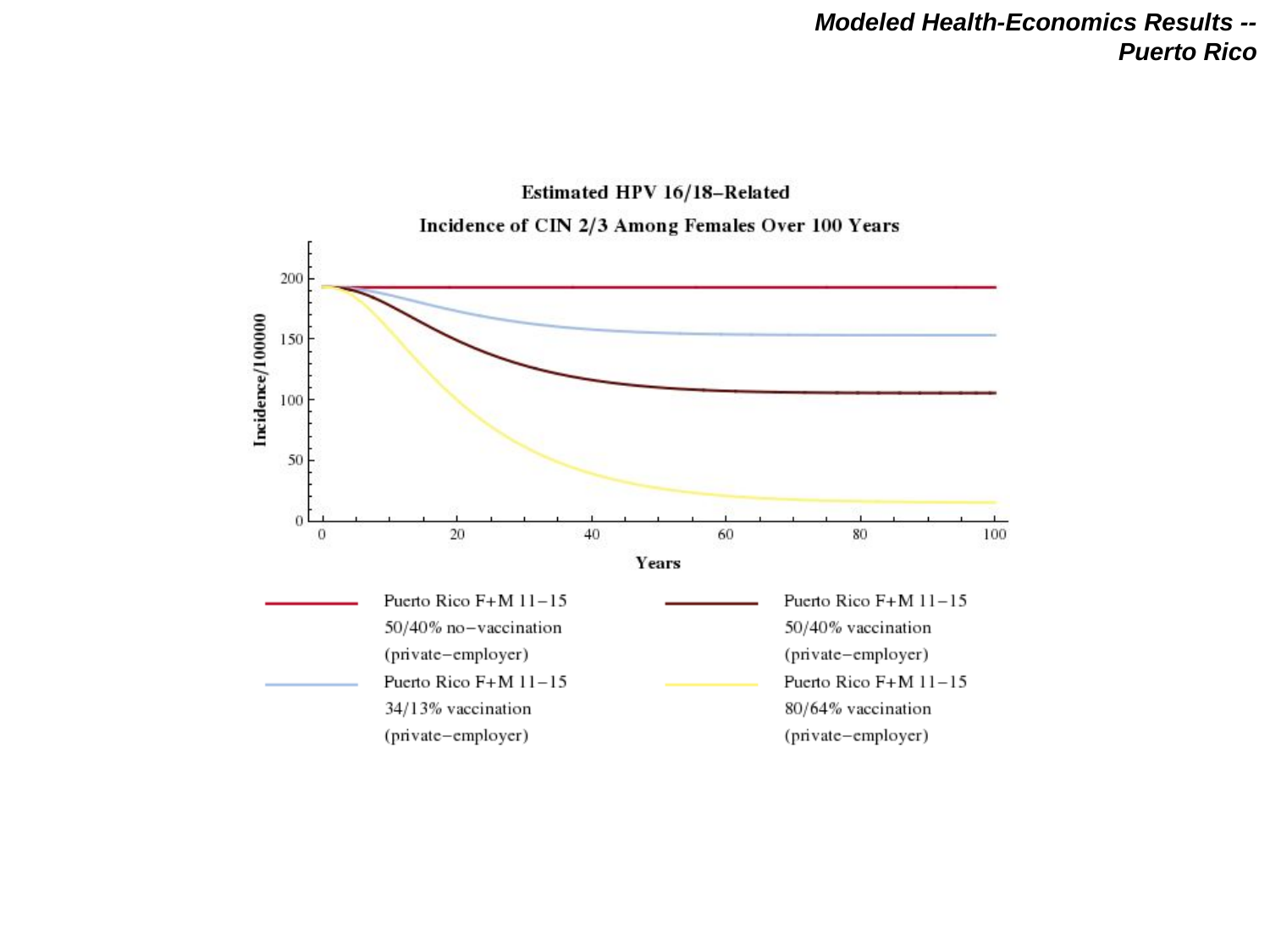

Modeled Health-Economics Results -- Puerto Rico
#

## Slide 8
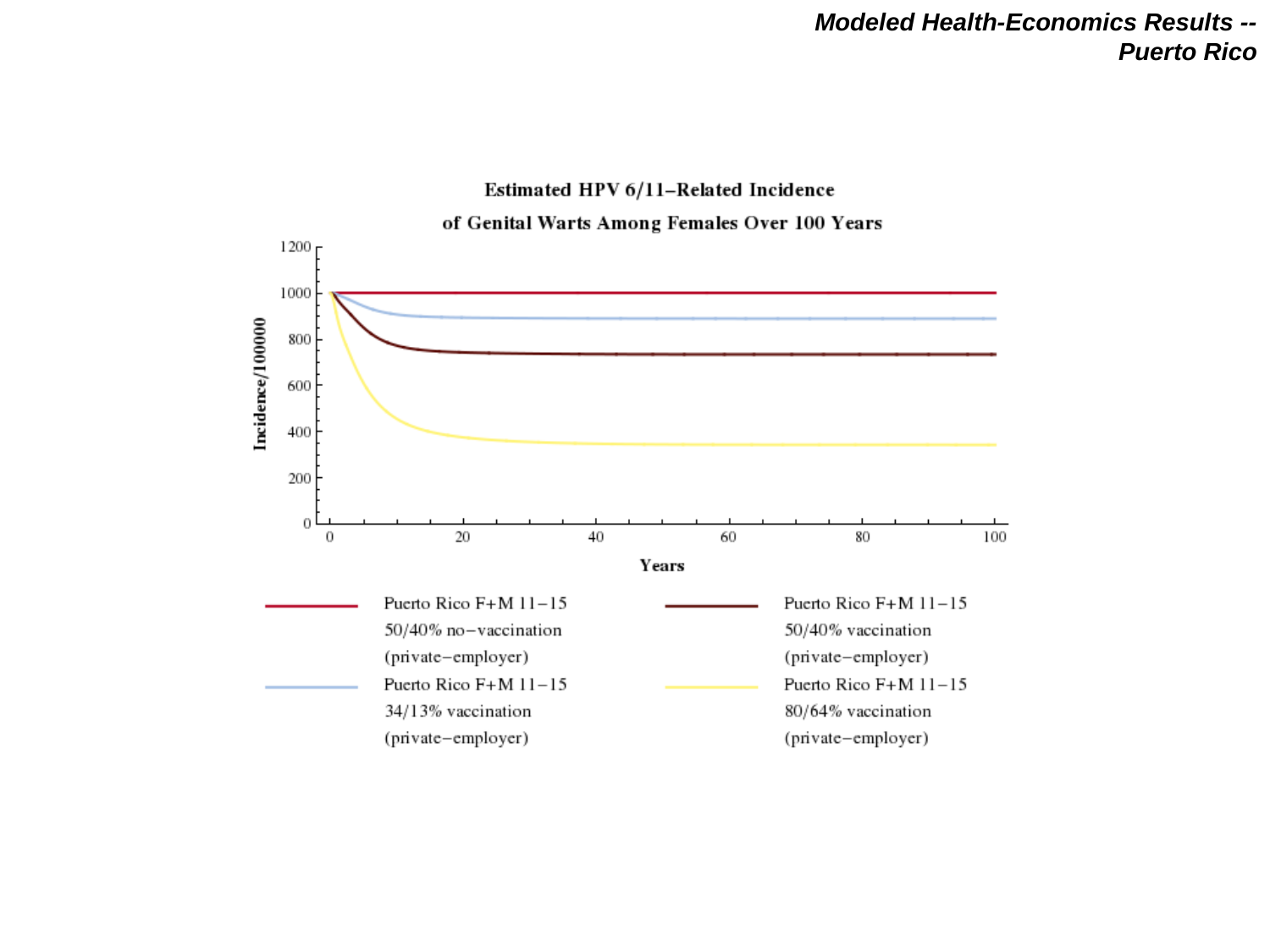

Modeled Health-Economics Results -- Puerto Rico
#

## Slide 9
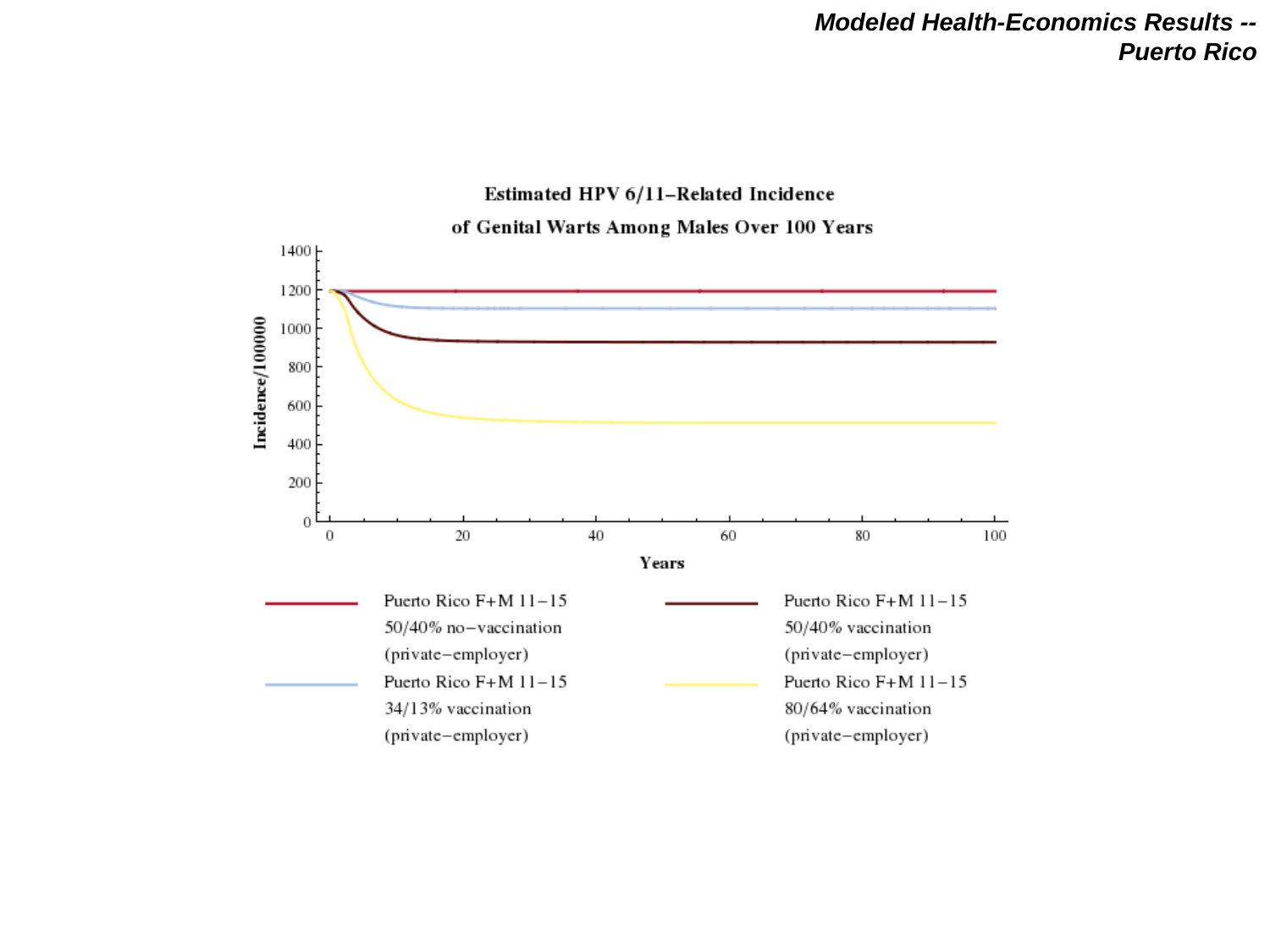

Modeled Health-Economics Results -- Puerto Rico
#

## Slide 10
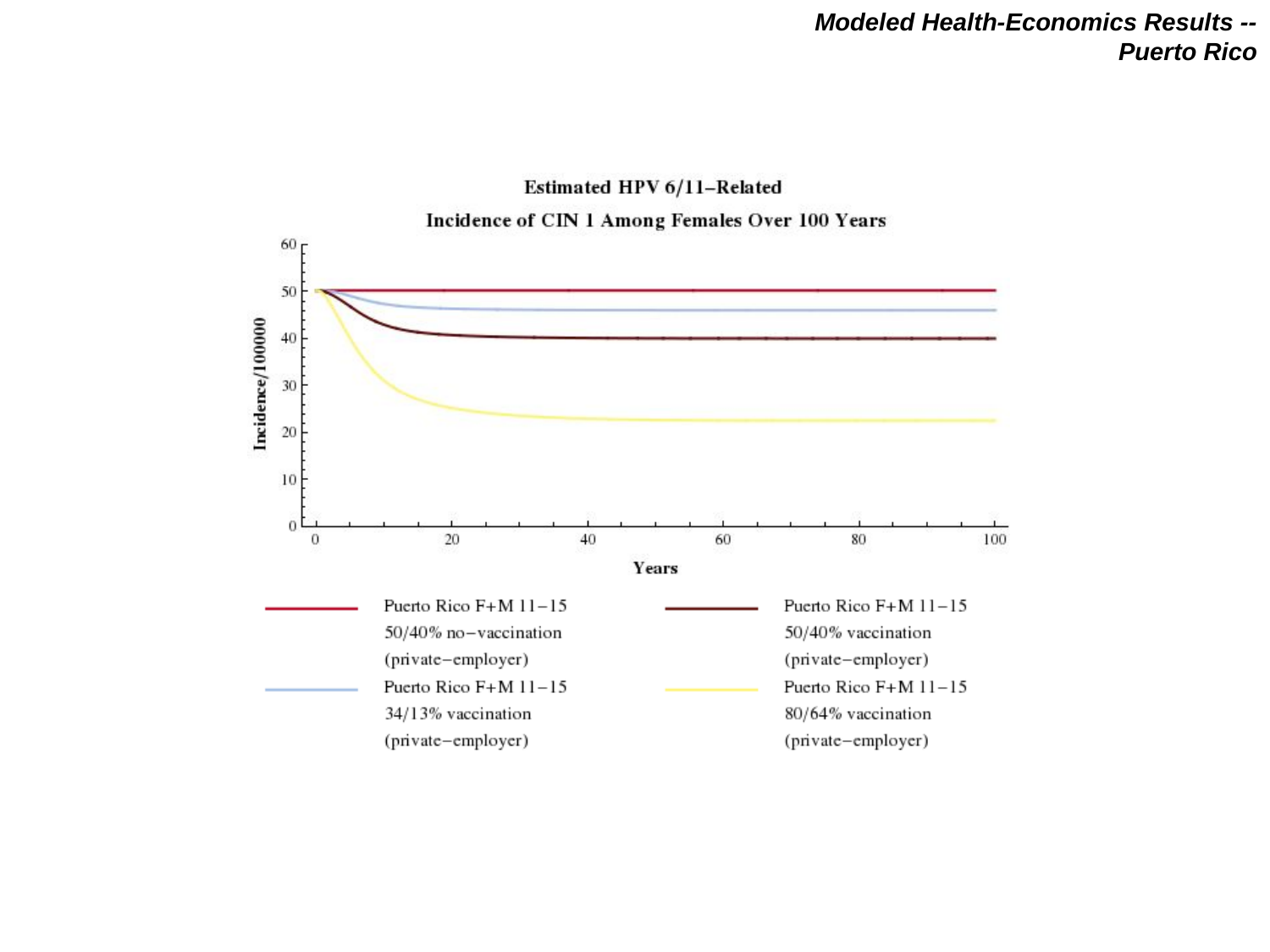

Modeled Health-Economics Results -- Puerto Rico
#

## Slide 11
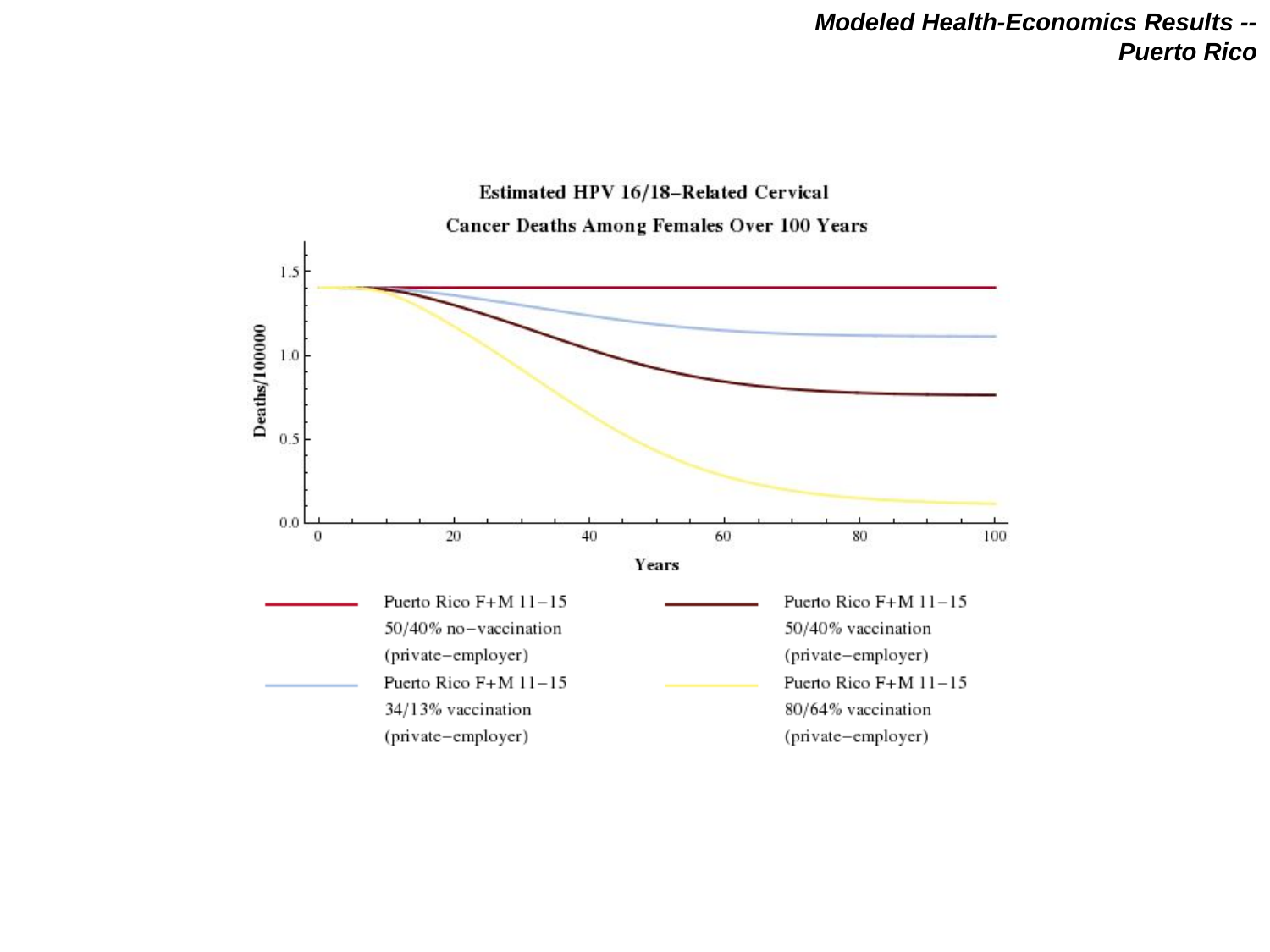

Modeled Health-Economics Results -- Puerto Rico
#

## Slide 12
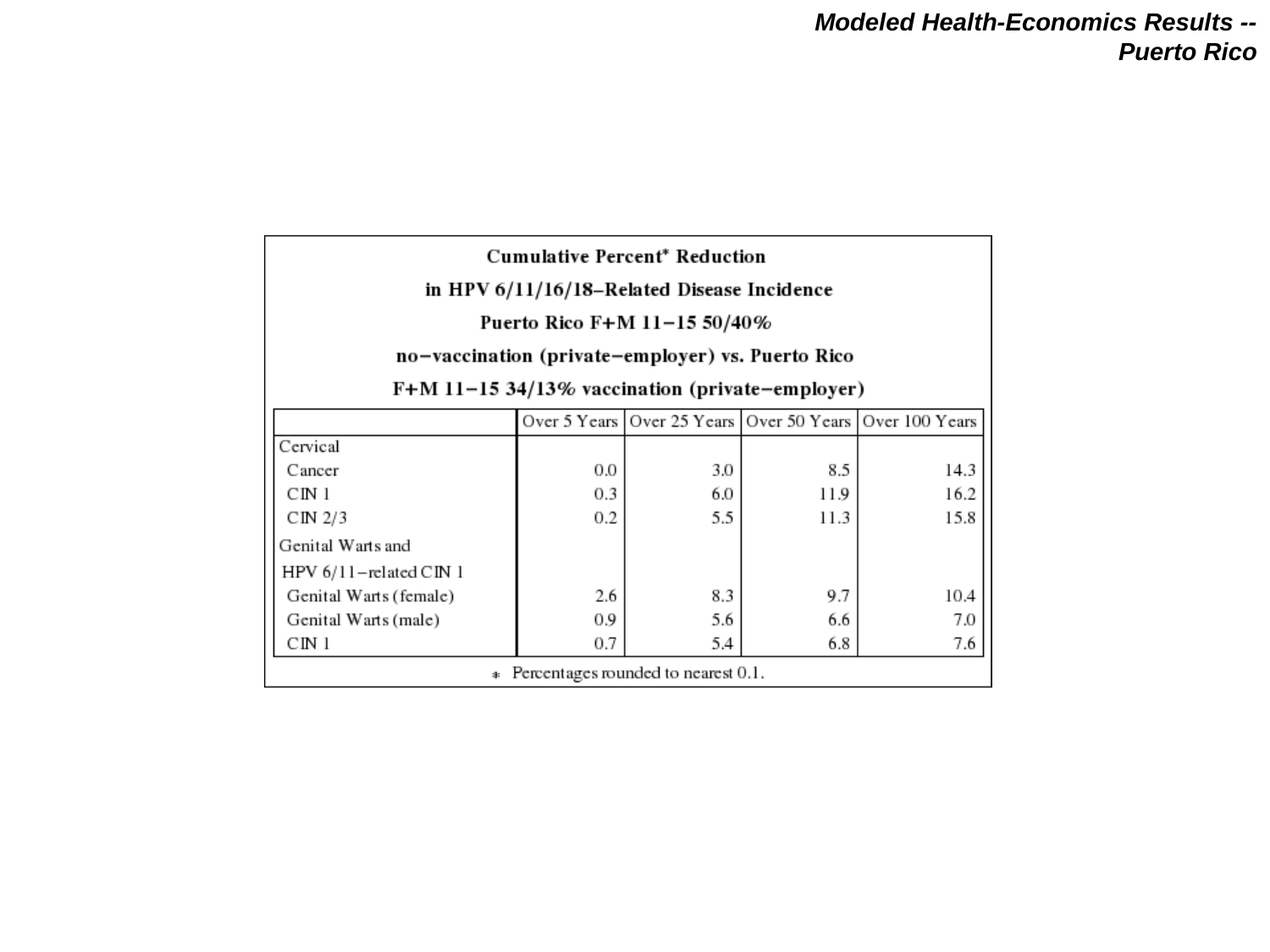

Modeled Health-Economics Results -- Puerto Rico
#

## Slide 13
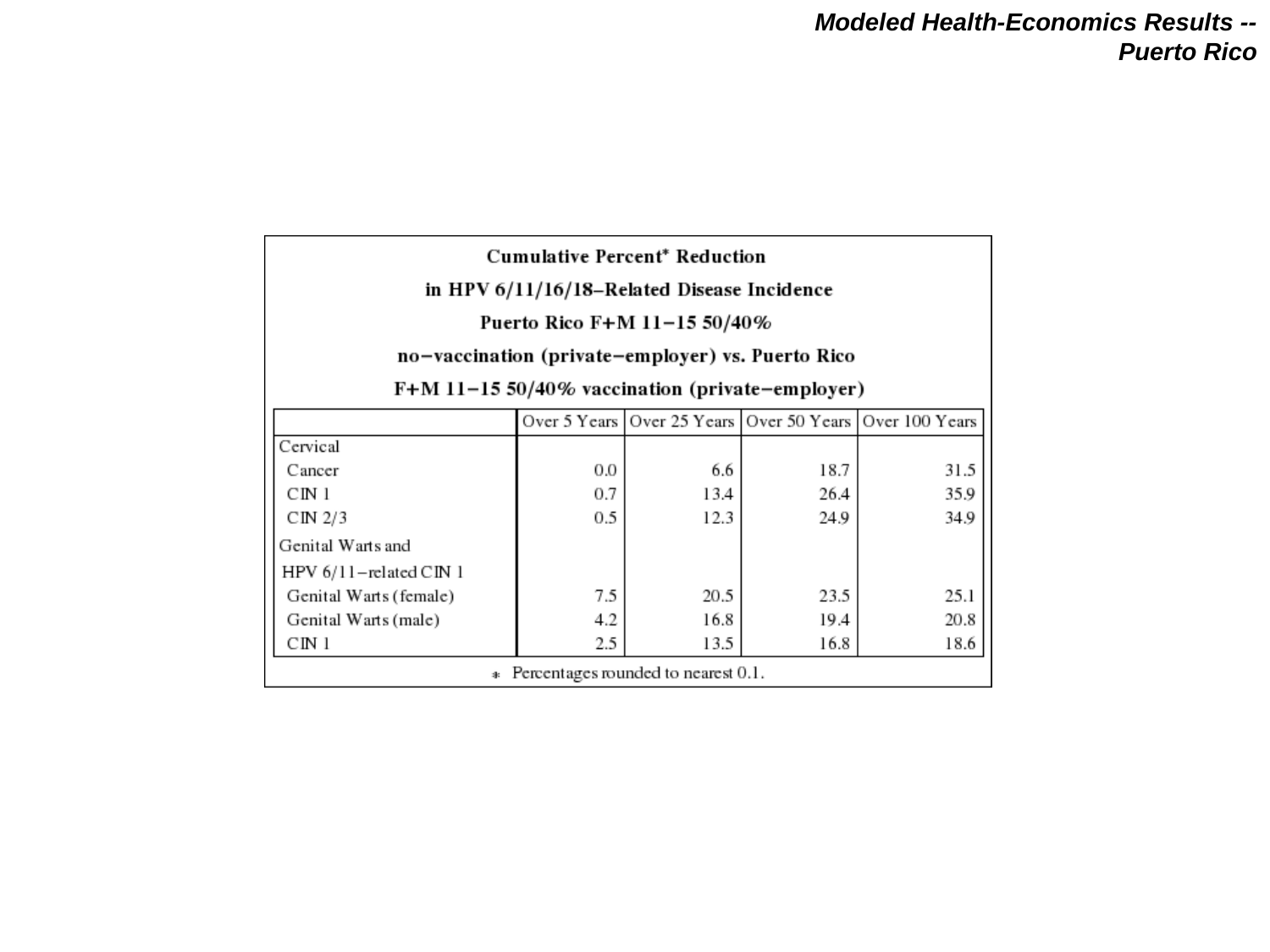

Modeled Health-Economics Results -- Puerto Rico
#

## Slide 14
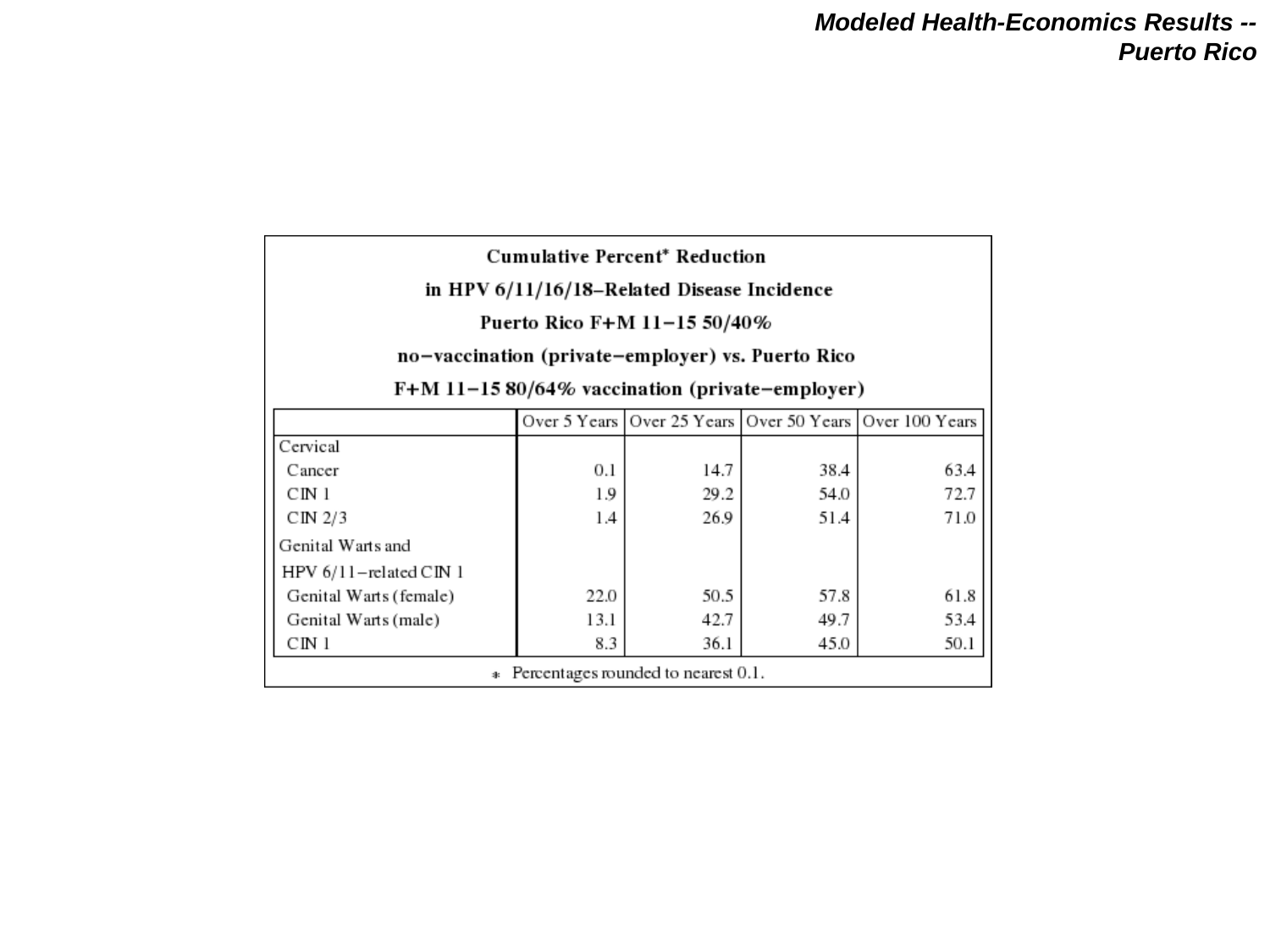

Modeled Health-Economics Results -- Puerto Rico
#

## Slide 15
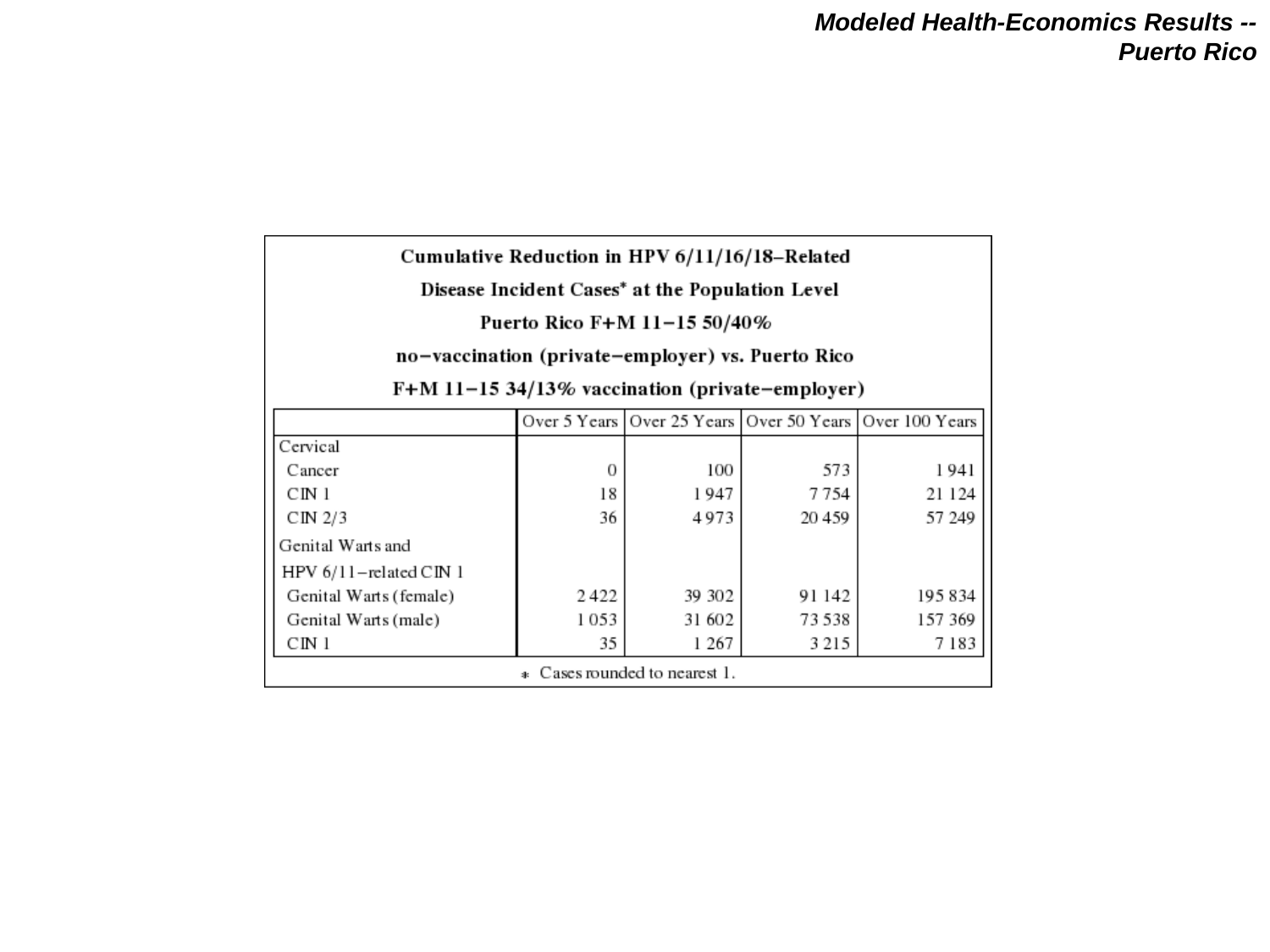

Modeled Health-Economics Results -- Puerto Rico
#

## Slide 16
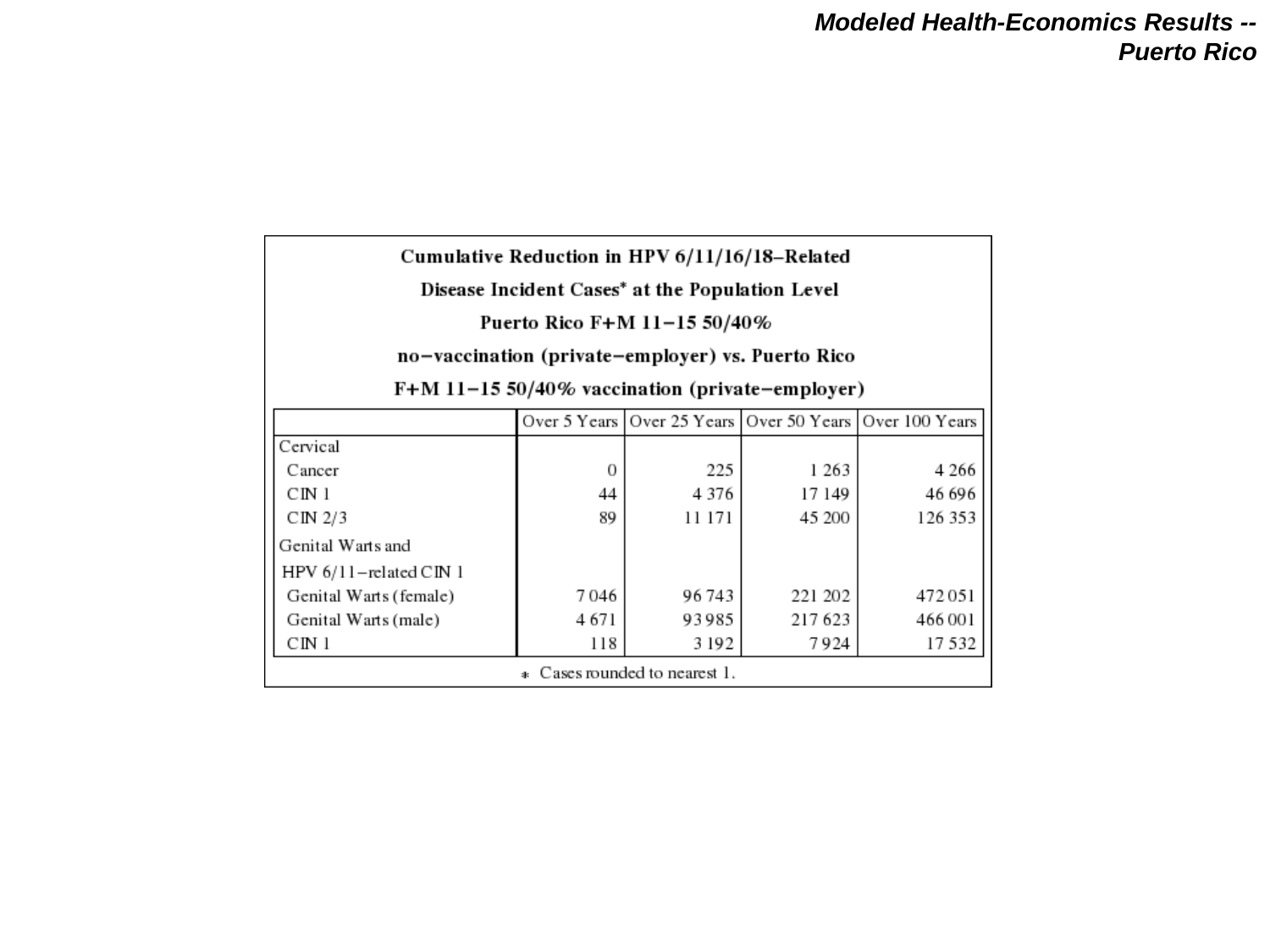

Modeled Health-Economics Results -- Puerto Rico
#

## Slide 17
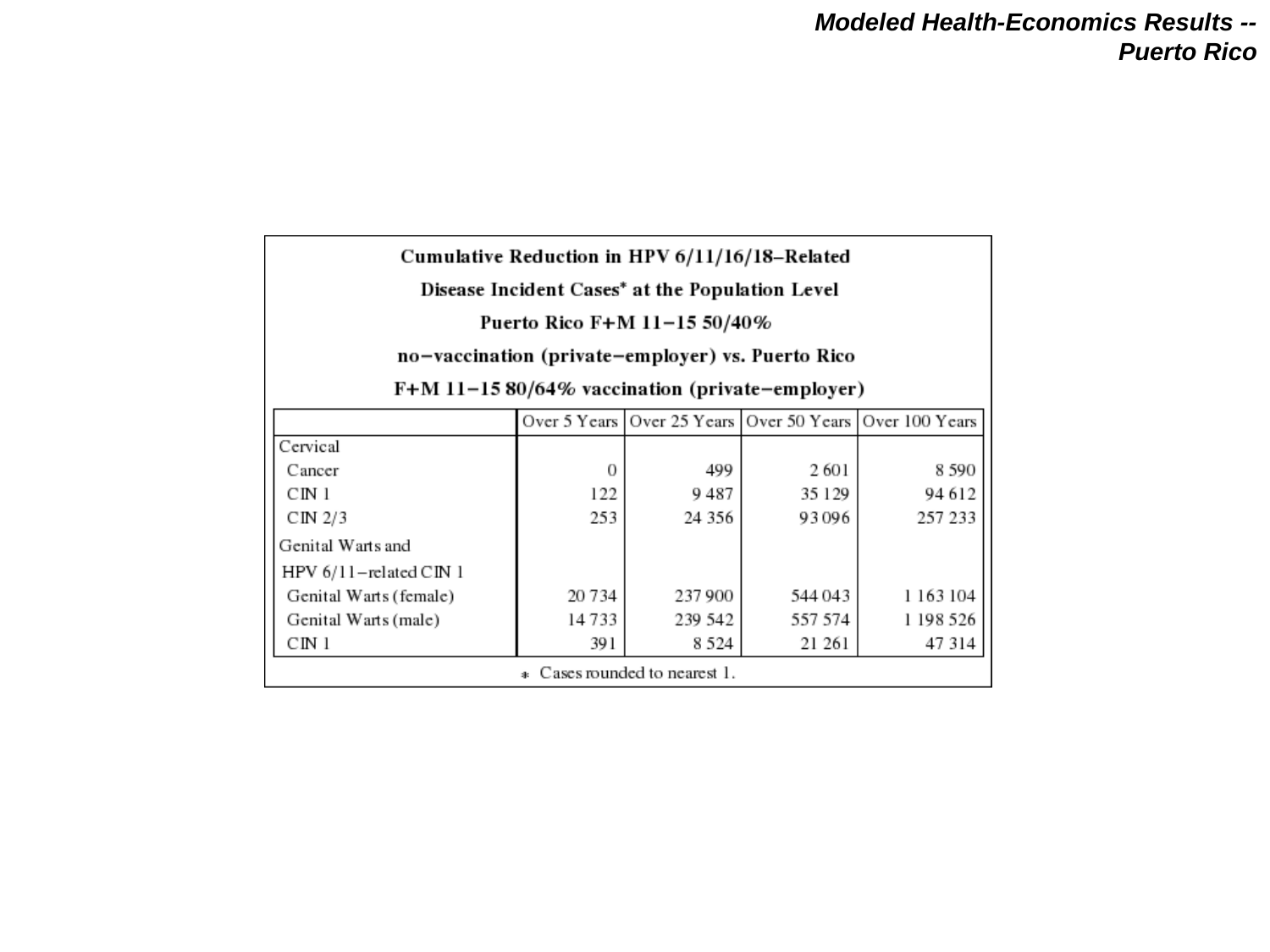

Modeled Health-Economics Results -- Puerto Rico
#

## Slide 18
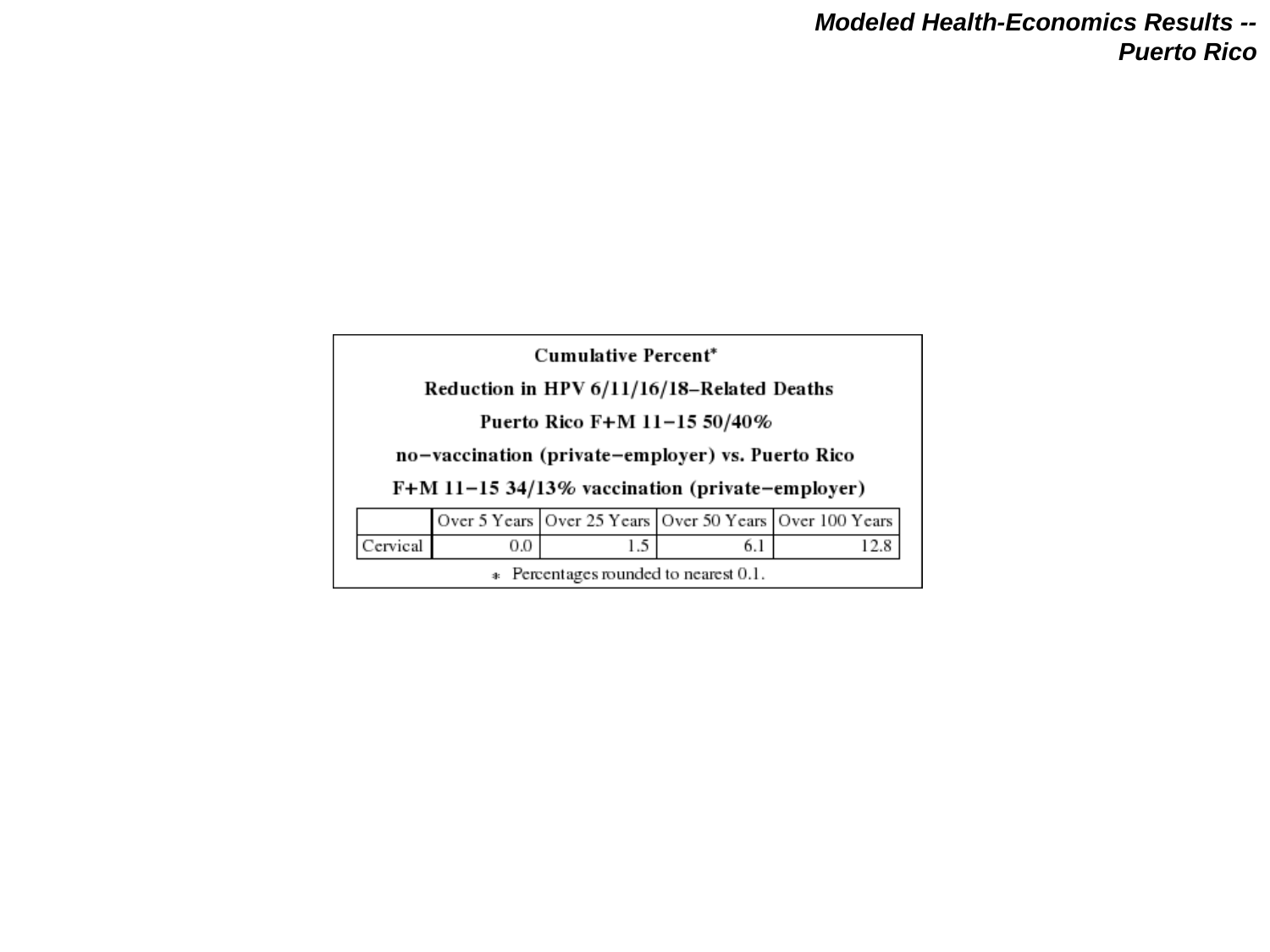

Modeled Health-Economics Results -- Puerto Rico
#

## Slide 19
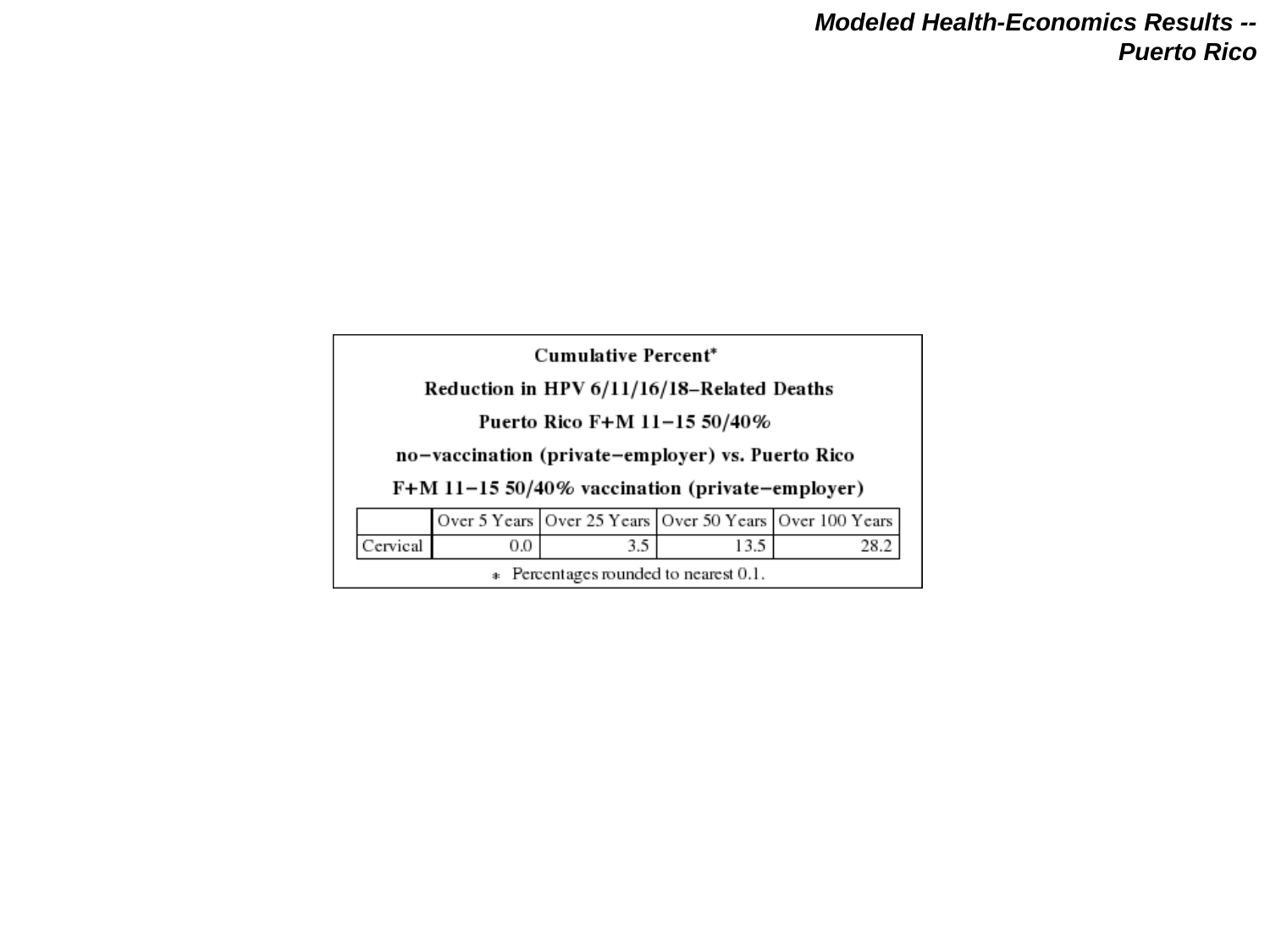

Modeled Health-Economics Results -- Puerto Rico
#

## Slide 20
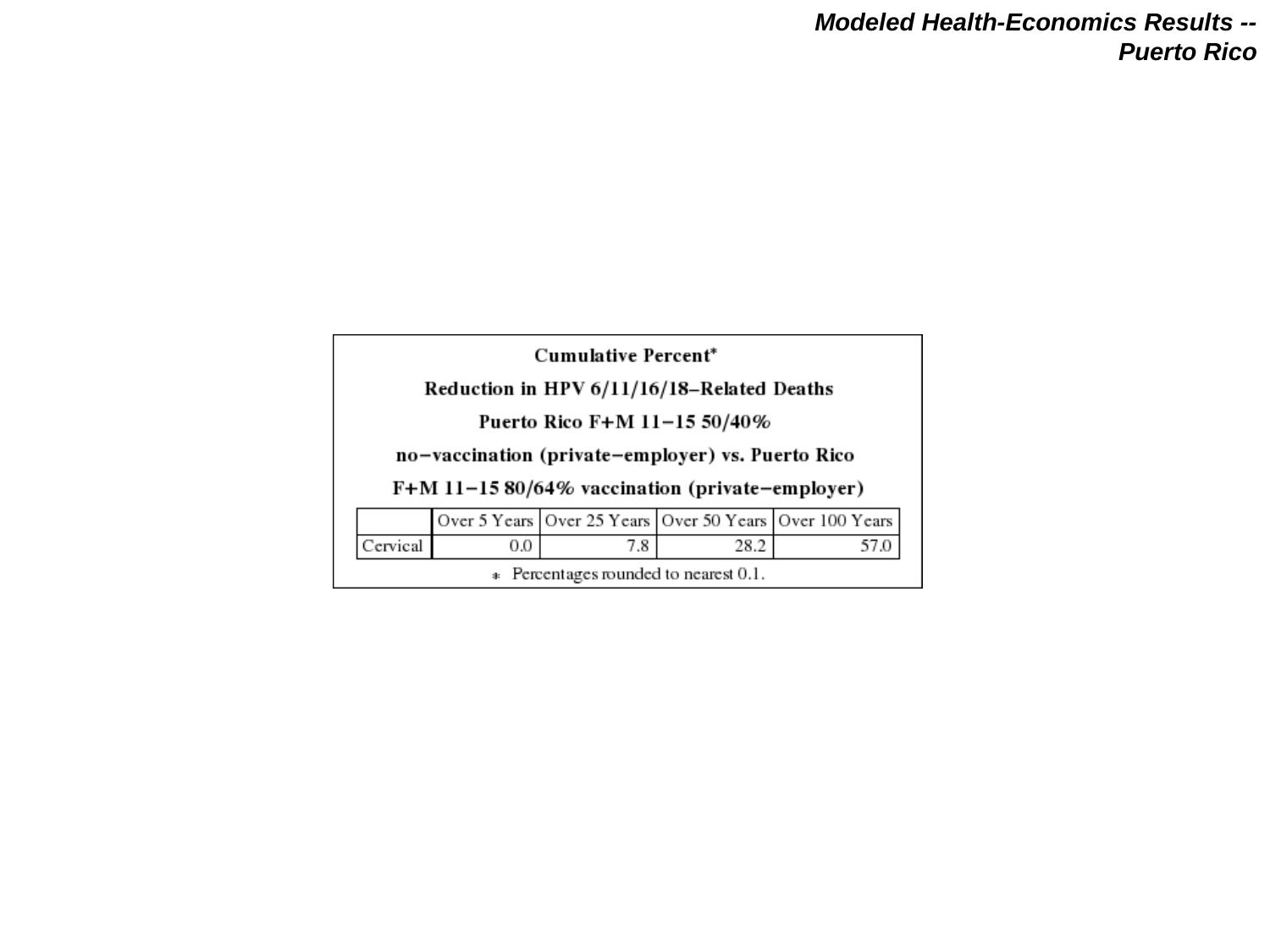

Modeled Health-Economics Results -- Puerto Rico
#

## Slide 21
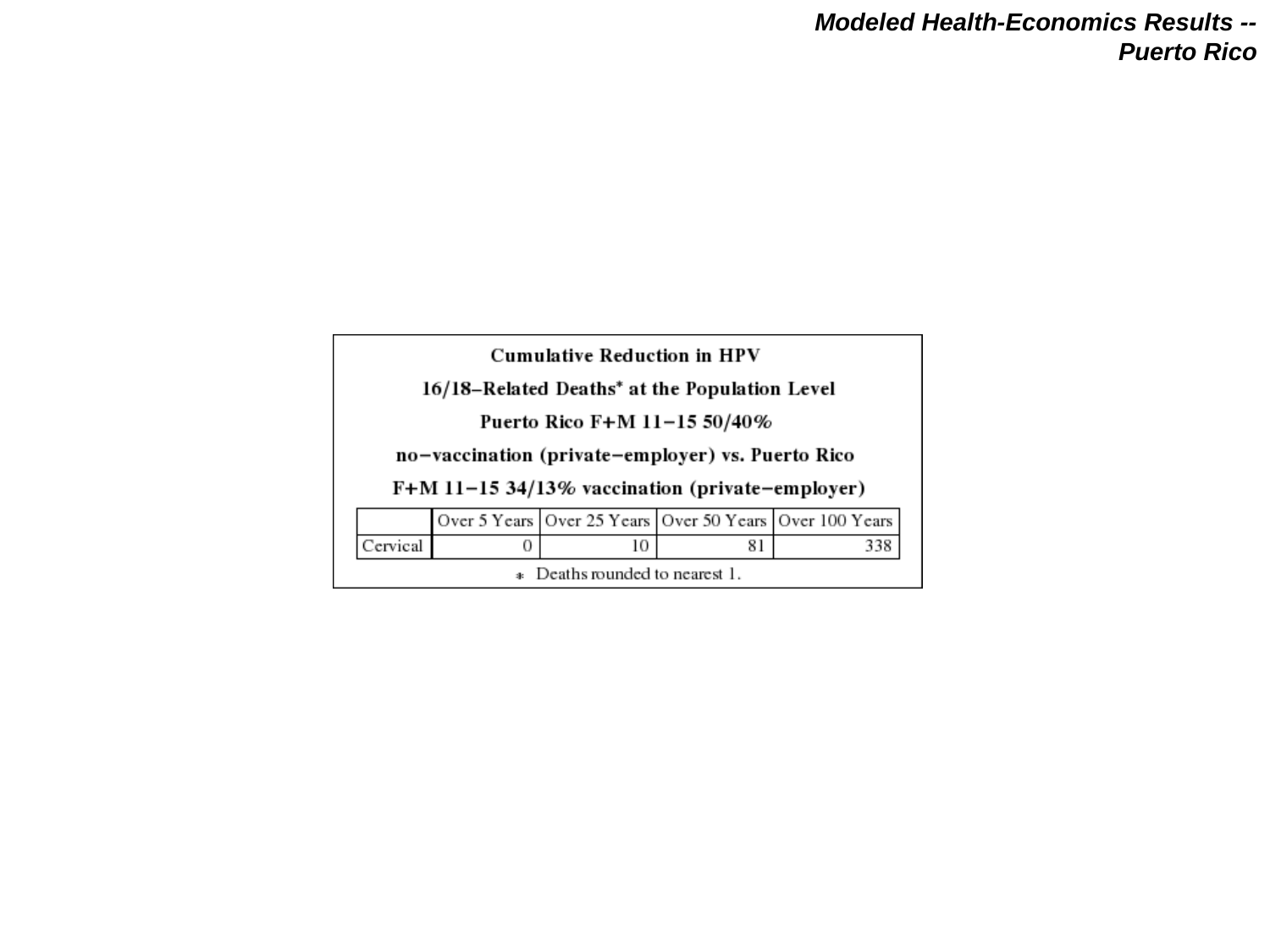

Modeled Health-Economics Results -- Puerto Rico
#

## Slide 22
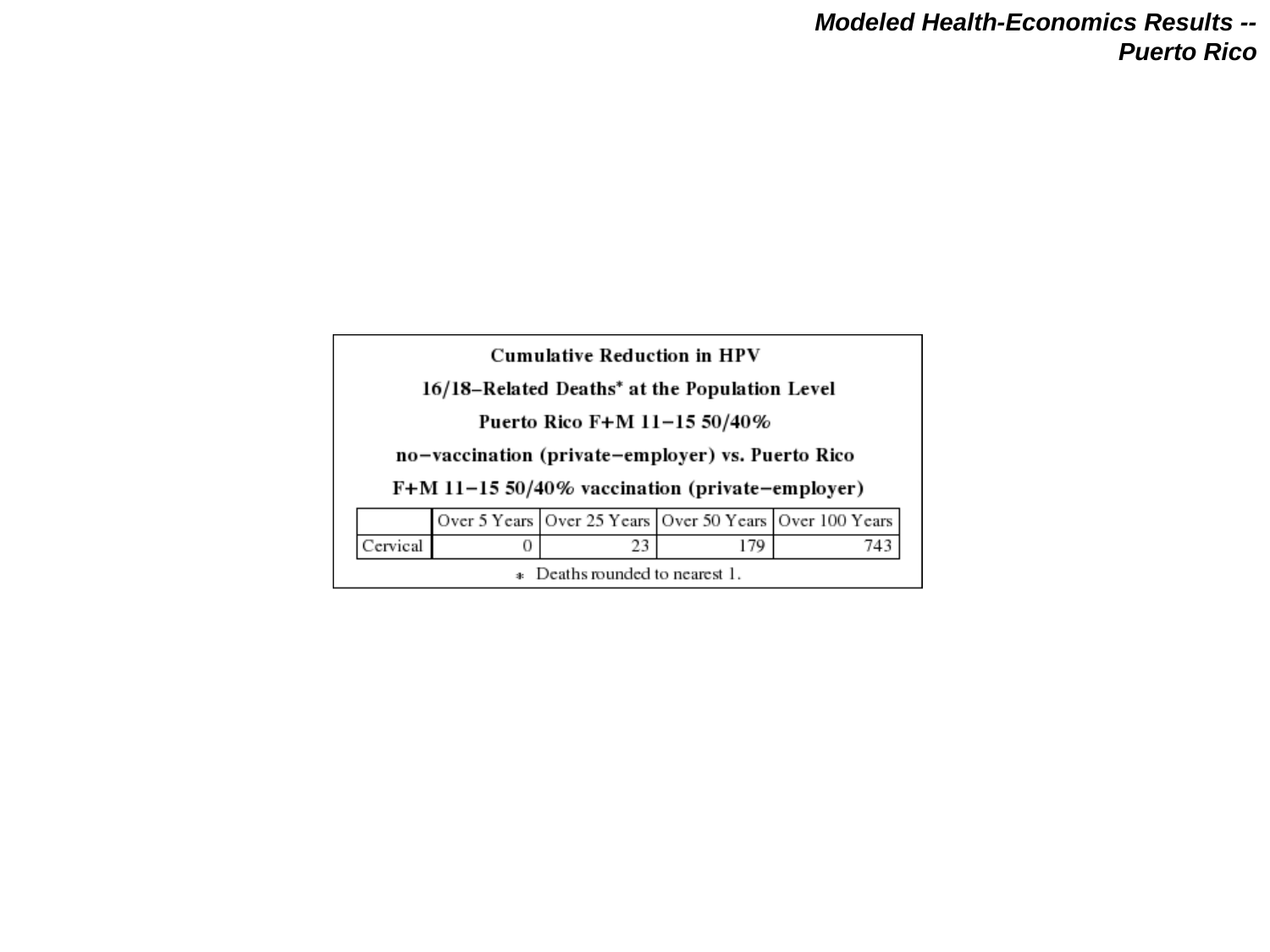

Modeled Health-Economics Results -- Puerto Rico
#

## Slide 23
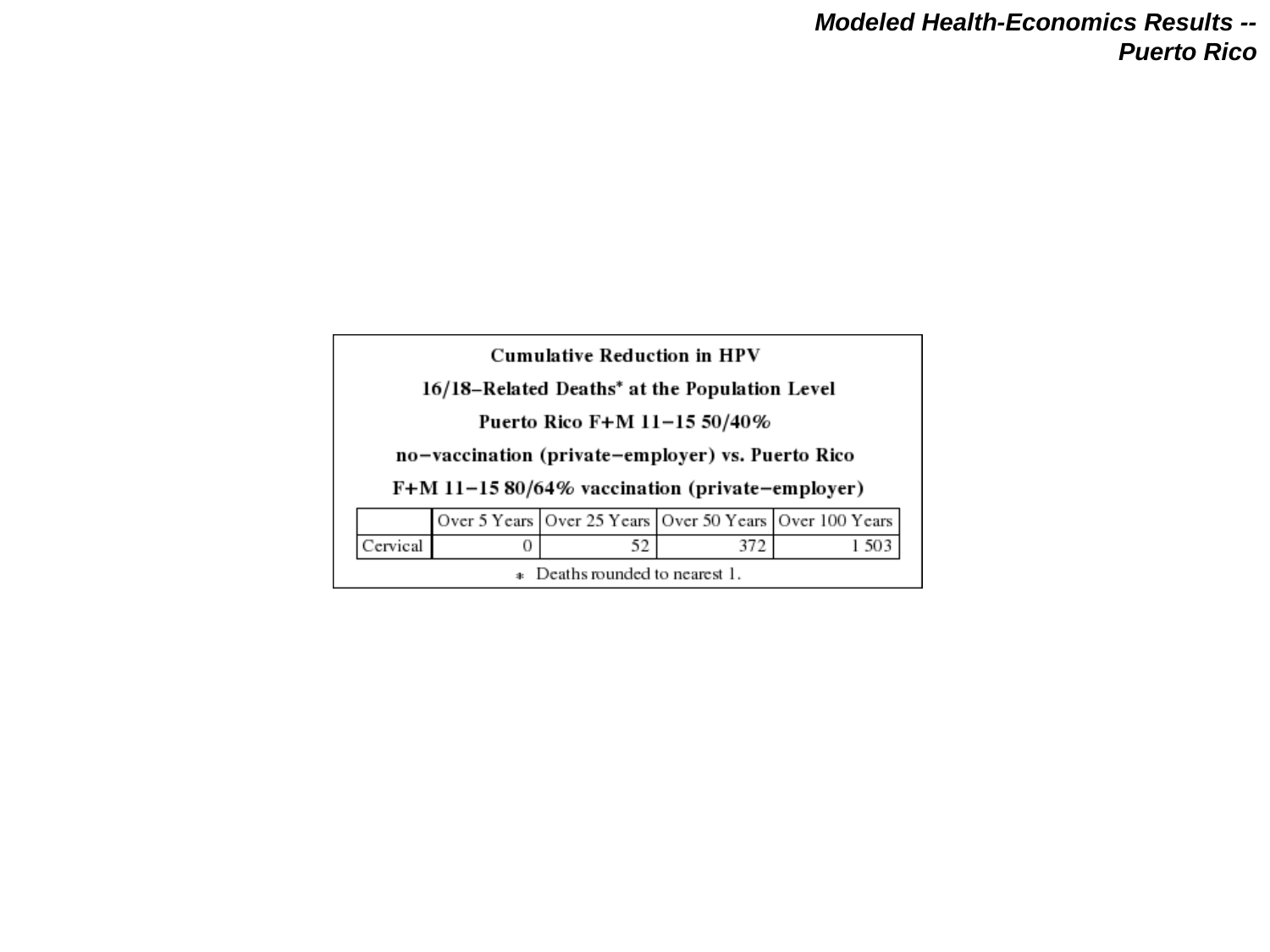

Modeled Health-Economics Results -- Puerto Rico
#

## Slide 24
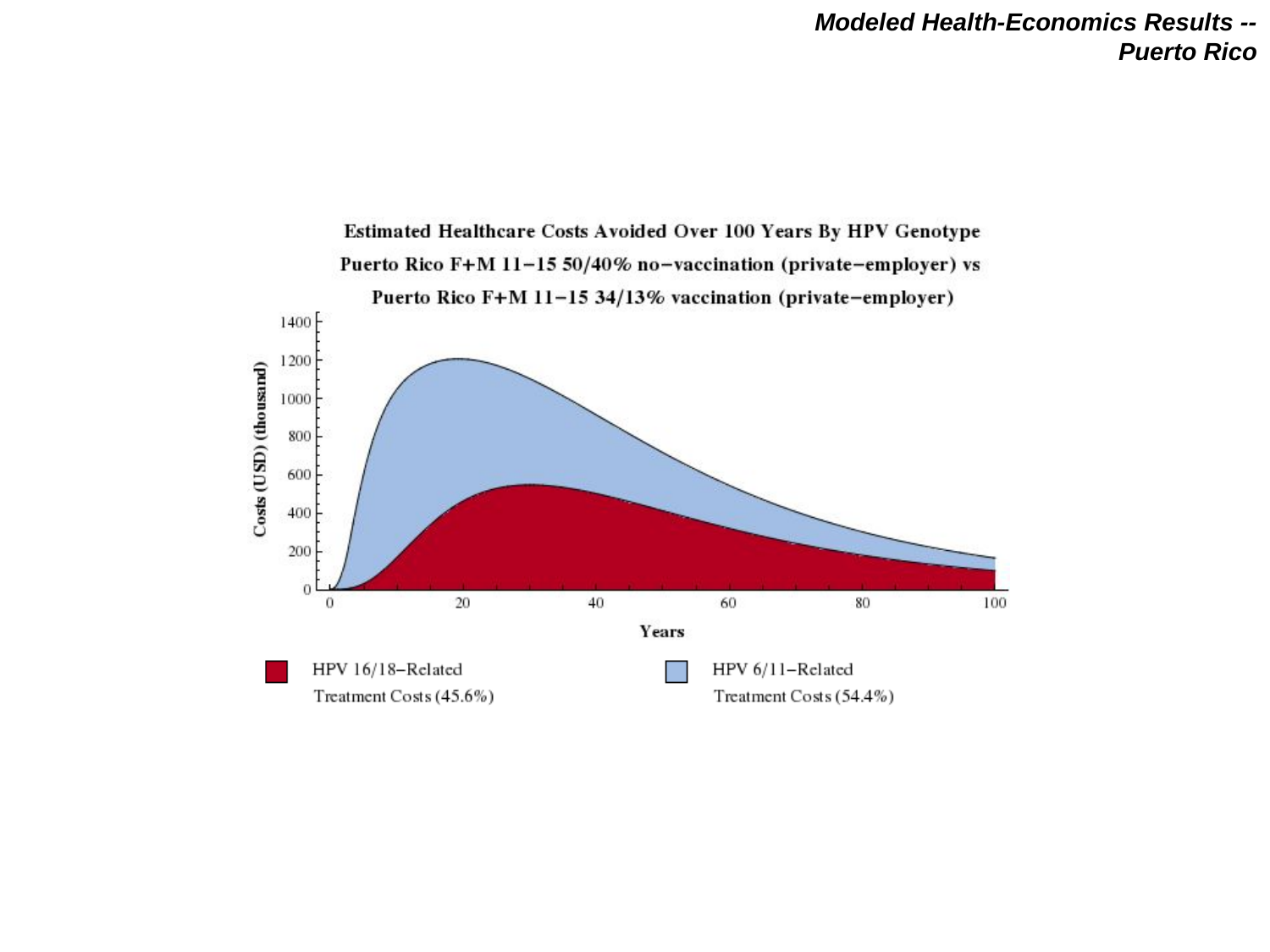

Modeled Health-Economics Results -- Puerto Rico
#

## Slide 25
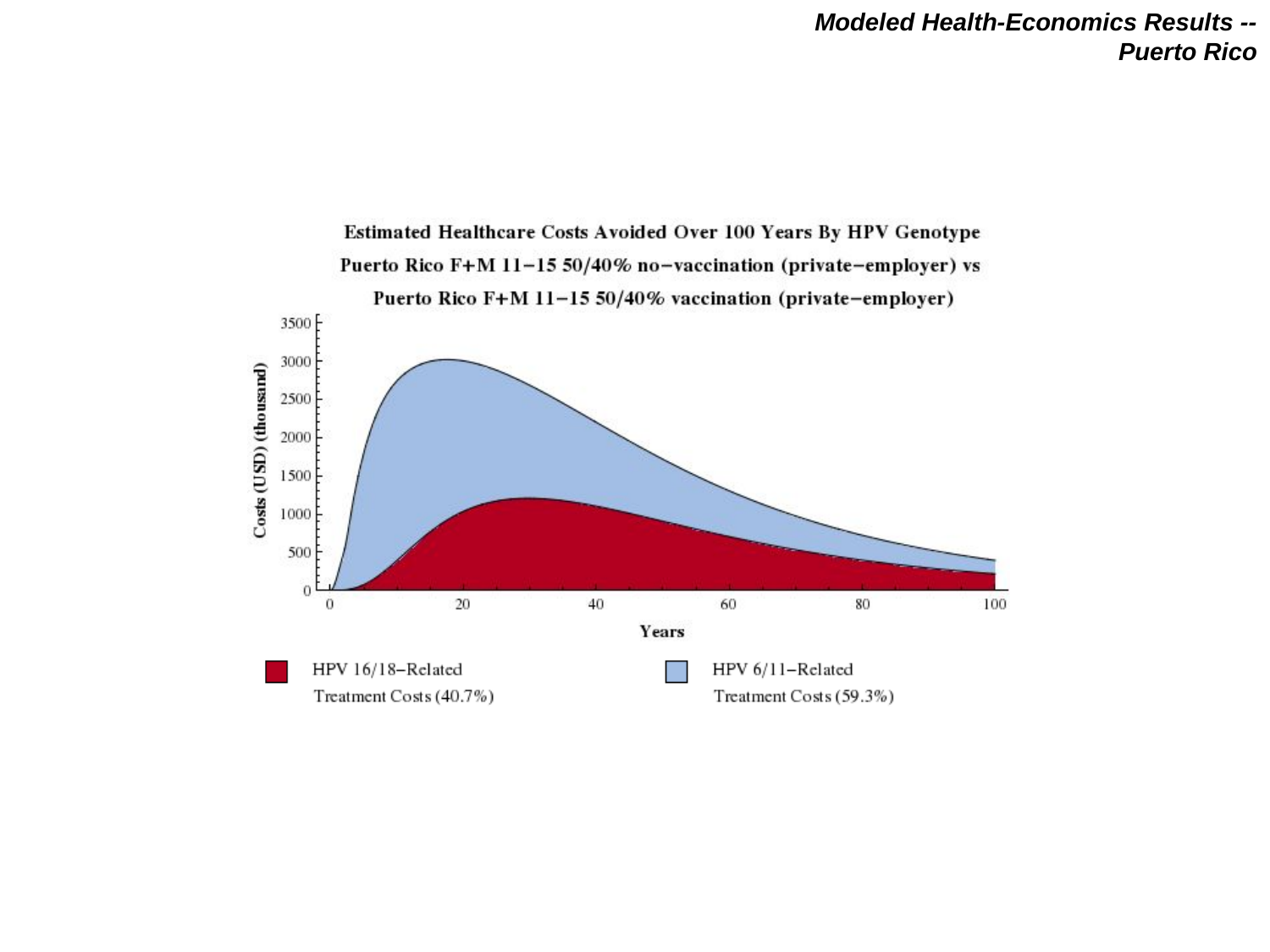

Modeled Health-Economics Results -- Puerto Rico
#

## Slide 26
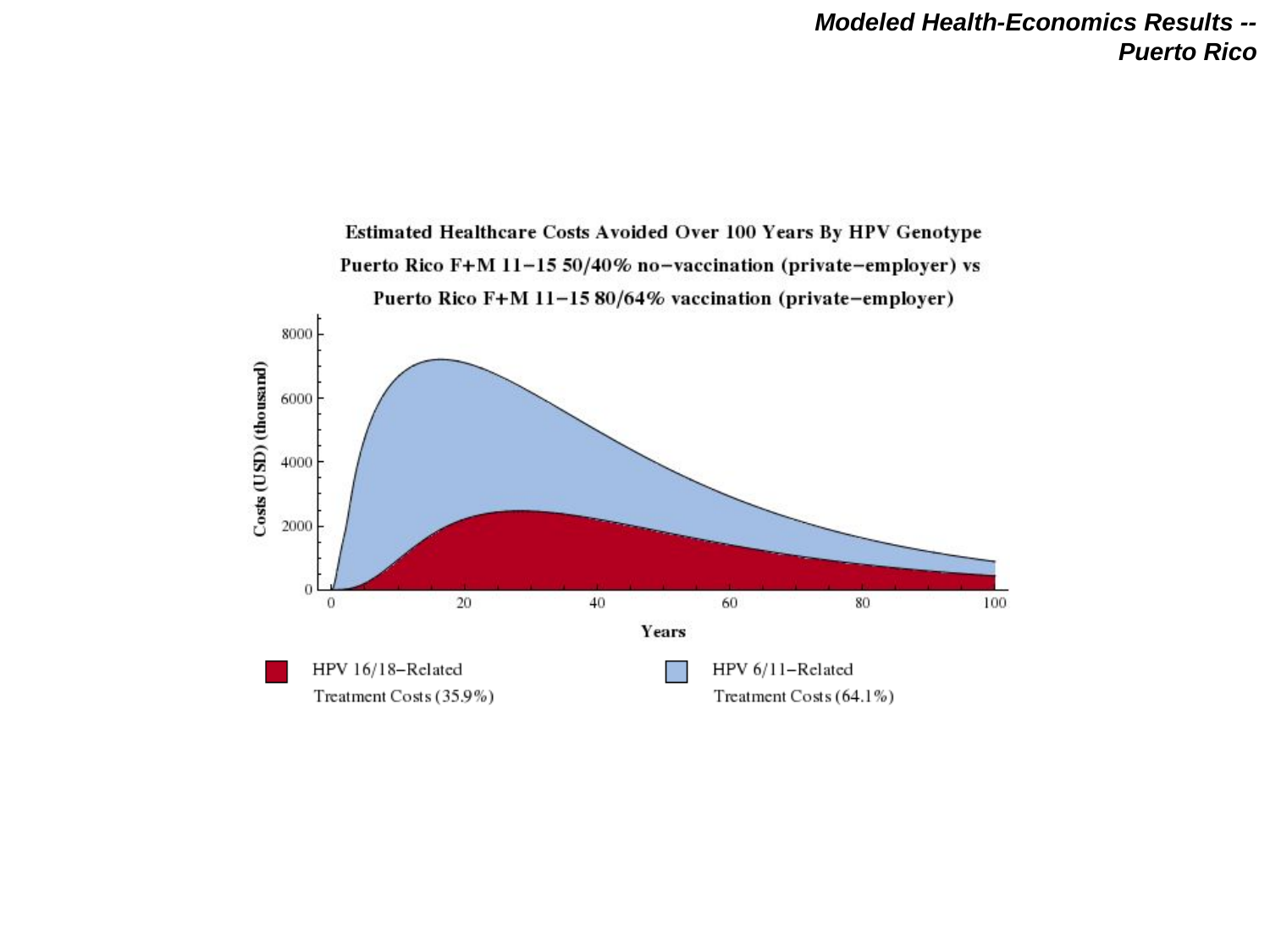

Modeled Health-Economics Results -- Puerto Rico
#

## Slide 27
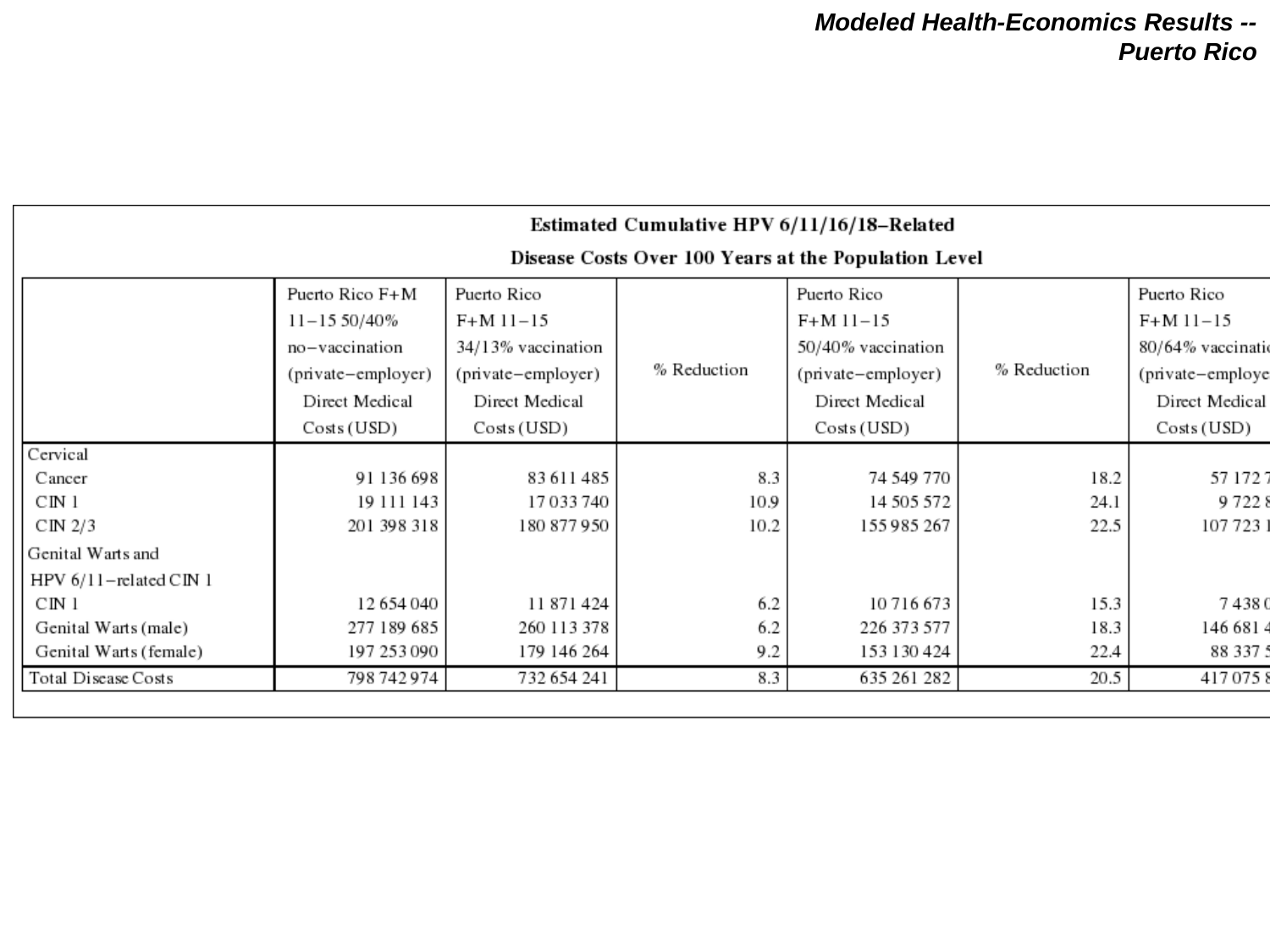

Modeled Health-Economics Results -- Puerto Rico
#

## Slide 28
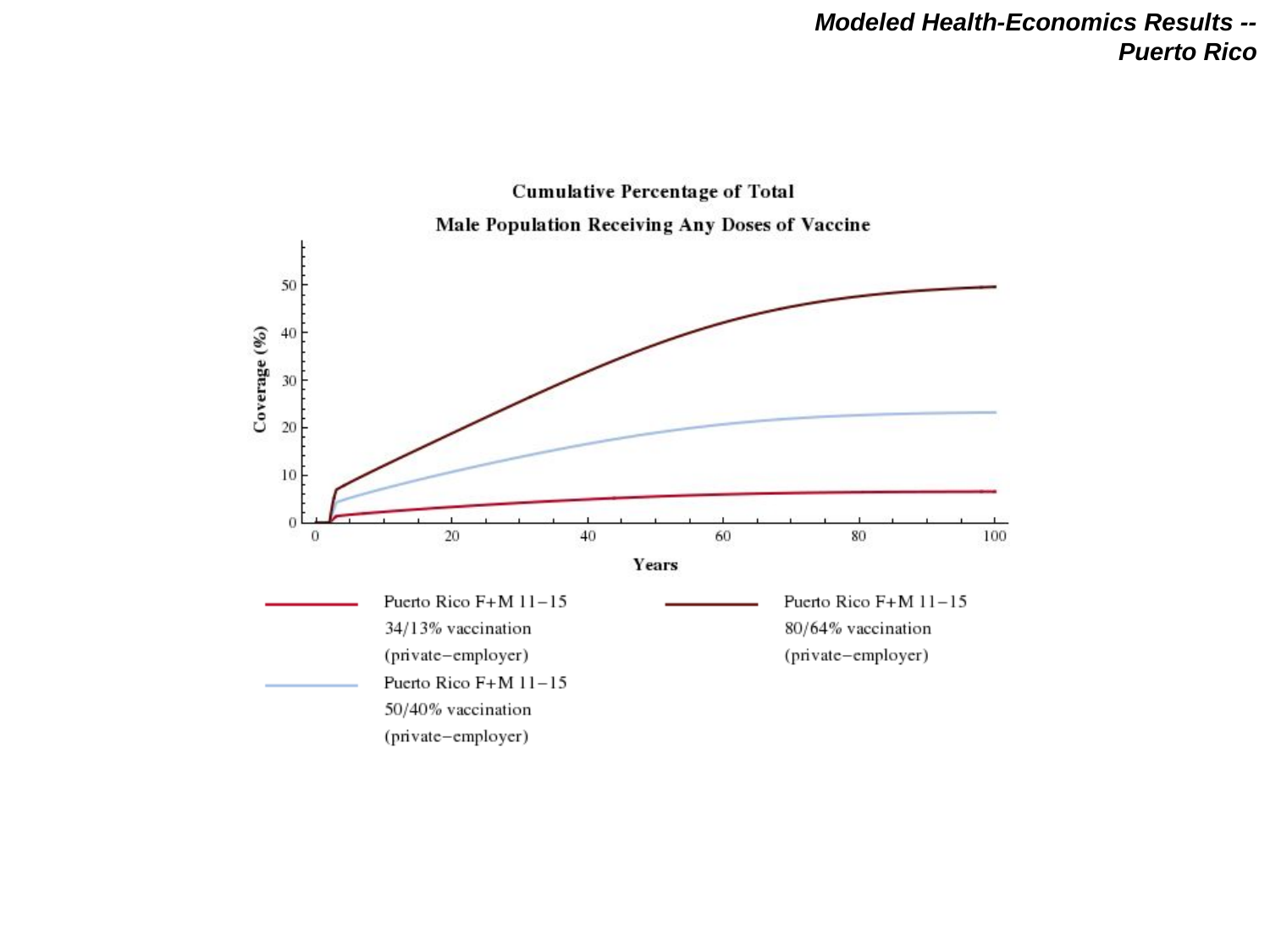

Modeled Health-Economics Results -- Puerto Rico
#

## Slide 29
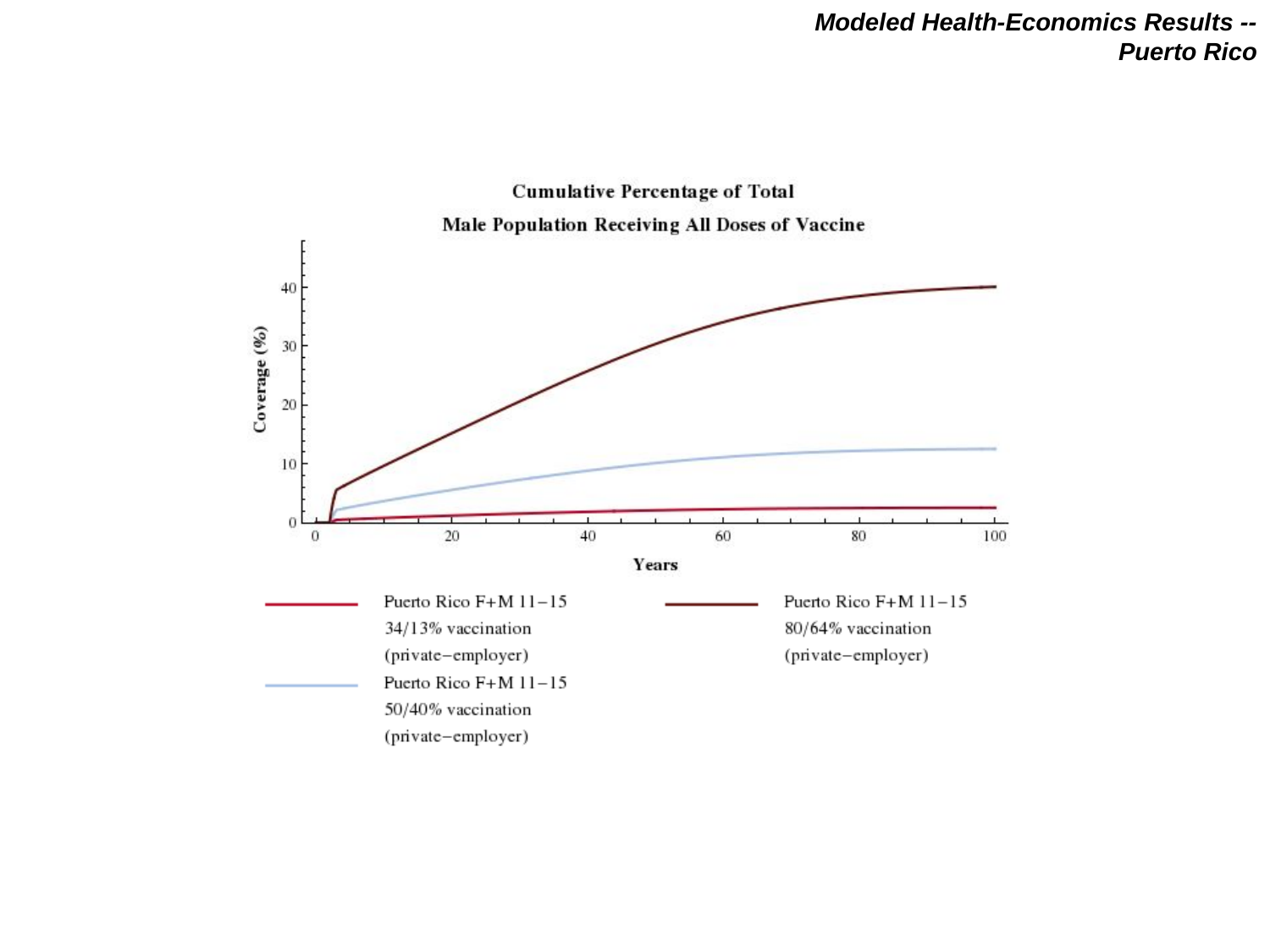

Modeled Health-Economics Results -- Puerto Rico
#

## Slide 30
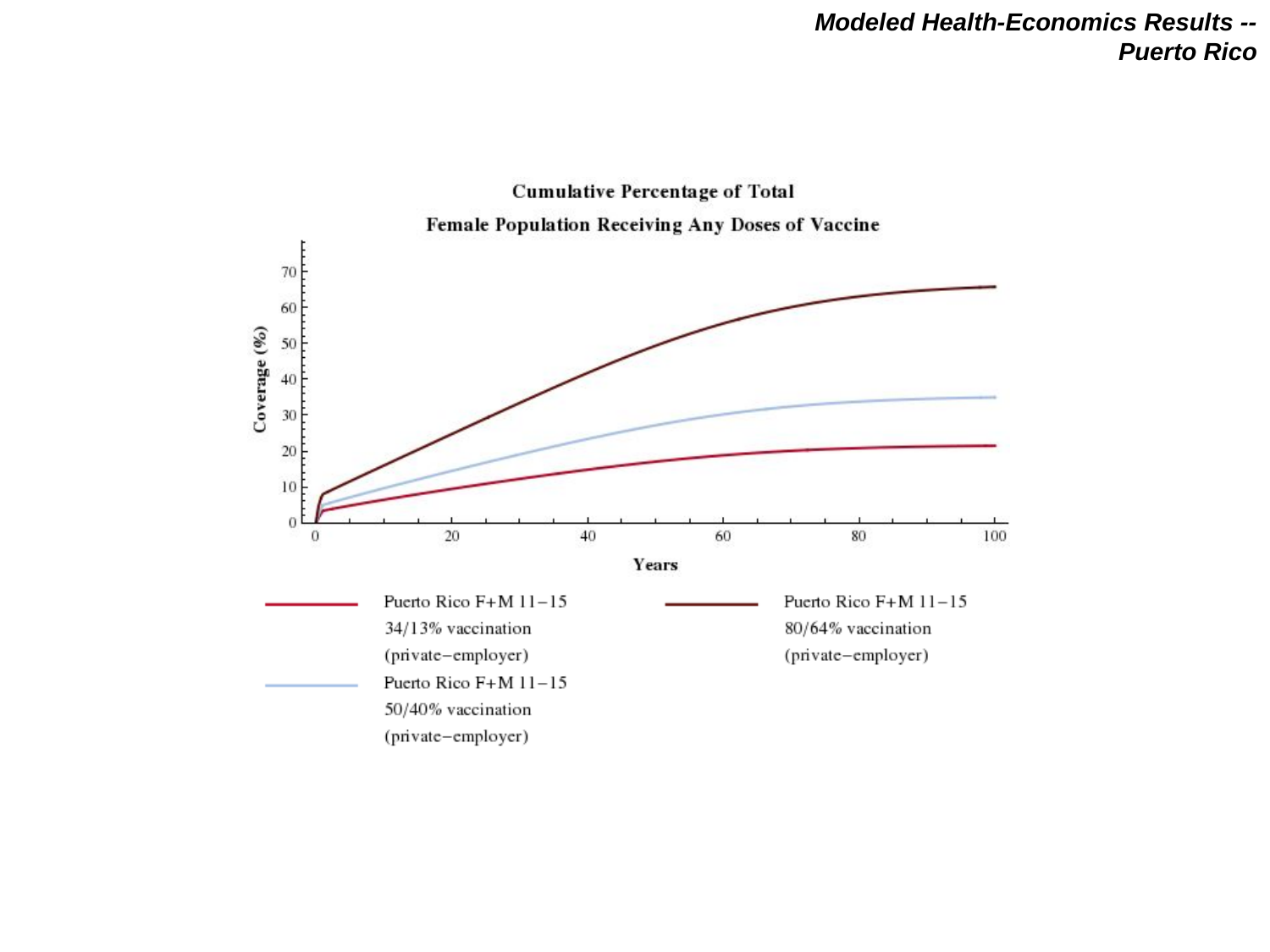

Modeled Health-Economics Results -- Puerto Rico
#

## Slide 31
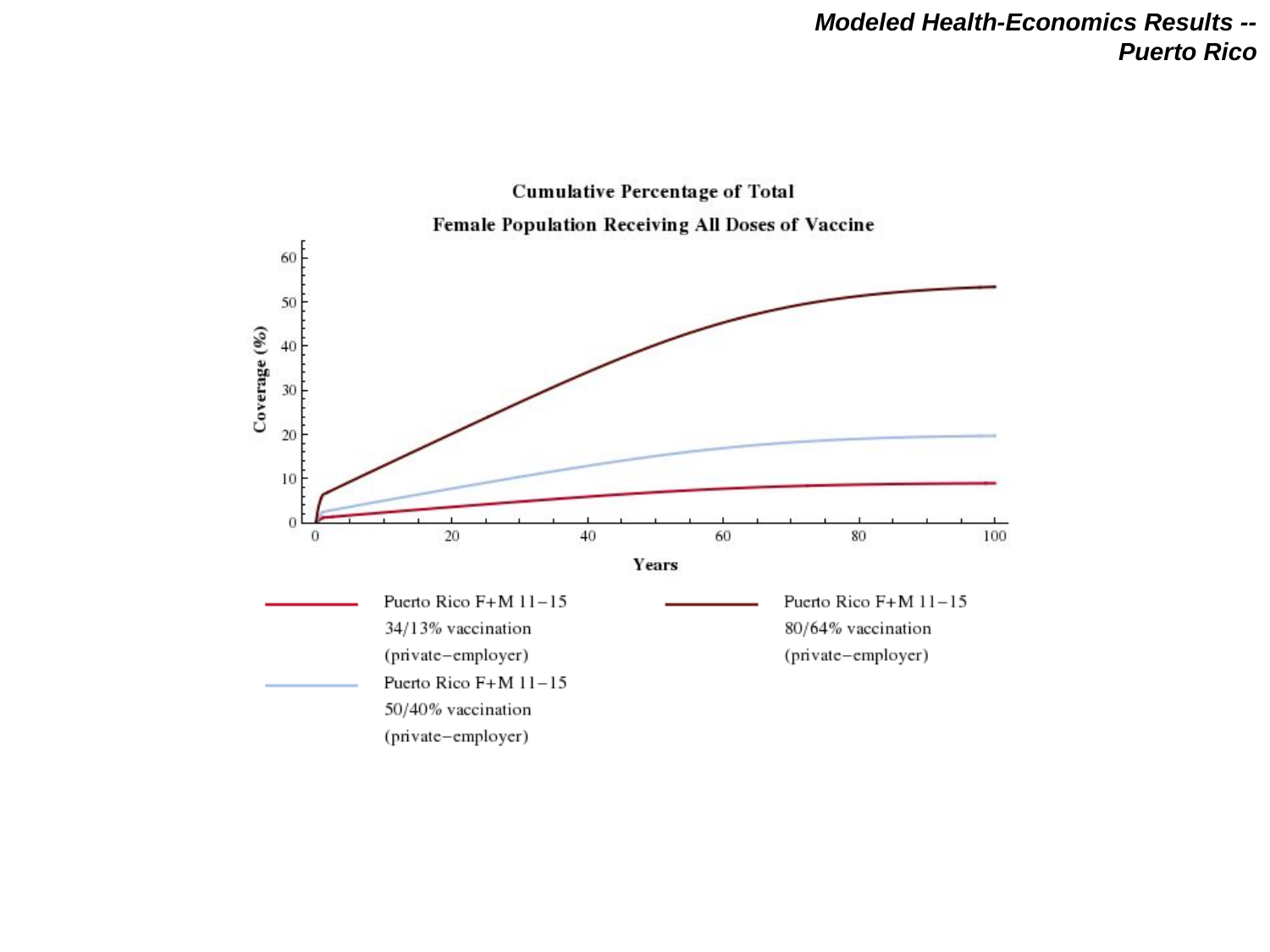

Modeled Health-Economics Results -- Puerto Rico
#

## Slide 32
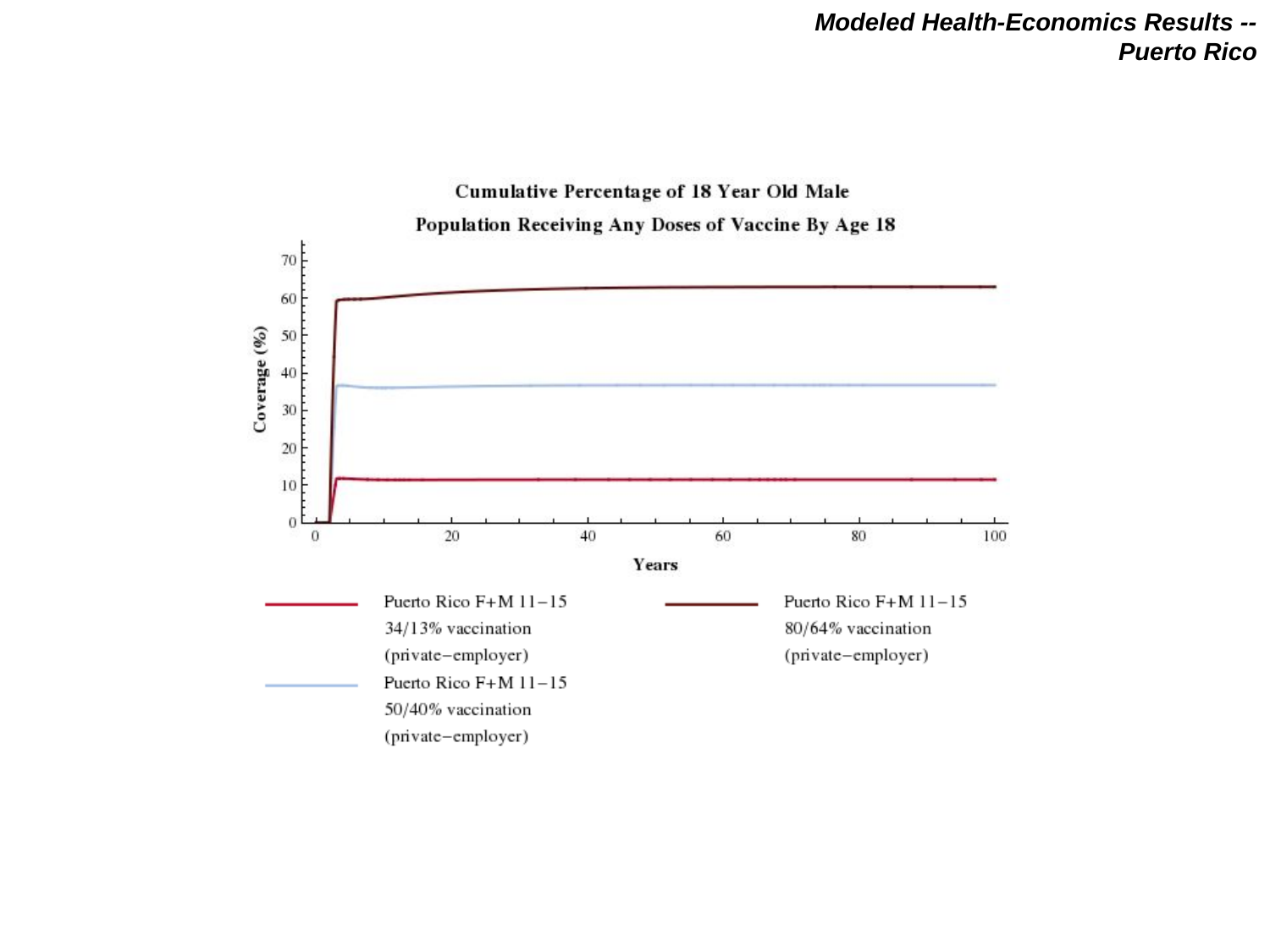

Modeled Health-Economics Results -- Puerto Rico
#

## Slide 33
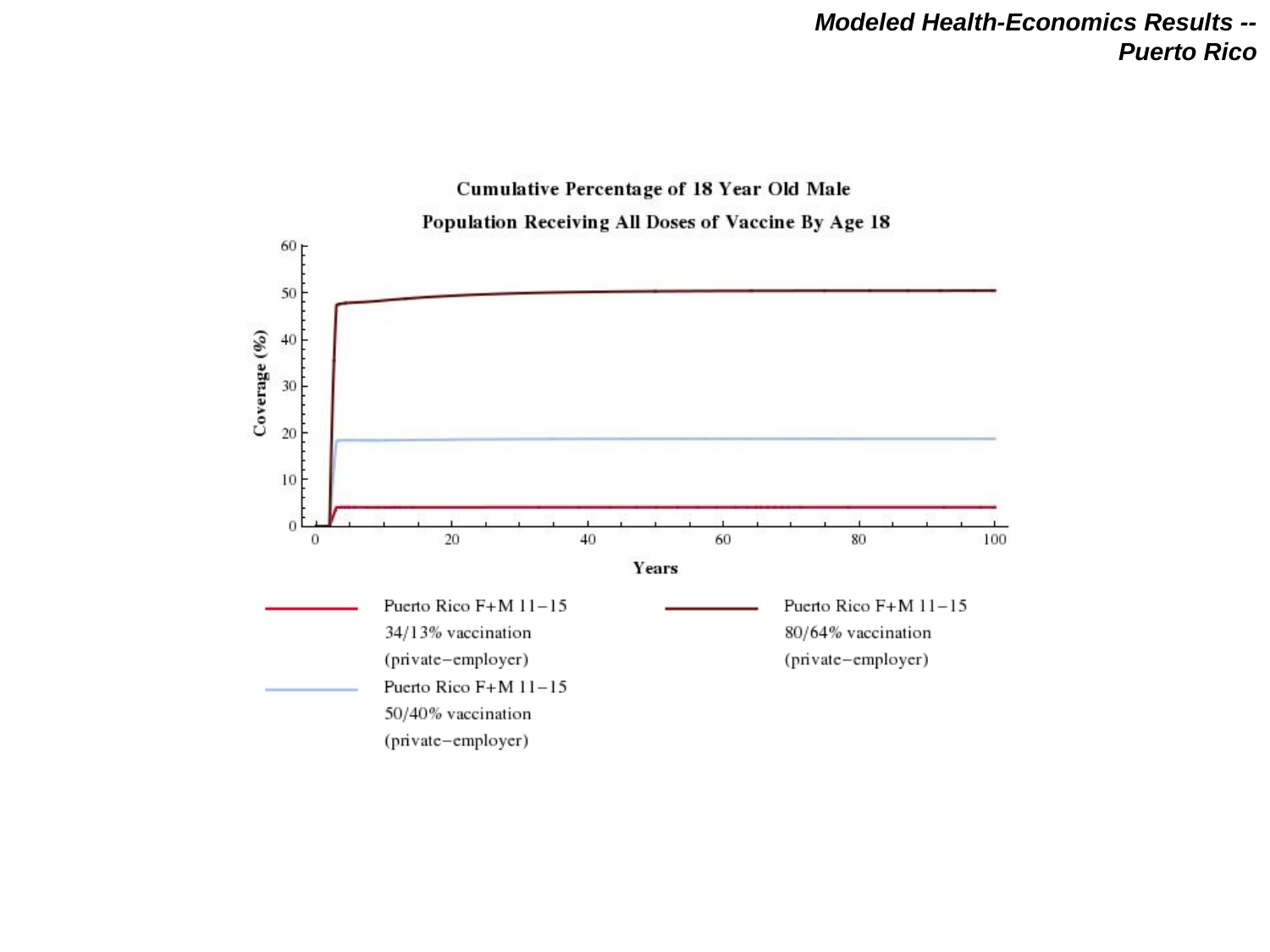

Modeled Health-Economics Results -- Puerto Rico
#

## Slide 34
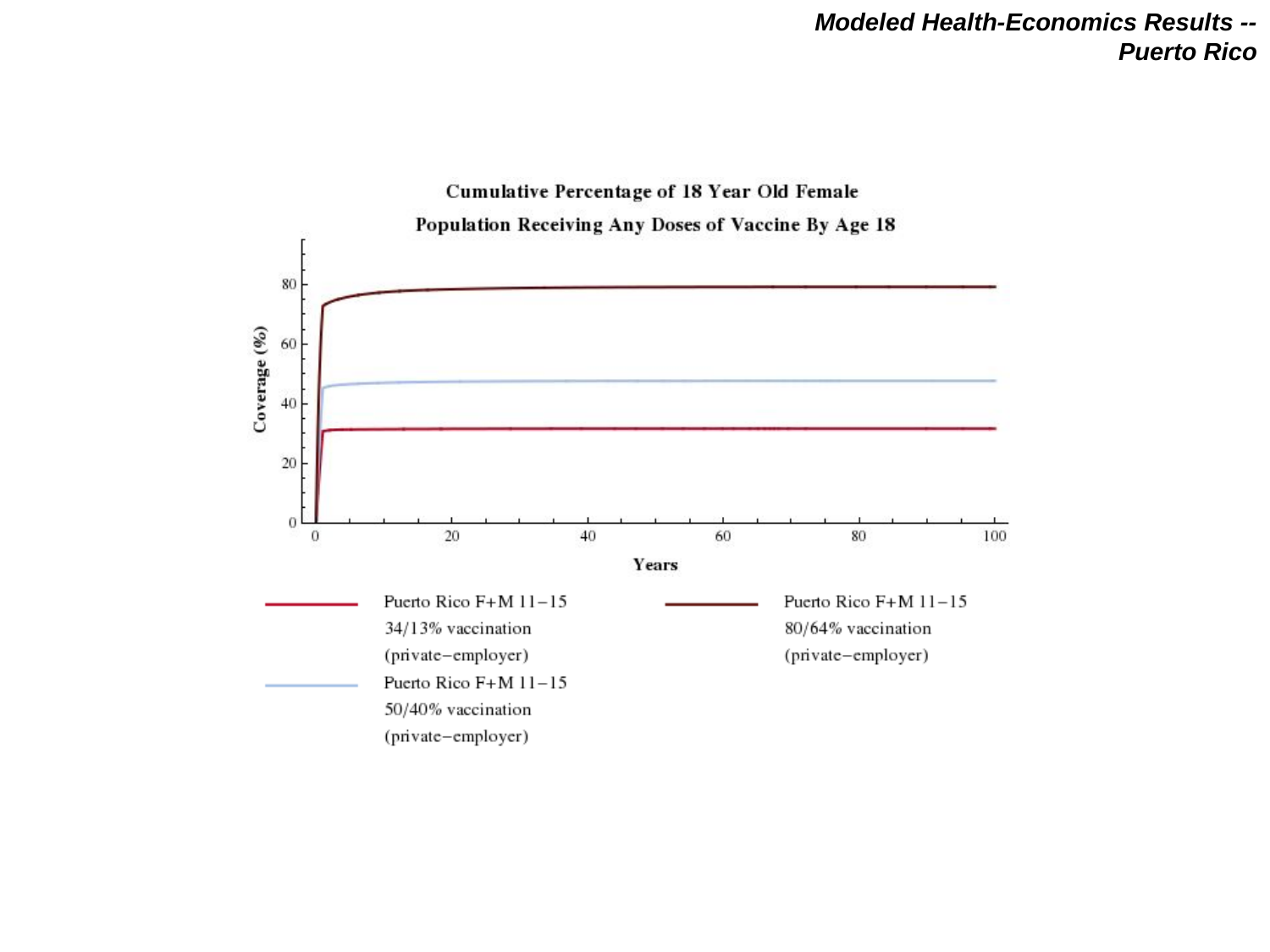

Modeled Health-Economics Results -- Puerto Rico
#

## Slide 35
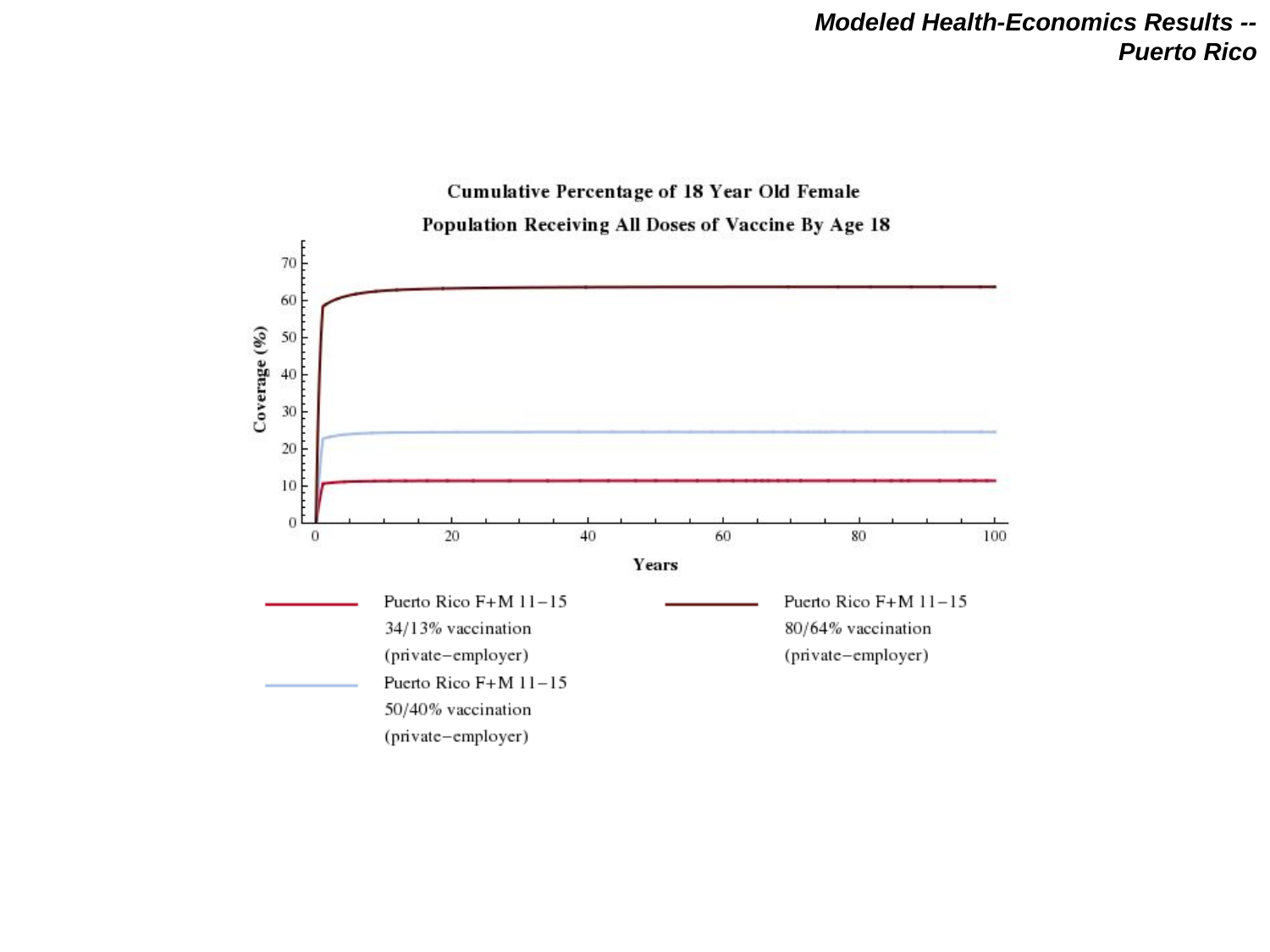

Modeled Health-Economics Results -- Puerto Rico
#

## Slide 36
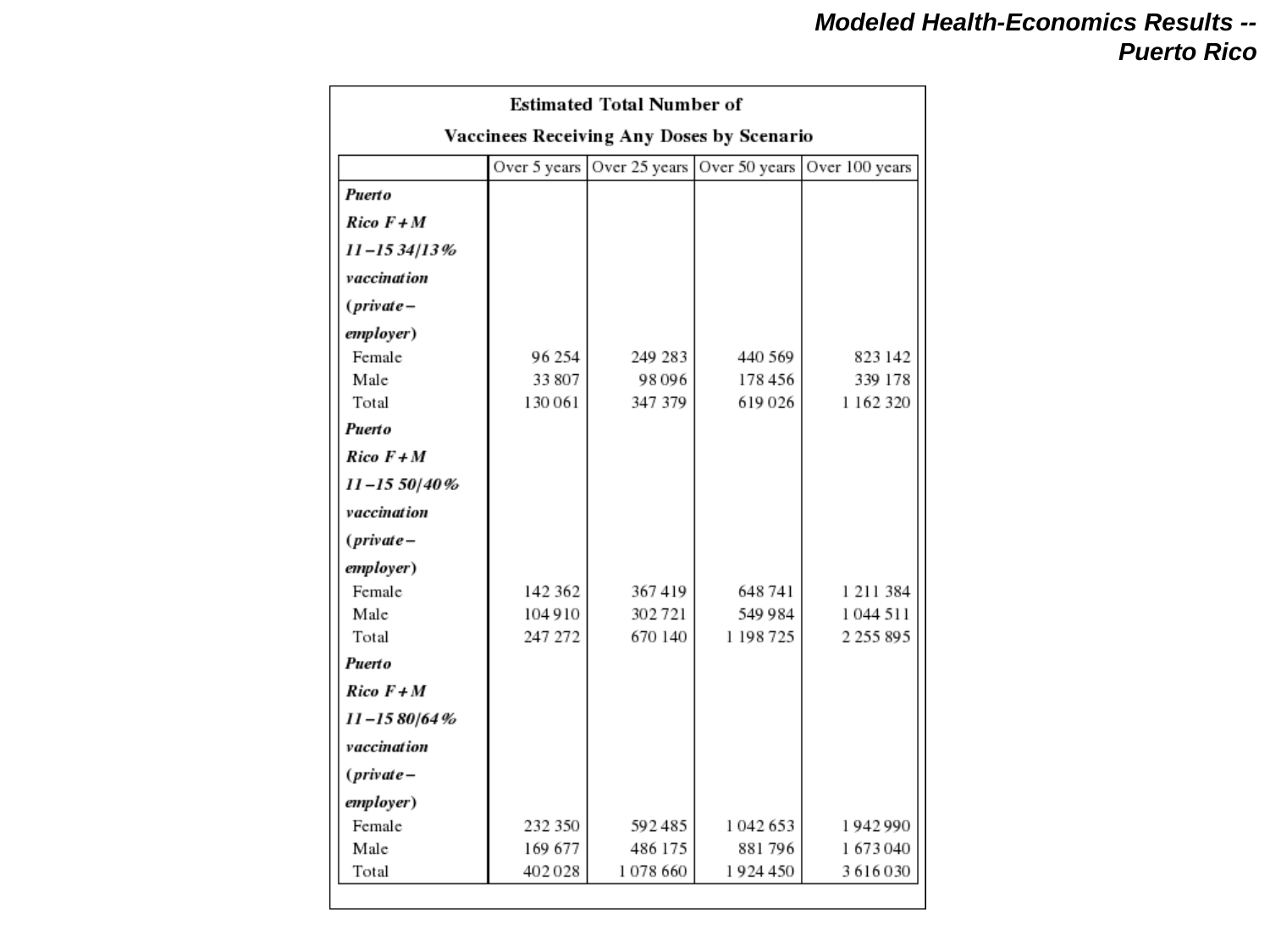

Modeled Health-Economics Results -- Puerto Rico
#

## Slide 37
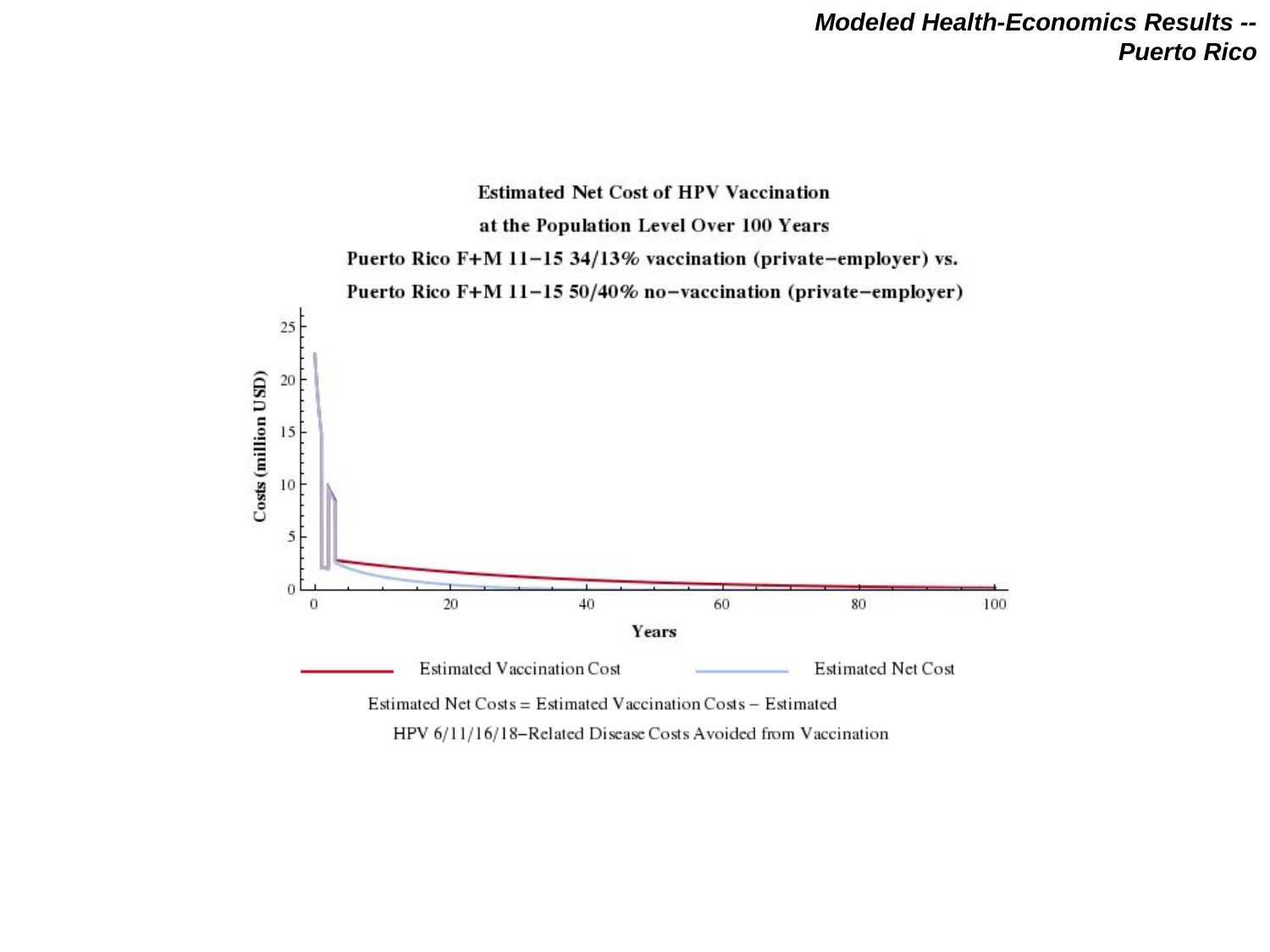

Modeled Health-Economics Results -- Puerto Rico
#

## Slide 38
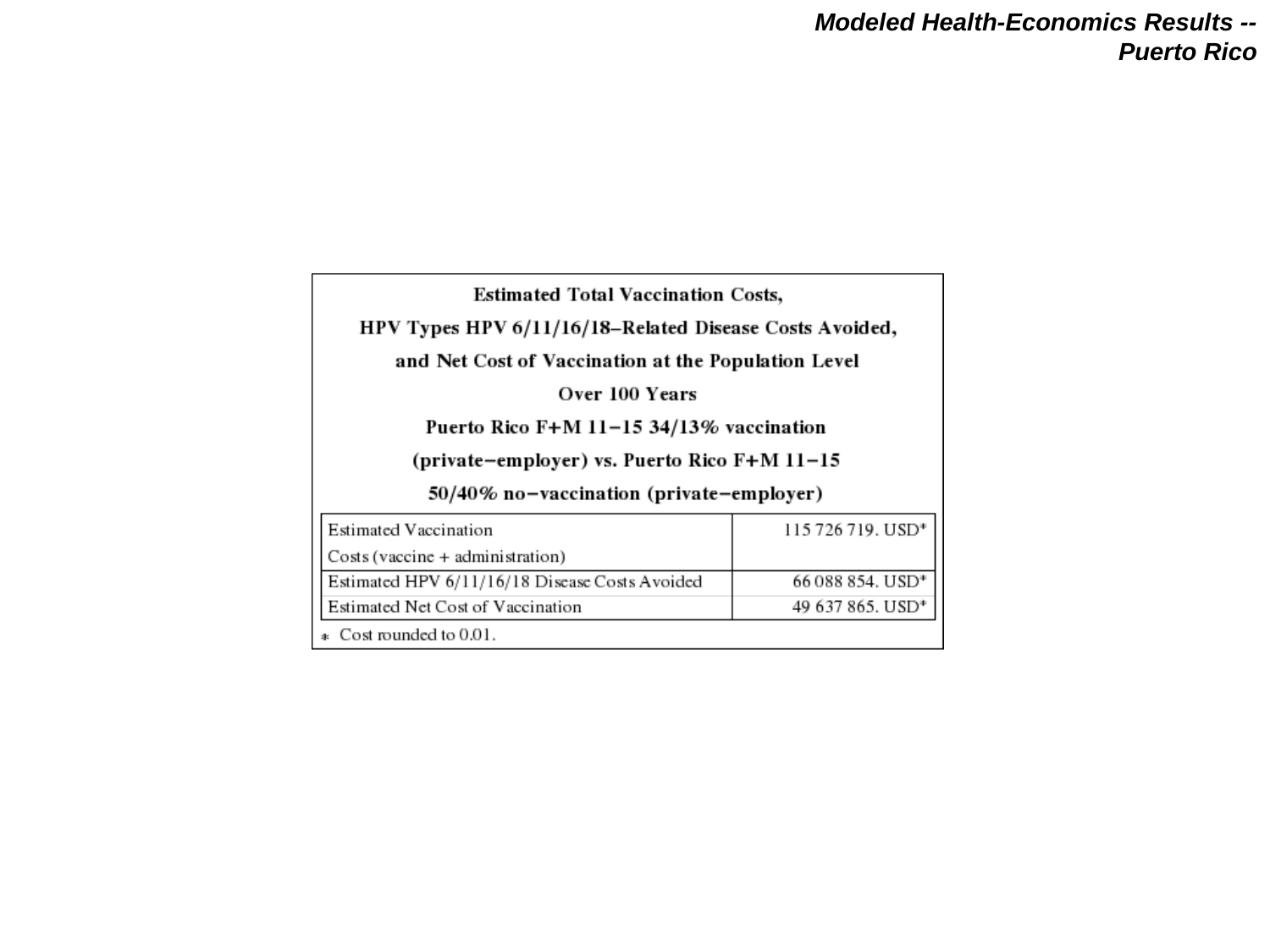

Modeled Health-Economics Results -- Puerto Rico
#

## Slide 39
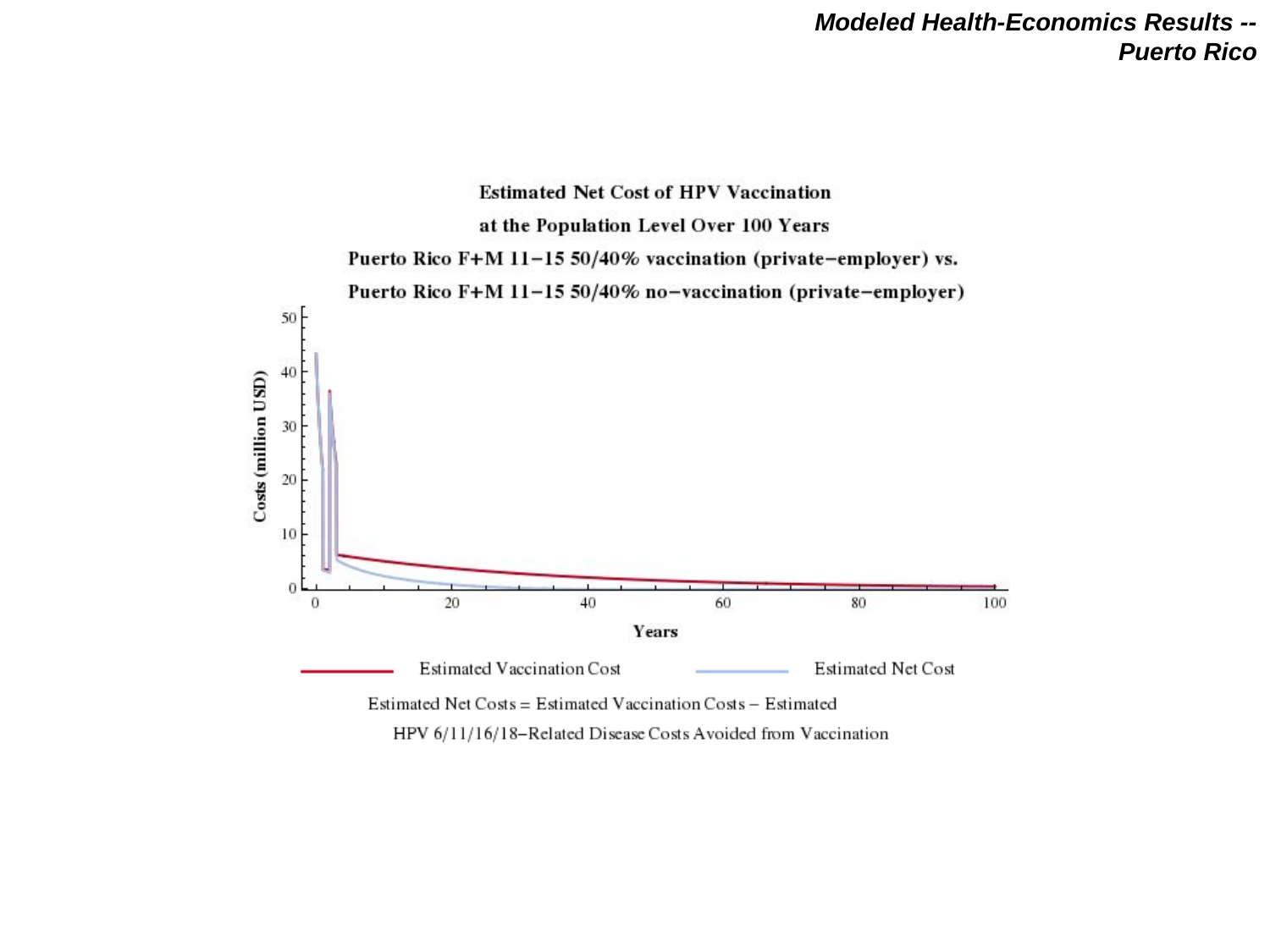

Modeled Health-Economics Results -- Puerto Rico
#

## Slide 40
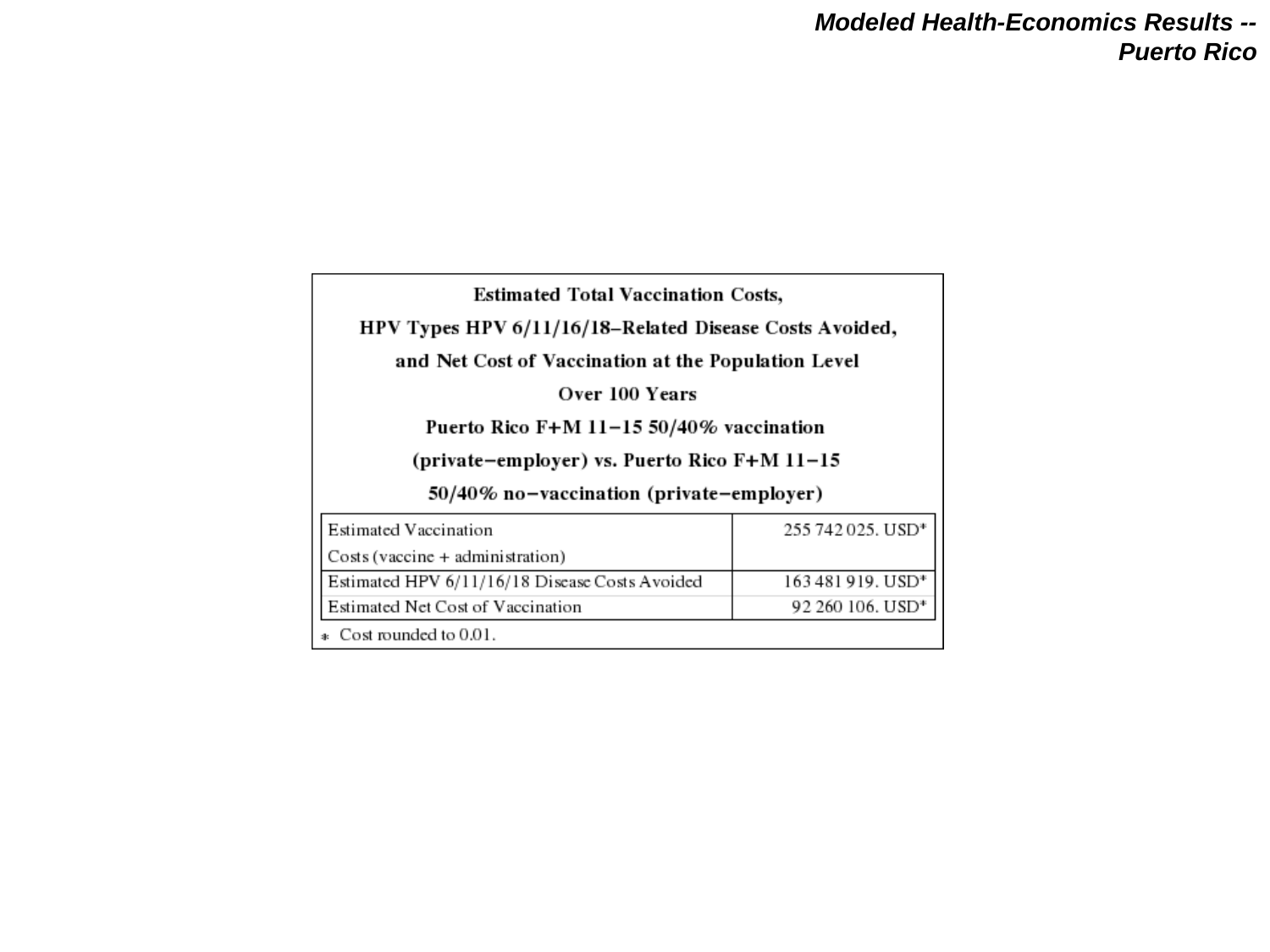

Modeled Health-Economics Results -- Puerto Rico
#

## Slide 41
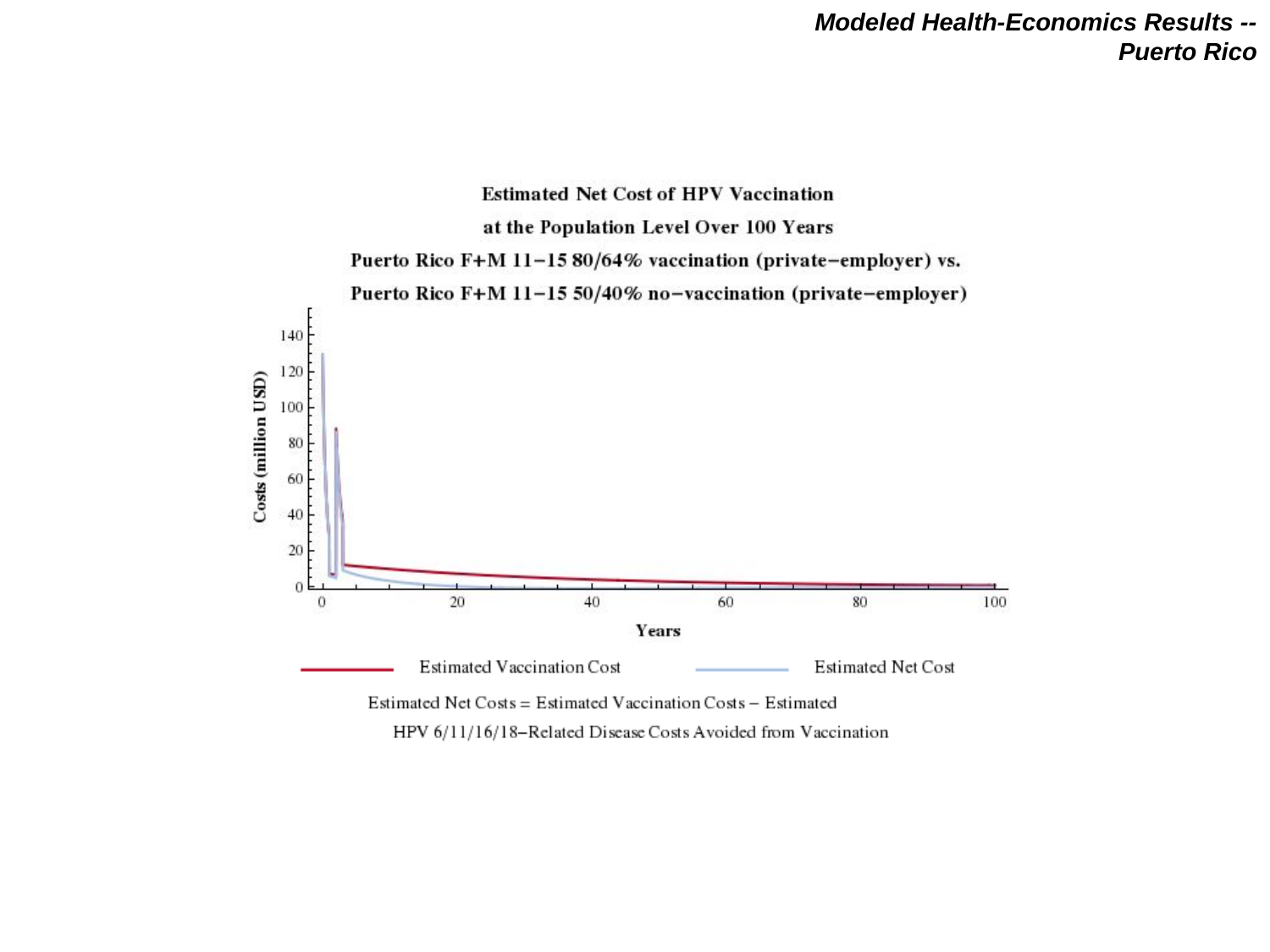

Modeled Health-Economics Results -- Puerto Rico
#

## Slide 42
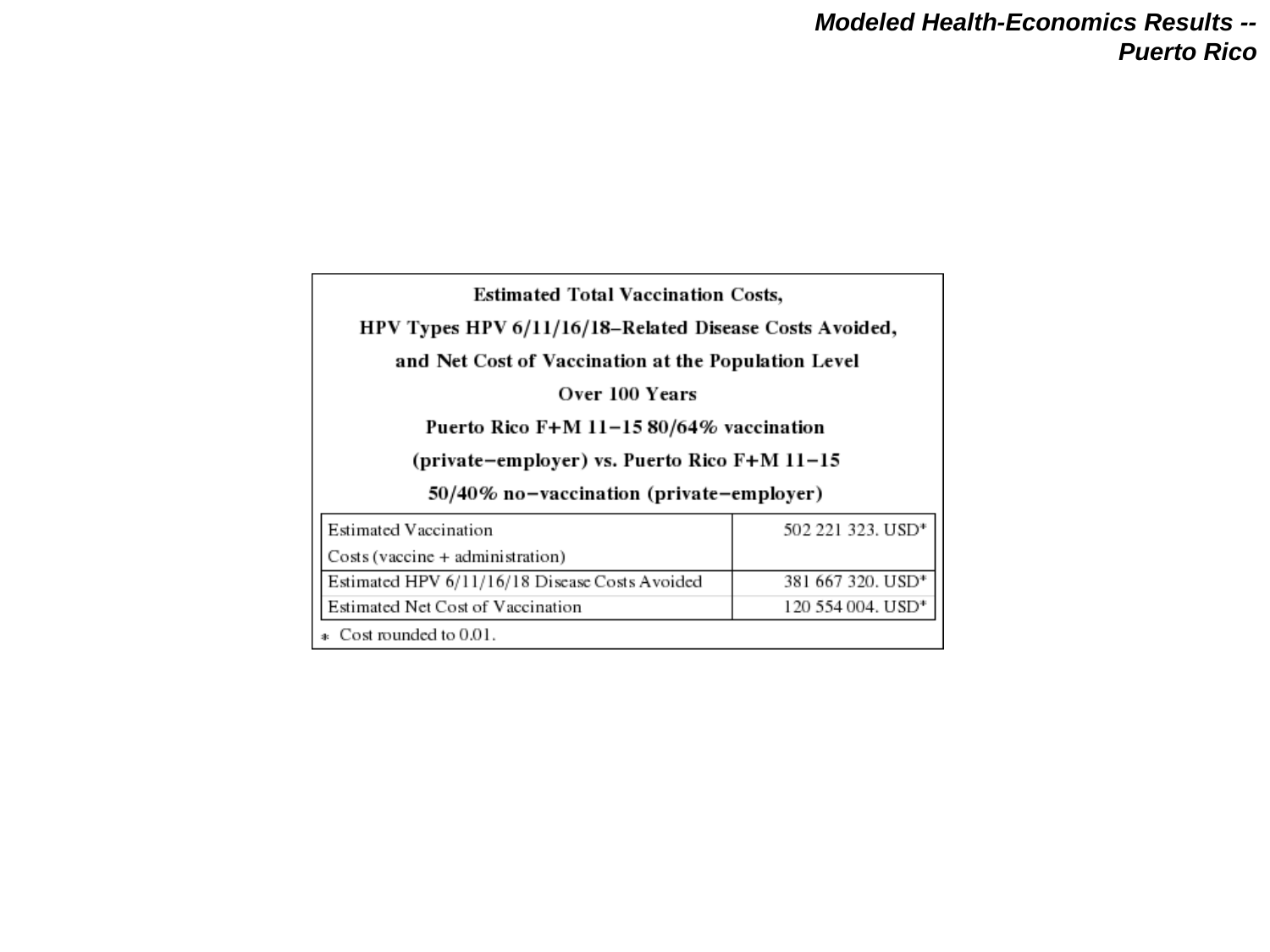

Modeled Health-Economics Results -- Puerto Rico
#

## Slide 43
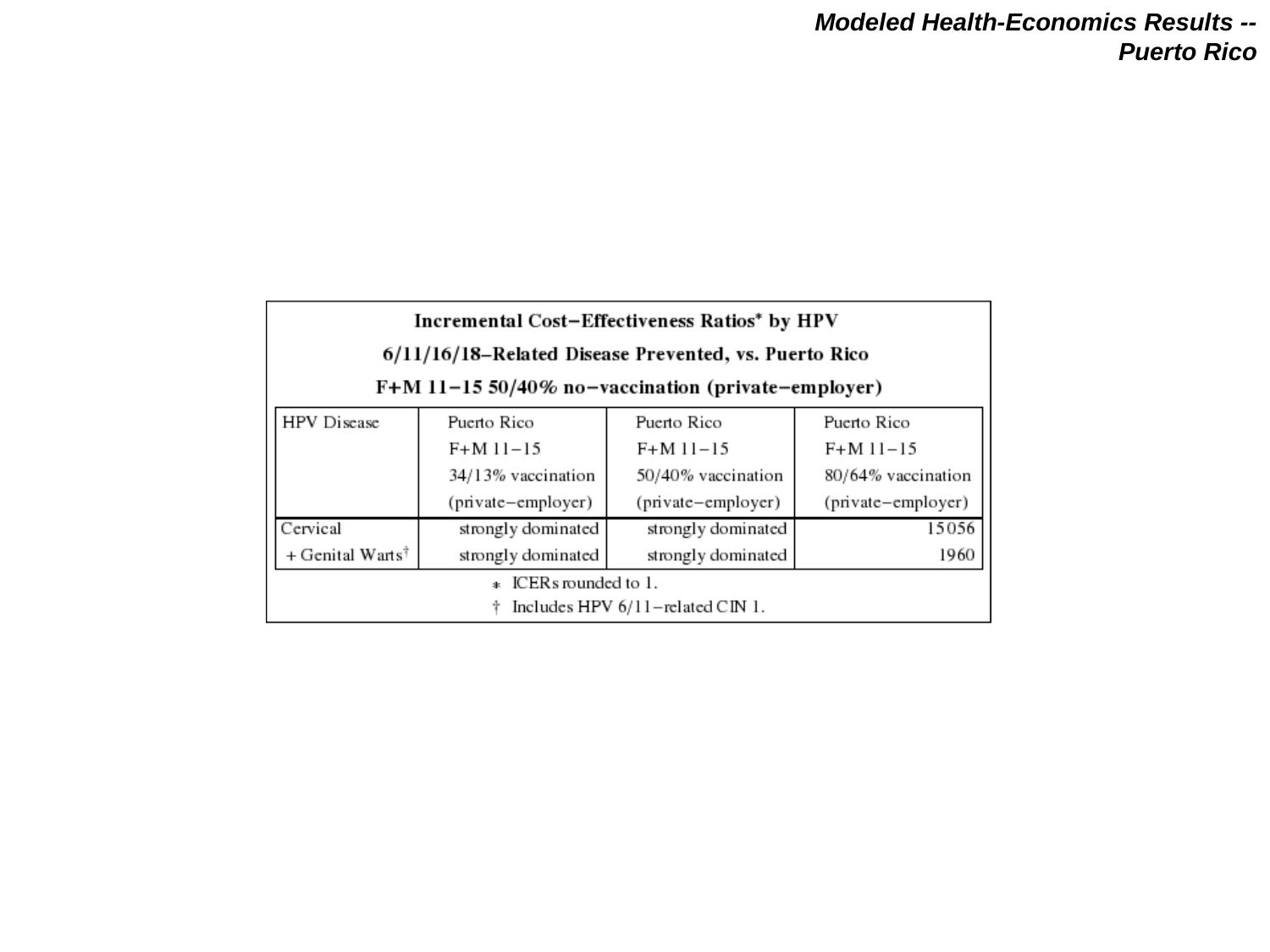

Modeled Health-Economics Results -- Puerto Rico
#

## Slide 44
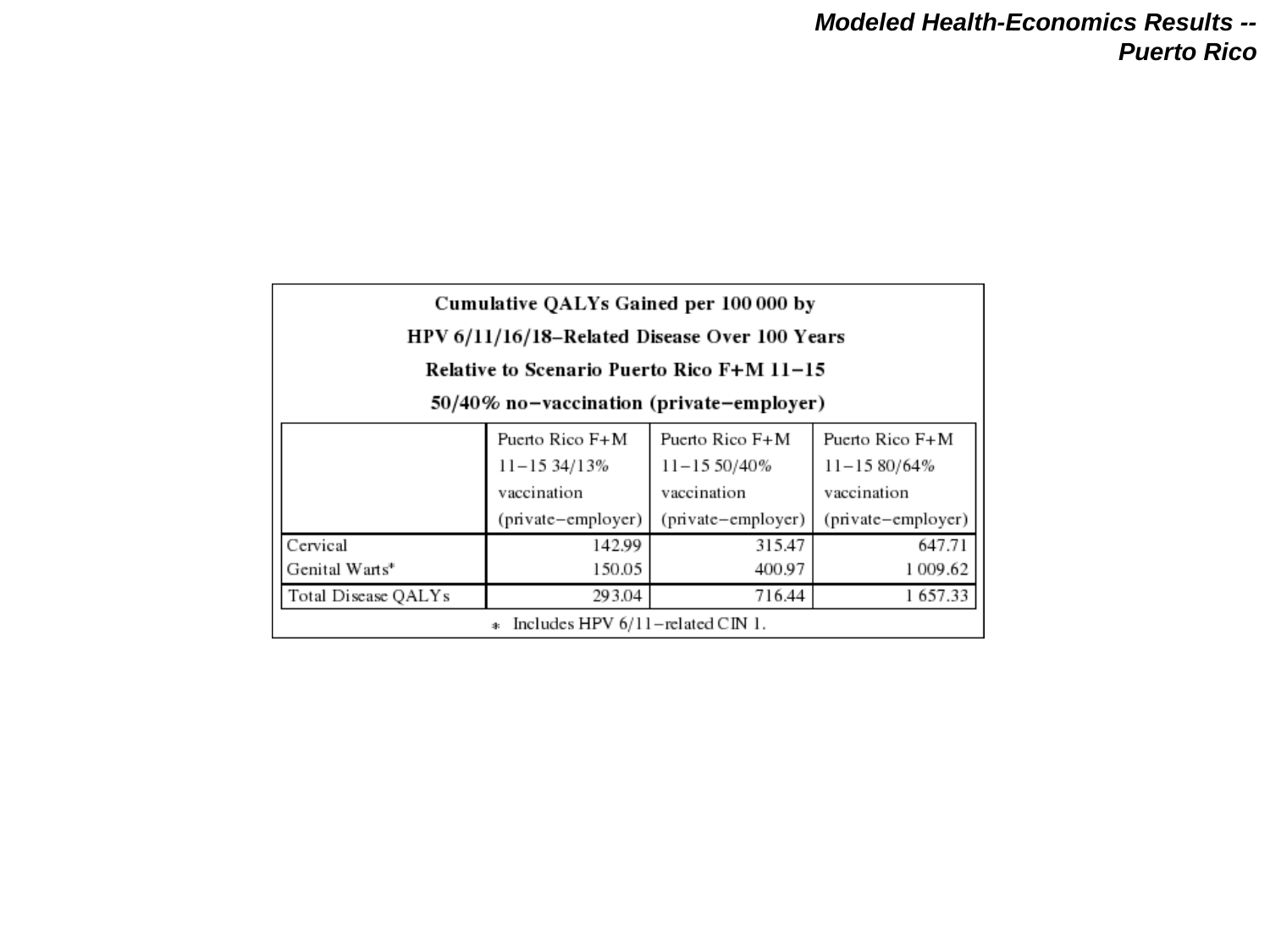

Modeled Health-Economics Results -- Puerto Rico
#

## Slide 45
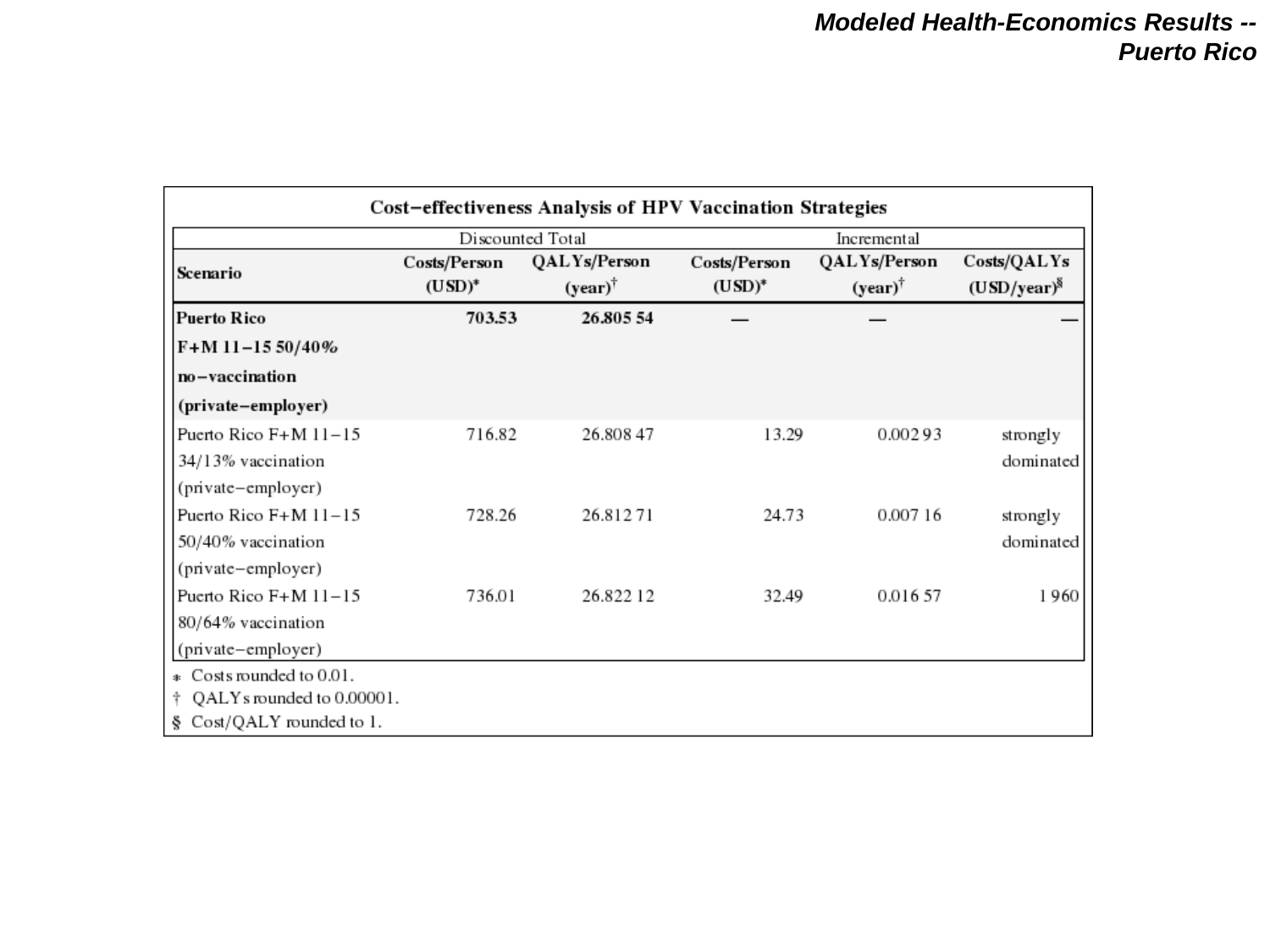

Modeled Health-Economics Results -- Puerto Rico
#

## Slide 46
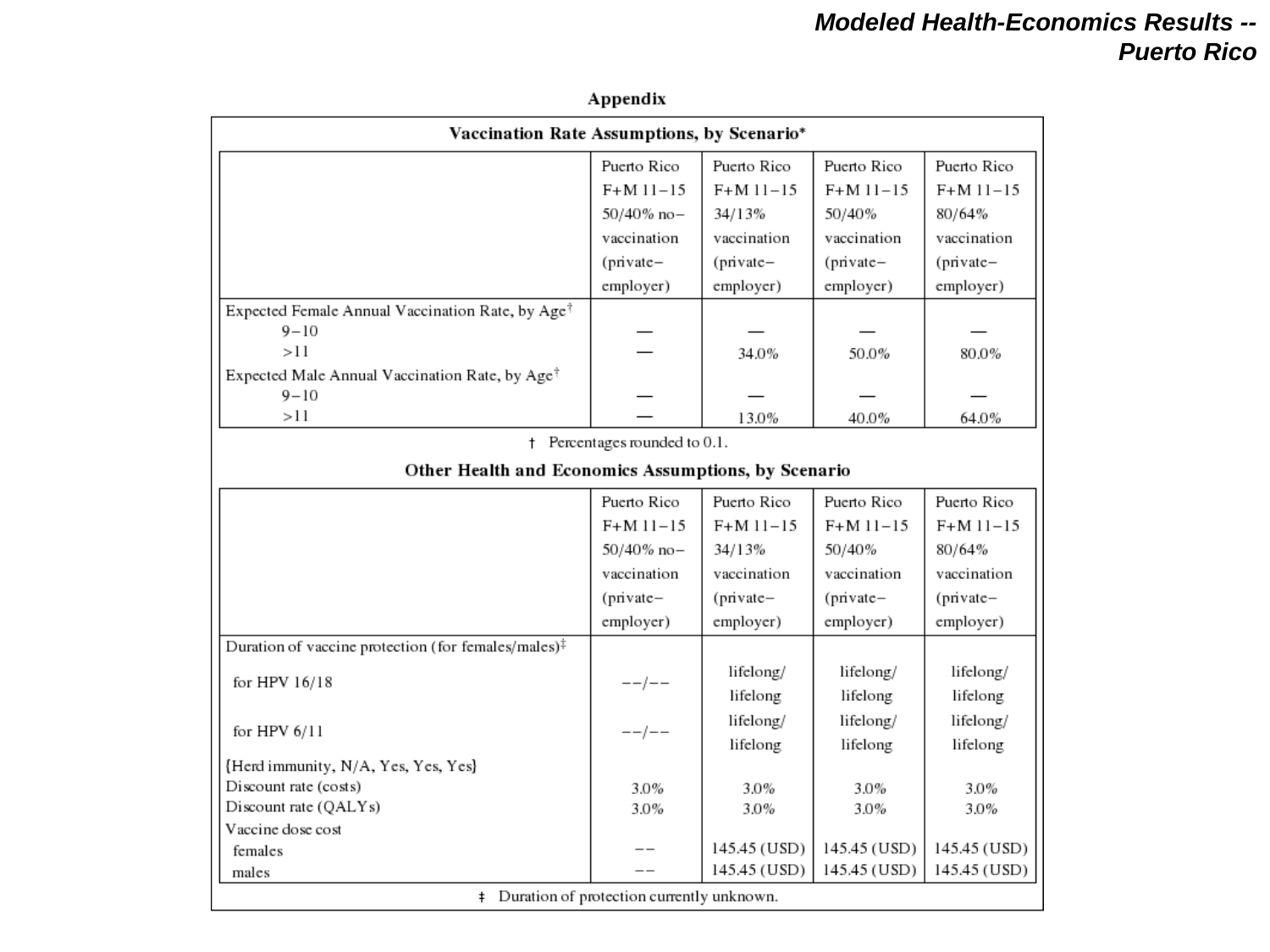

Modeled Health-Economics Results -- Puerto Rico
#

## Slide 47
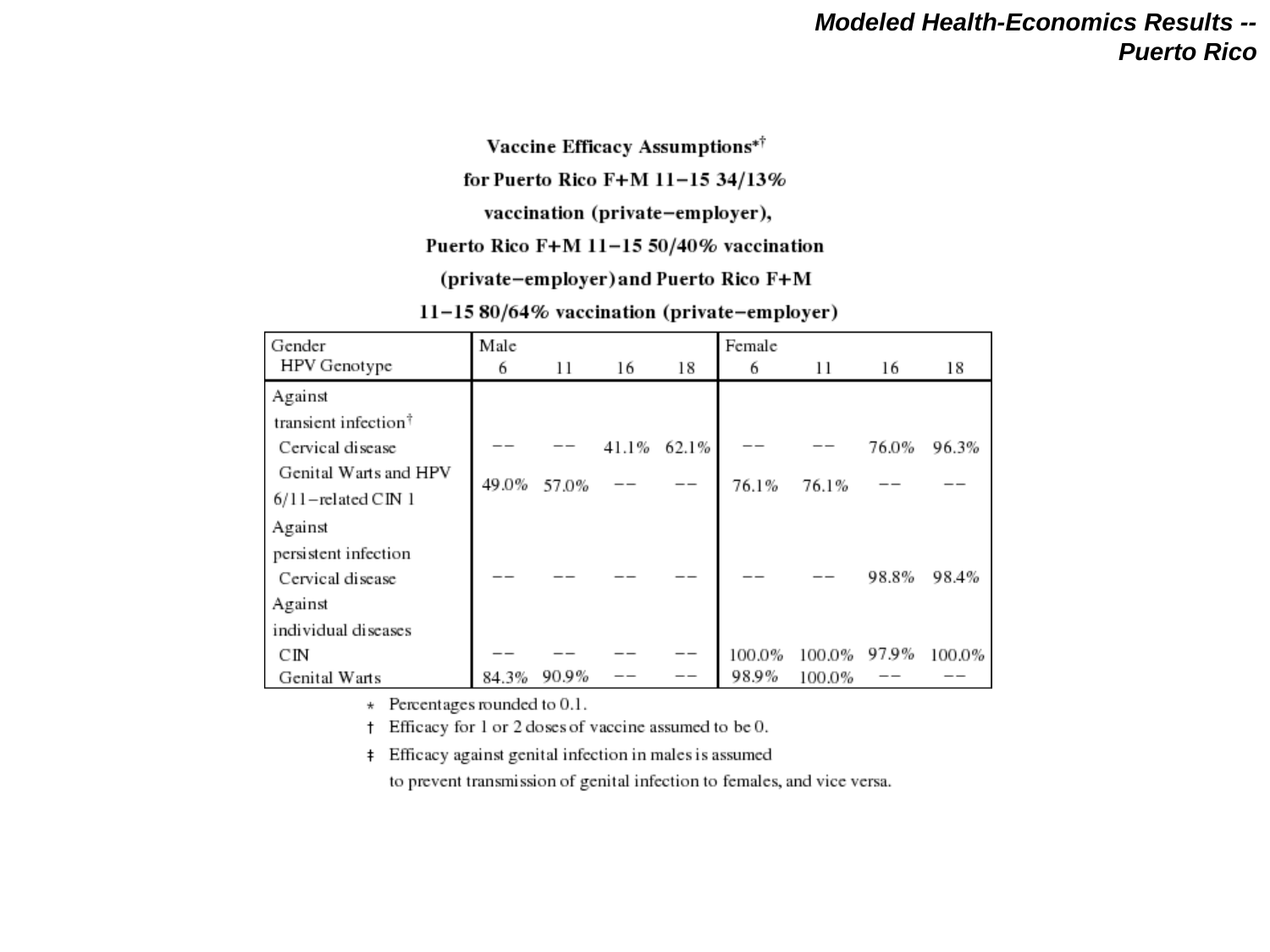

Modeled Health-Economics Results -- Puerto Rico
#
